# Microbiota in cancer: current understandings and future perspectives

**DOI:** 10.1038/s41392-025-02335-3

**Published:** 2026-02-02

**Authors:** Yanxi Yao, Yifei Zhu, Keji Chen, Jiayu Chen, Yuxue Li, Dawei Li, Ping Wei

**Affiliations:** 1 Department of Pathology, Fudan University Shanghai Cancer Center, Shanghai, China; 2 Cancer Institute, Fudan University Shanghai Cancer Center, Shanghai, China; 3 Institute of Pathology, Fudan University, Shanghai, China; 4 Department of Oncology, Shanghai Medical College Fudan University, Shanghai, China; 5 Department of Colorectal Surgery, Fudan University Shanghai Cancer Center, Shanghai, China

**Keywords:** Cancer microenvironment, Cancer metabolism, Tumour immunology, Cancer therapy

## Abstract

The intricate relationship between the microbiota and cancer has recently emerged as a pivotal area of research, highlighting their critical roles in carcinogenesis, progression, and prognosis. With the increasing recognition of the therapeutic potential of the microbiota in cancer, there is an urgent need to understand the diverse impacts of different microbiota on tumors and explore innovative strategies to harness their benefits. For the first time, this review traces the historical evolution of microbiota–cancer studies, from early observations of microbial presence in cancers to landmark discoveries linking specific microorganisms to carcinogenesis. Furthermore, this study delves into the molecular mechanisms underlying microbiota-mediated cancer progression to elucidate the modulatory roles of oncogenic pathways, immune responses, and tumor metabolism. We also discuss the dual roles of the microbiota in promoting and inhibiting cancer, highlighting its potential as both a facilitator of tumor growth and a target for therapeutic intervention. In addition, this review highlights the mechanism by which the microbiota mediates the response to anticancer immunotherapy, chemotherapy, and radiotherapy. Simultaneously, emerging anticancer strategies targeting microbiota (e.g., probiotics, antibiotics, and fecal microbiota transplantation) have been explored alongside U.S. Food and Drug Administration-approved drugs and ongoing clinical trials. Finally, this review outlines future directions in this field, emphasizing the need for personalized approaches to harness the anticancer potential of the microbiota. The interpretations in this review are expected to establish a stereoscopic, comprehensive framework for advancing research and clinical applications in microbiota-targeted oncology.

## Introduction

Over the past century, there has been significant evolution in the study of the microbiota in cancer, renewing our understanding of the influence of microbial communities on human health and diseases. Early observations documented the presence of microorganisms in tumor tissues, but their roles in cancer biology have remained speculative for decades. Landmark discoveries, such as the identification of *Helicobacter pylori* (*H. pylori*), as causative agents in gastric cancer, revealed that specific microbes have a direct impact on carcinogenesis through inflammation, genotoxicity, and immune modulation. These findings in relevant fields have increased the interest of researchers in exploring diverse microbiota‒cancer interactions. At present, the microbiota is recognized as a key regulator of the tumor microenvironment (TME), which is capable of driving or inhibiting cancer progression through its generous regulatory roles in host pathways, immune responses, and metabolic processes. In this context, the microbiota are recognized for their dual nature—functioning both as “accomplices” in cancer progression and as potential “allies” in therapeutic interventions. This duality endows them with remarkable complexity and significance in the field of oncology.^1,2^ This highlights the importance of investigating potential mechanisms underlying the cancer-promoting and therapeutic anticancer roles of the microbiota. These findings may provide opportunities to deepen our understanding of cancer biology and identify novel approaches for cancer treatment. Accordingly, the present review provides a comprehensive consideration of the microbiota in cancer, beginning with the historical development of this field and the classification of tumor-associated microbiota. This review continues to explore their molecular mechanisms, roles within the TME, and impacts on cancer treatment outcomes. We also discuss emerging anticancer strategies targeting microbiota and provide several perspectives on future research directions to advance the potential of microbiota-based interventions in precision oncology.

## Research history and milestone events of the microbiota in cancer

**Fig. 1:**
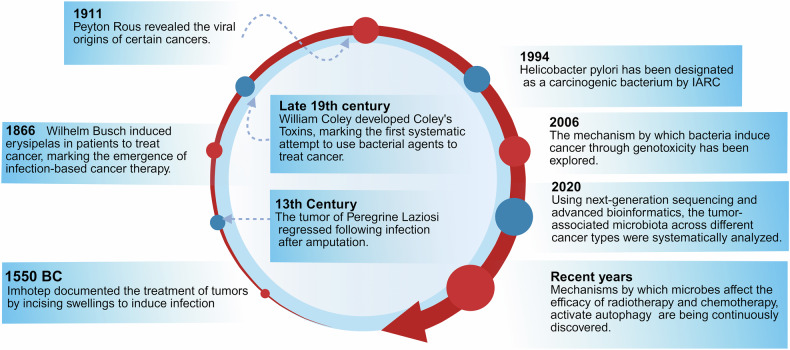
Timeline of the history and milestones in microbiota research in cancer. The figure highlights nine critical milestones in the study of tumor-associated microbiota, tracing the journey from the initial exploration of the relationship between microbes and cancer to practical applications. As technology has advanced, the functions of these microbiota have become increasingly clear, and the underlying mechanisms are gradually being elucidated. IARC International Agency for Research on Cancer. created in BioRender. Wang, Y. (2025) https://BioRender.com/w5er4m9

The history of studies on the microbiota in cancer has extended back millennia. Related observations and milestones can provide valuable references for the gradual evolution of our understanding of the roles of the microbiota in cancer (Fig. 1).

### Early observations and initial studies

As early as 1550 BCE, Imhotep, an Egyptian physician, documented the treatment of tumors through the incision of swellings to provoke infection. This practice, although rudimentary, exemplified an early but intuitive approach to modulate immunity via microbial exposure.^3,4^ In the 13th century, Peregrine Laziosi, an Italian saint, suffered from a substantial tumor on his tibia, which resolved following a severe infection after amputation. To a certain extent, this infection might elicit an immune response, culminating in spontaneous tumor regression. Laziosi was designated as the patron saint of those suffering from dreaded diseases such as cancer, underscoring the enduring historical link between infection and tumor remission.^5^

By the 18th century, it had gained wider recognition concerning spontaneous tumor regression in the context of severe bacterial infections. Physicians noted that such infections frequently coincided with tumor shrinkage, leading to speculation regarding their therapeutic potential.^6^ In 1866, Wilhelm Busch deliberately induced erysipelas, a bacterial skin infection, in a cancer patient, resulting in substantial tumor regression.^7^ This pioneering work was further expanded upon by Friedrich Fehleisen, who isolated *Streptococcus pyogenes* (*S. pyogenes*) and confirmed the above findings, marking the advent of infection-based cancer therapies.^8^

In the late 19th century, the proposed concept was further advanced by William Coley, the father of cancer immunotherapy.^9^ He developed “Coley’s toxins (also known as Coley’s vaccine)”, which is composed of inactivated *S. pyogenes* and *Serratia marcescens*, and directly administered this vaccine into sarcomas.^10^ Consequently, this procedure achieves tumor regression in some cases, marking the first systematic attempt to leverage bacterial agents for cancer treatment.^11^ However, the mechanisms underlying these effects remain obscure. At that time, Coley’s work illuminated the potential role of the immune system in combating malignancies, laying the foundation for contemporary cancer immunotherapy.^12^

In the early 20th century, Thomas Glover and Virginia Livingston-Wheeler proposed the controversial hypothesis that bacteria cultured from tumors have a common bacterial etiology for cancer.^7,13^ Although ultimately refuted, their studies emphasized the growing interest in microbial contributions to increase the development of cancer biology. In 1911, Peyton Rous demonstrated that chicken tumor-derived cell-free filtrates could transmit sarcomas, revealing a viral origin for some types of cancers.^14^ This landmark discovery paved the way for identifying oncogenic viruses, including Epstein–Barr virus (EBV), human papillomavirus (HPV), and hepatitis B and C viruses, all of which have since been implicated in the pathogenesis of various malignancies.^15,16^

### Key discoveries in the modern era

In 1983, Barry Marshall and Robin Warren successfully cultured *H. pylori* from the gastric mucosa of patients with chronic gastritis and peptic ulcers. This seminal discovery highlights the prevailing notion that stress and dietary habits primarily induce peptic ulcer diseases, revealing instead a bacterial etiology.^17^ Subsequent epidemiological and experimental studies firmly established a direct link between *H. pylori* infection and gastric adenocarcinoma, leading to its designation in 1994 as the first bacterium classified as a Group 1 carcinogen by the International Agency for Research on Cancer.^18^ In addition to providing a causative explanation for gastric cancer, this groundbreaking finding also revolutionized its treatment. The use of *H. pylori*-targeted antibiotic regimens has contributed to a significant reduction in the incidence of gastric ulcers and related malignancies, highlighting the therapeutic potential of combating pathogenic microbes.^19^

The discovery of *H. pylori* spurred broader exploration of the role of microbial agents in carcinogenesis.^20^ Research has subsequently revealed that *Fusobacterium nucleatum* (*F. nucleatum*) contributes to colorectal cancer and that *Porphyromonas gingivalis* (*P. gingivalis*) contributes to oral cancer.^21,22^ By 2006, existing research revealed a direct mechanism by which certain bacterial species promote cancer through genotoxic effects.^23^ Strains of *Escherichia coli* (*E. coli*), which produce cytolethal distending toxins, for example, were shown to induce DNA double-strand breaks in host cells.^24^ By disrupting DNA repair mechanisms and promoting mutagenesis, these genotoxic effects could promote genomic instability, a hallmark of cancer.^25^ This paradigm shifted the focus from chronic inflammation as the primary mode of microbial influence to the direct DNA-damaging capacity of bacterial toxins.^26^ Discoveries of other genotoxins (e.g., colibactin) have further increased the understanding of bacterial metabolites in cancer biology.^26^

Recent studies have increasingly illuminated the diverse roles of the microbiota in carcinogenesis, progression, and therapeutic response.^27,28^ Landmark studies have demonstrated the significant regulatory role of the gut microbiota in the efficacy of immune checkpoint inhibitors (ICIs).^29–31^ Notably, by priming the immune system to combat tumors more effectively, enhanced responses to ICIs have been revealed to be linked to the presence of *Akkermansia muciniphila* (*A. muciniphila*) and *Enterococcus hirae*.^32^ The composition of the gut microbiota has also been reported to affect the efficacy and toxicity of chemotherapeutic agents.^33^ Specific bacterial species, such as *F. nucleatum*, metabolize chemotherapeutic agents to alter their effectiveness.^34^ In colorectal cancer, *F. nucleatum* might trigger chemoresistance by interfering with the efficacy of 5-fluorouracil and oxaliplatin to activate autophagy pathways in cancer cells.^35,36^ Therefore, microbiota profiling holds promise for personalizing cancer treatment by identifying bacterial species that may influence drug response and resistance.^37^

Technological advances have further challenged the long-held belief that tumors are sterile environments, as assumed by previous research on cancer. High-resolution sequencing and microbial imaging have revealed diverse microbial communities within tumors, emphasizing their roles in shaping cancer biology. For example, *F. nucleatum*, which is found frequently in colorectal cancer tissues, was shown to modulate immune responses and induce treatment resistance.^38^ In 2020, large-scale studies systematically analyzed the tumor-associated microbiota across cancer types via next-generation sequencing and advanced bioinformatics.^39,40^ Specifically, Poore et al. profiled microbial DNA in more than 30 cancer types, identifying distinct microbial signatures associated with specific tumors. These findings support the potential use of microbial DNA as a diagnostic biomarker with high sensitivity and specificity. Concurrently, Straussman et al. provided spatial imaging evidence of intratumoral microbial localization, elucidating the interactions of microbes with cancer cells and their impact on the TME.^41^ Building on these insights, a significant role for fungal communities in the TME has been demonstrated to reveal a polymicrobial network within cancers. Fungal profiling across 35 tumor types identified species such as *Candida* and *Malassezia*, which are associated with gastrointestinal cancer and pancreatic cancer, respectively. These fungi synergize with bacteria to promote cancer progression and modulate immune responses. The correlations between fungal DNA signatures and clinical outcomes underscore their potential impacts on therapeutic efficacy and treatment resistance.^42^

Taken together, these milestones highlight the intricate interplay between microbial communities and tumors, advancing development in the field of cancer microbiology. Existing and future studies may provide more useful data to explore microbiome-targeted therapies through the identification of cancer-specific microbial signatures and the elucidation of their functional roles. Such approaches may include modulating microbial populations to increase therapeutic efficacy or employing microbiome-based diagnostics for early detection and prognosis, offering promising avenues to improve cancer outcomes.

## Classifications of microbiota in cancer

**Fig. 2:**
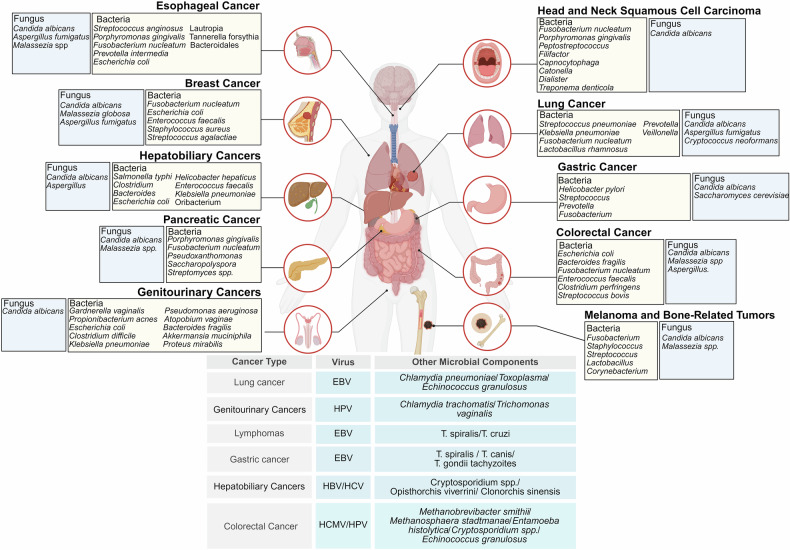
Microbial colonization in tumors. Bacteria, fungi, viruses, and parasites can all colonize tumors in various locations, thereby promoting or inhibiting tumor progression. The microbial compositions of different types of tumors exhibit a certain degree of heterogeneity. EC esophageal cancer, BC breast cancer, PC pancreatic cancer, HNSCC head and neck squamous cell carcinoma, LCA lithocholic acid, GC gastric cancer, CRC colorectal cancer. Created in BioRender. Wang, Y. (2025) https://BioRender.com/kvzbzyn

The microbiota in cancer represents a complex and dynamic ecosystem, encompassing a diverse spectrum of microorganisms, e.g., bacteria, fungi, viruses, archaea, and protozoa.^43,44^ Each microbial group contributes distinctively to the intricate interactions within the TME, shaping critical processes such as cancer initiation, progression, metastasis, and therapeutic response^44^ (Fig 2). These microorganisms engage in multifaceted interactions with both tumor cells and the host immune system, thereby mediating local and systemic metabolism and modulating pathways central to tumor behavior.^45,46^ The multifarious roles of the microbiota in cancer underscore its dualistic nature, serving both as potential drivers of carcinogenesis and as promising targets for therapeutic intervention.

### Bacterial microbiota in cancer

The bacterial microbiota plays a multifaceted role in carcinogenesis, progression, and therapeutic responses.^47^ Recent studies have elucidated the complex interactions between bacterial communities and cancer biology, highlighting both their oncogenic and antioncogenic potential.^48^ Cancer-associated bacteria originate from diverse sources and are influenced by anatomical sites, physiological barriers, and environmental conditions.^7,49^ These bacteria can infiltrate the TME through breaches in mucosal barriers, migration from adjacent tissues, or systemic dissemination.

(a) The mucosal barrier serves as the first barrier against microbial invasion.^50^ When compromised, it may create a gateway for bacteria to infiltrate tissues and induce carcinogenesis.^51^ The gastrointestinal tract, which harbors a dense microbial community, forms a protective mucosal barrier that is critical for maintaining internal homeostasis.^52^ Disruption of this barrier may provide bacteria with free access to underlying tissues to trigger cancer.^53^ For example, *H. pylori* breaches the gastric mucosa and is recognized as a key driver of gastric cancer by initiating chronic inflammation and DNA damage.^54^ Similarly, dysbiosis in the gut microbiota can stimulate the overgrowth of pathogenic *E. coli* that produce colibactin, a genotoxin implicated in colorectal cancer.^55^ Periodontal disease and poor oral hygiene can compromise the mucosal barrier in the oral cavity, facilitating the translocation of bacteria such as *F. nucleatum* to distant sites, including the colon.^56^ Moreover, *F. nucleatum* can adhere to and invade colonic epithelial cells, exacerbating colorectal carcinogenesis by activating β-catenin signaling.^57^ Chronic respiratory infections may enable bacterial infiltration by disturbing the epithelial barrier of the airway.^58^ Persistent infection with *Chlamydia pneumoniae* and subsequent inflammation may create a permissive environment for the development of lung cancer.^59^ (b) Cancer-associated bacteria may also originate from adjacent anatomical regions and migrate into neighboring tissues through direct extension or inflammation-mediated pathways.^60^ The gallbladder is a reservoir for *Salmonella typhi* (*S. typhi*), particularly in chronic carriers. Persistent colonization by *S. typhi* and inflammation of the biliary epithelium are significant risk factors for gallbladder cancer.^61^ The presence of *S. typhi* in the biliary tract may also induce oxidative stress and DNA damage to cause carcinogenesis.^62^ Bacteria from the gut lumen can migrate to the peritoneum and other adjacent tissues, especially during mucosal barrier breaches caused by colorectal cancer.^63^ *Bacteroides fragilis* (*B. fragilis*), known for its enterotoxigenic strains, produces *B. fragilis* toxin (BFT), which disrupts epithelial cell junctions and promotes inflammation-driven carcinogenesis in colorectal tissues.^64^ Vaginal dysbiosis has been linked to cervical and ovarian cancers.^65^ *Gardnerella vaginalis* (*G. vaginalis*), a common bacterial species involved in bacterial vaginosis, for example, may create a conducive environment for carcinogenesis owing to epithelial inflammation and immune modulation.^66^ (c) Bacteria can enter the circulatory or lymphatic system and spread systemically to colonize distant tissues, including tumors.^67^ Systemic dissemination of bacteria via the bloodstream is a well-documented mechanism. For example, *Streptococcus anginosus* (*S. anginosus*), an oral microbiota, has been identified in esophageal cancer and liver cancer tissues.^68^ Through the bloodstream, *S. anginosus* may spread and interact with preexisting inflammation in the TME to promote carcinogenesis.^69^ Similarly, *F. nucleatum* may spread hematogenously to breast cancer and colorectal cancer, exacerbating their progression.^70^ Bacterial dissemination may also occur through the lymphatic system.^71^ Bacteria such as *Mycoplasma* species exploit immune cells such as macrophages to evade detection and utilize the lymphatic network for systemic spread. These bacteria may indirectly affect tumor progression by modulating immune responses in the TME.^72,73^ (d) Tumors, characterized by hypoxia, nutrient availability, and immune suppression, often create conditions favorable for bacterial colonization.^74,75^ The presence of hypoxic cores, one of the most defining features of a tumor, can provide an ideal niche for anaerobic bacteria such as *Clostridium* species. These bacteria thrive in low-oxygen conditions and can interact with tumor cells to promote angiogenesis and tumor growth.^75^ Tumor-induced immunosuppression allows bacteria to evade host defenses. For example, *F. nucleatum* may suppress T-cell activity within the TME, thus improving its own survival and facilitating tumor progression.^34^ Moreover, tumors can release metabolites such as lactate to support bacterial growth.^76^ Metabolically active bacteria within the TME can further modify local conditions to interfere with cancer biology. (e) External factors (e.g., diet, antibiotic use, chronic infections, etc.) may also alter the composition and behavior of cancer-associated bacteria.^77,78^ Specifically, diet plays a critical role in shaping the microbiota. High-fat diets might increase the risk of colorectal cancer by increasing the prevalence of pathogenic bacteria such as *E. coli* and *B. fragilis*.^79^ The overuse of antibiotics may disrupt microbial homeostasis, often leading to dysbiosis and the overgrowth of pathogenic bacteria. This disruption has been reported to alter responses to cancer therapy and increase bacterial colonization of tumors.^80^ Persistent bacterial infections, such as those caused by *H. pylori* and *S. typhi*, may create a chronic inflammatory environment that predisposes patients to the malignant transformation of specific tissues. These infections act as long-term sources of bacteria associated with cancer.^81,82^

#### Head and neck squamous cell carcinoma (HNSCC)

HNSCC is closely associated with dysbiosis of the oral microbiota and is characterized by an enrichment of pathogenic bacteria such as *F. nucleatum* and *P. gingivalis* and a depletion of commensal bacteria such as Streptococcus species. These shifts in microbial composition may contribute to carcinogenesis through multiple mechanisms.^83,84^ *F. nucleatum*, which is frequently detected in HNSCC patients, can promote carcinogenesis by invading epithelial cells, activating β-catenin signaling, and suppressing antitumor immune responses through the upregulation of immune checkpoint molecules such as PD-L1.^57,85^ Similarly, *P. gingivalis*, a key pathogen in periodontal disease, can induce chronic inflammation and modulate the TME by activating the nuclear factor-kappa B (NF-κB) and STAT3 pathways, promoting epithelial cell proliferation and inhibiting apoptosis.^86,87^

The proinflammatory milieu generated by these bacteria generally presents elevated levels of cytokines (e.g., IL-6 and TNF-α), which facilitate angiogenesis and tumor progression.^88^ Certain bacterial species can also produce genotoxins or reactive oxygen species (ROS) to induce DNA damage and genomic instability, further driving carcinogenesis.^89^ These mechanistic insights underscore the importance of the oral microbiota in HNSCC development. Clinically, alterations in the composition of the microbiota, including increased abundances of *F. nucleatum* and *P. gingivalis*, may greatly benefit the diagnosis of HNSCC at an early stage.^90^ Moreover, HNSCC can be prevented or managed through promising therapeutic strategies that target dysbiotic oral microbiota, such as probiotics or microbiota-modulating agents.^91^ In this context, continuing research on deciphering the interplay between the microbiota, host immune responses, and environmental factors such as tobacco and alcohol is important. This study may provide deeper insights into the various functions of bacterial communities in HNSCC and facilitate the development of innovative diagnostic and therapeutic approaches.

#### Esophageal cancer

Esophageal cancer generally includes two major clinical phenotypes, esophageal squamous cell carcinoma (ESCC) and esophageal adenocarcinoma, which are significantly associated with alterations in the esophageal microbiota.^92^ Dysbiosis in esophageal cancer patients is usually characterized by decreased microbial diversity and the dominance of specific pathogenic species, such as *S. anginosus*, *P. gingivalis*, and *F. nucleatum*.^93^ In addition to colonizing the esophageal mucosa, these bacteria may also actively modulate the TME to promote carcinogenesis through multiple mechanistic pathways.

*S. anginosus*, a frequently occurring bacterium in ESCC tissues, can initiate carcinogenesis by inducing chronic inflammation and producing carcinogenic metabolites.^94^ Persistent colonization by *S. anginosus* may trigger an inflammatory cascade involving the upregulation of IL-6, TNF-α and other cytokines to induce cell proliferation and angiogenesis.^95^ Moreover, this bacterium can generate ROS, causing DNA damage and increasing genomic instability in esophageal epithelial cells.^96^

*P. gingivalis*, which is commonly associated with periodontal disease, has also been implicated in ESCC.^97^ Its virulence factors, such as gingipains, allow it to degrade host proteins, disrupt cellular adhesion, and invade epithelial cells.^98^ By altering T-cell activity, *P. gingivalis* can suppress antitumor immune responses to promote immune evasion in the TME.^99^ Furthermore, *F. nucleatum*, which is enriched in both ESCC and esophageal adenocarcinoma tissues, can play an oncogenic role through its adhesin protein FadA.^100^ By binding to E-cadherin on epithelial cells, FadA can promote the activation of β-catenin pathways to increase cell proliferation and migration.^101^ Moreover, bacterial dysbiosis in esophageal adenocarcinoma may be related to the occurrence of gastroesophageal reflux disease and its precursor lesion, Barrett’s esophagus. The esophageal microbiota in esophageal adenocarcinoma patients has shifted toward an increased prevalence of gram-negative bacteria, such as *Prevotella intermedia* and *E. coli*, thereby exacerbating reflux-induced inflammation and oxidative stress.^102^ These bacteria release lipopolysaccharides to activate Toll-like receptor (TLR) 4 signaling, further amplifying inflammatory and oncogenic pathways.^103^

Collectively, the esophageal microbiota can be considered a pathologic contributor to esophageal cancer development by inducing/boosting chronic inflammation, generating genotoxic agents, activating oncogenic pathways, and modulating immune responses. These microbial signatures may serve as potential biomarkers for early detection and risk stratification. Furthermore, esophageal cancer patients may benefit from the benefits of management and outcomes of therapeutic strategies that target microbial dysbiosis, including probiotics, antibiotics, or microbiota transplantation.^104^ At this stage, further studies are still warranted to fully elucidate the causal relationships and optimize microbiota-based interventions.

#### Breast cancer

Recent data have established an association of breast cancer with dysbiosis in both the gut and breast tissue microbiota, highlighting the value of microbial communities in its development and progression. The gut microbiota is particularly relevant to hormone receptor-positive breast cancer considering its roles in systemic inflammation, hormone metabolism, and immune regulation.^105^ Certain bacterial species, such as those involved in estrogen metabolism (e.g., *E. coli* and *Enterococcus faecalis* (*E. faecalis*)), can produce β-glucuronidase, an enzyme that deconjugates estrogen metabolites in the gut, leading to increased circulating estrogen levels.^106,107^ Critically, elevated estrogen is a well-known risk factor for breast cancer.^108^

In addition to the gut, breast tissue harbors its own microbiota, which is distinct from that of other body sites. An increased abundance of *Staphylococcus*, *Streptococcus*, and *E. coli* species has previously been identified in breast cancer tissues compared with healthy controls.^109^ *E. coli* strains isolated from breast cancer tissues can produce colibactin, a genotoxin that induces DNA double-strand breaks, contributing to genomic instability and carcinogenesis.^110^ Similarly, *Staphylococcus aureus* and *Streptococcus agalactiae* are thought to trigger local inflammation and immune modulation within the TME. The microbial dysbiosis-induced inflammatory environment can suppress antitumor immune responses and promote angiogenesis to initiate tumors and support their progression.^111^ For example, gram-negative bacteria-derived lipopolysaccharides may activate TLR4 signaling to increase tumor cell proliferation, migration, and invasion.^112^ Moreover, alterations in the microbiota within the breast tissue may result in abnormal local immune cell activity and drug metabolism, eventually compromising the efficacy of certain cancer treatments, including chemotherapy and immunotherapy.^113^ Therefore, targeting microbiota-associated pathways is a potential therapeutic strategy. Probiotic interventions to restore microbial balance or dietary modifications to influence the gut microbiota composition are being explored as adjunctive therapies for breast cancer.^114^ Additionally, it may be feasible to mitigate tumor-promoting effects by using antibiotics to selectively target pathogenic bacterial species within breast tissues. Future research is needed to fully elucidate the interactions between the microbiota, systemic hormone levels, and the breast TME, paving the way for innovative diagnostic and therapeutic approaches in breast cancer.

#### Lung cancer

Lung cancer, including non-small cell lung cancer (NSCLC) and small cell lung cancer, has been increasingly linked to dysbiosis of both the lung and gut microbiota.^115^ The lung, previously considered sterile, harbors a diverse microbial community that interacts with the TME to initiate and advance cancer.^116^ The abundance of pathogenic bacteria such as *Streptococcus pneumoniae* (*S. pneumoniae*), *Klebsiella pneumoniae* (*K. pneumoniae*) and *F. nucleatum* has been shown to be increased in LC patients, along with a reduction in the abundance of commensal species.^117,118^ The presence of these pathogenic bacteria in the lung may contribute to carcinogenesis through several mechanisms. *F. nucleatum*, known for its role in colorectal cancer, is also implicated in lung cancer. It can promote immune evasion by recruiting myeloid-derived suppressor cells (MDSCs) and suppressing T-cell-mediated antitumor responses.^119^ Similarly, *S. pneumoniae* can exacerbate local inflammation, releasing proinflammatory cytokines (e.g., IL-6 and TNF-α), which create a protumorigenic environment.^120^ In addition to local effects, the gut‒lung axis has emerged as a key pathway in lung cancer progression. Dysbiosis of the gut microbiota can disturb systemic immune responses and the lung TME. For example, gut bacteria such as *B. fragilis* and *E. faecalis* produce metabolites such as short-chain fatty acids (SCFAs) that regulate immune cell activity. An altered gut microbiota composition may impair the activation of dendritic cells and cytotoxic T lymphocytes in the lungs to reduce the efficacy of immunotherapies.^121^ Additionally, bacterial biofilms within the lungs can facilitate chemoresistance by protecting tumor cells from drug penetration and altering drug metabolism.^122^

From a therapeutic perspective, therapeutic potential can be triggered by modulating the lung and gut microbiota. Currently, probiotics, prebiotics, and fecal microbiota transplantation (FMT) are being investigated as adjunctive therapies to restore microbial balance and enhance antitumor immunity.^123^ Antibiotics targeting specific pathogens, such as *K. pneumoniae*, may also relieve bacteria-driven inflammation and improve therapeutic efficacy.^124^ Future studies should focus on elucidating the interplay between the lung and gut microbiota, host immune responses, and cancer biology to optimize microbiota-targeted therapies for lung cancer.

#### Gastric cancer

Gastric cancer is among the top causes of cancer-related mortality worldwide, and its development is strongly associated with the gastric microbiota.^125^ While *H. pylori* is the most well-established bacterial contributor to gastric cancer, emerging research suggests that non-*H. pylori* bacteria is also a key participant in gastric carcinogenesis. These microorganisms collectively interact with the host to create a tumor-promoting environment through inflammation, immune modulation, and metabolic alterations.^126^

The virulence factors of *H. pylori*, such as CagA and VacA, disrupt epithelial integrity, induce DNA damage, and alter cellular signaling. Specifically, CagA is injected into gastric epithelial cells via a type IV secretion system, where it activates oncogenic pathways such as the NF-κB and Wnt/β-catenin pathways to promote cell proliferation and survival.^127^ On the other hand, VacA induces mitochondrial dysfunction and resistance to apoptosis. Together, these effects can create a proinflammatory environment characterized by the release of cytokines such as IL-6 and TNF-α, thereby driving the development of chronic gastritis and increasing the risk of neoplastic transformation.^128^ Persistent *H. pylori* infection is also associated with epigenetic changes, including hypermethylation of tumor suppressor genes, further contributing to malignancy.^81^ In addition to *H. pylori*, alterations in the gastric microbiota of gastric cancer patients have also been documented in recent studies. The enrichment of bacterial genera, e.g., *Streptococcus*, *Prevotella*, and *Fusobacterium*, has been observed in gastric tumor tissues.^69,129^ These non-*H. pylori* bacteria may promote carcinogenesis through distinct mechanisms. For example, certain bacteria can metabolize dietary nitrates into carcinogenic N-nitrosamines, which can damage DNA and initiate tumor development.^130^ Others, such as *F. nucleatum*, can also facilitate tumor progression, as they can modulate the TME by recruiting tumor-associated macrophages (TAMs) and suppressing antitumor immune responses. Dysbiotic microbiota may also disrupt the gastric mucosal barrier, thus increasing susceptibility to carcinogenic insults and further increasing the risk of cancer.^131^

To improve prevention and treatment strategies for gastric cancer in the clinic, a deeper understanding of the interplay between *H. pylori* and non-*H. pylori* is needed. pylori microbiota. To date, eradication of *H. pylori* has not addressed the potential contributions of other bacterial species, despite its possible role in reducing the risk of gastric cancer.^132^ Emerging therapeutic strategies, such as probiotics, prebiotics, and microbiota-targeting drugs, aim to restore healthy gastric microbiota balance.^132^ Concerning future research directions, emphasis should be placed on delineating the roles of individual microbial species in gastric carcinogenesis as well as developing integrated microbiota-based diagnostic and therapeutic approaches for gastric cancer.

#### Hepatobiliary cancers

Hepatobiliary cancers generally include hepatocellular carcinoma, cholangiocarcinoma, and gallbladder cancer. All these cancers are significantly influenced by the gut‒liver axis, wherein the gut microbiota and their metabolites interact with hepatic tissues to promote carcinogenesis.^133^ Dysbiosis of the gut microbiota may increase intestinal permeability, facilitating the translocation of microbial products such as lipopolysaccharides into the portal circulation. Lipopolysaccharides can further activate TLR4 signaling in hepatocytes and Kupffer cells, triggering chronic inflammation and the release of proinflammatory cytokines (e.g., IL-6 and TNF-α) to drive carcinogenesis in hepatocellular carcinoma.^134^ Additionally, gut bacteria are also contributors to metabolic alterations in the bile acid pool. Certain species, including *Clostridium* and *Bacteroides*, can convert primary bile acids into secondary bile acids such as deoxycholic acid, which is hepatotoxic and carcinogenic.^135^ Deoxycholic acid can induce oxidative stress and DNA damage and upregulate protumorigenic pathways in hepatocytes to facilitate the progression of hepatocellular carcinoma. Deoxycholic acid also impairs antitumor immunity by suppressing CD8^+^ T-cell effector functions.^136^

In cholangiocarcinoma, bacterial infections, notably with *Helicobacter hepaticus* (*H. hepaticus*) and *E. faecalis*, have been implicated in carcinogenesis. These bacteria can activate oncogenic pathways, such as the NF-κB and STAT3 pathways, to induce biliary epithelial damage, chronic inflammation, and increased cell proliferation.^137^ Gut-derived secondary bile acids can also damage the biliary epithelium and initiate a regenerative response, increasing the risk of malignant transformation.^138^

Gallbladder cancer is a highly lethal malignancy that is associated with bacterial infections. Chronic carriage of *S. typhi* has been strongly linked to an increased risk of gallbladder cancer, particularly in endemic regions.^139^ Persistent colonization of the gallbladder epithelium by S. typhi may induce chronic inflammation, oxidative stress, and DNA damage, leading to epithelial dysplasia and thus malignant transformation.^139^ Moreover, dysbiosis has been identified in the gallbladder microbiota of gallbladder cancer patients. There have been reports related to the enrichment of pathogenic species such as *K. pneumoniae* and *E. coli* in the gallbladder.^140^ These bacteria may produce proinflammatory metabolites and disrupt the epithelial barrier to promote gallbladder cancer development. Additionally, gut-derived secondary bile acids, such as lithocholic acid, can accumulate in the gallbladder and exert carcinogenic effects on the biliary epithelium.^141^

Targeting the microbiota may offer potential therapeutic avenues for gallbladder cancer. Antibiotics to eliminate *S. typhi* in chronic carriers may reduce the risk of gallbladder cancer in high-risk populations.^139^ Moreover, probiotics and dietary modifications can be regarded as preventive strategies to modulate bile acid metabolism, which are currently being investigated.^142^ Further research is needed to elucidate the complex interactions among bacterial infections, bile acid dysregulation, and gallbladder carcinogenesis to offer additional insights for microbiota-based interventions.

#### Pancreatic cancer

Pancreatic cancer, particularly pancreatic ductal adenocarcinoma (PDAC), is a highly aggressive malignancy with a poor prognosis. Emerging evidence indicates that the microbiota, which includes oral, gut, and tumor-resident bacteria, plays a vital role in pancreatic carcinogenesis through mechanisms involving inflammation, immune modulation, and metabolic alterations.^143^

In terms of the role of the oral microbiota in pancreatic cancer, periodontal pathogens such as *P. gingivalis* and *F. nucleatum* have been revealed to increase the risk of pancreatic cancer.^144^ These bacteria can translocate from the oral cavity to the pancreas, potentially via hematogenous routes, and colonize themselves within pancreatic tissue. Once localized, they may induce chronic inflammation and modulate the TME by suppressing antitumor immune responses, thereby promoting carcinogenesis.^144^ The gut microbiota is also an important culprit that cannot be ignored in pancreatic cancer. Dysbiosis, characterized by an imbalance in the microbial composition of beneficial and pathogenic bacteria, can increase intestinal permeability to accelerate the translocation of microbial products such as lipopolysaccharides into the systemic circulation.^145^ Within the pancreatic TME, certain bacteria may also participate in disease progression by directly interacting with tumor cells and immune cells. For example, *F. nucleatum* can modulate immune responses by inhibiting the activity of natural killer (NK) cells and cytotoxic T lymphocytes, facilitating immune evasion by tumor cells.^146^ Additionally, the presence of specific bacterial populations within the pancreas can affect the efficacy of chemotherapeutic agents. For example, chemoresistance may develop since some bacteria possess enzymes capable of metabolizing gemcitabine (Gem), a standard chemotherapeutic agent for pancreatic cancer, into its inactive form.^147^

At present, some researchers in this field are exploring therapeutic strategies that target the microbiota to improve pancreatic cancer outcomes. Approaches such as probiotics, prebiotics, and FMT can be applied to restore healthy microbial balance, reduce inflammation, and enhance antitumor immunity. The use of selective antibiotics to deplete tumor-promoting bacteria is currently under investigation.^148^ The development of microbiota-based diagnostic and therapeutic strategies may benefit from a deeper understanding of the complex interactions between the microbiota and pancreatic cancer.

#### Colorectal cancer

Colorectal cancer is one of the most extensively studied cancers in the context of the gut microbiota. Dysbiosis may play a critical role in colorectal cancer development and progression owing to an imbalanced distribution of beneficial and pathogenic gut bacteria.^149^

*F. nucleatum* is frequently enriched in colorectal cancer tissues and is known to promote carcinogenesis through multiple mechanisms.^34^ It can adhere to colonic epithelial cells via its adhesin protein FadA, which binds to E-cadherin, activating β-catenin pathways that drive cell proliferation and tumor invasion.^101^ *F. nucleatum* also promotes an immunosuppressive TME by recruiting MDSCs while inhibiting the activity of cytotoxic T cells, enabling immune evasion by tumor cells.^144^ Another key player in colorectal cancer is *E. coli*, specifically strains harboring the polyketide synthase genomic island. These strains produce colibactin, resulting in genomic instability and the accumulation of mutations in colorectal tissues. This genotoxic effect is further exacerbated by the inflammatory environment created by dysbiosis.^150^ *B. fragilis*, particularly its enterotoxigenic strains, contributes to colorectal cancer by secreting BFT, a toxin that disrupts epithelial barrier integrity and induces chronic inflammation. BFT-mediated activation of the Wnt/β-catenin pathway is a potential mechanism that promotes epithelial cell proliferation and enhances carcinogenesis.^151^ Gut microbial metabolism also plays a pivotal role in colorectal cancer development. Dysbiotic microbiota convert dietary components into harmful metabolites, such as secondary bile acids and hydrogen sulfide, which induce oxidative stress and DNA damage in colonic epithelial cells. These metabolic byproducts further exacerbate the tumor-promoting effects of a dysbiotic microbiota.^152^

Therapeutically, the gut microbiota can be targeted to potentially prevent and treat colorectal cancer. Probiotic and prebiotic interventions can restore microbial balance and enhance antitumor immunity. FMT has also been explored to counteract dysbiosis in colorectal cancer patients.^142^ Understanding the roles of specific microbial species and their metabolites may offer opportunities to develop novel diagnostic biomarkers and therapeutic strategies tailored to individual microbial profiles. Therefore, these complex interactions can be further explored in future studies to optimize microbiota-targeted therapies for colorectal cancer.

#### Genitourinary cancers

In general, genitourinary cancers encompass malignancies of the urinary, male reproductive, and female reproductive systems. Alterations in the local and systemic microbiota may have noteworthy effects on these cancers.^153^ Through mechanisms such as chronic inflammation, immune modulation, and microbial metabolite production, dysbiosis in the urinary tract, genital region, and gut microbiota may contribute to the pathogenesis and progression of these cancers.^153^

The vaginal and cervical microbiota are key players in female reproductive cancers, particularly in the context of cervical and ovarian cancers. In cervical cancer, coinfection with *Chlamydia trachomatis* (*C. trachomatis*) has been shown to act synergistically with HPV to promote carcinogenesis. The presence of *C. trachomatis* may create a tumor-promoting microenvironment due to its ability to induce chronic inflammation, DNA damage, and immune evasion.^154^ Dysbiosis in the vaginal microbiota may present with a reduction in protective *Lactobacillus* species and an overgrowth of pathogenic anaerobes such as *G. vaginalis*. It may further exacerbate inflammation, thereby enhancing HPV persistence and integration into the host genome.^155^ Moreover, in ovarian cancer, emerging evidence suggests a potential role for *Mycoplasma* species in tumor initiation and progression. These bacteria can induce genomic instability and suppress apoptosis in ovarian epithelial cells. Additionally, the gut microbiota, through the gut‒ovary axis, may indirectly influence ovarian carcinogenesis by modulating systemic inflammation and estrogen metabolism.^156^

Furthermore, in male reproductive cancers, such as prostate cancer, dysbiosis of the prostate and gut microbiota has been linked to carcinogenesis. *Propionibacterium acnes* and *E. coli* are commonly detected in prostate cancer tissues because of their mechanisms of inducing chronic inflammation, oxidative stress, and DNA damage in prostatic epithelial cells.^156^ Dysbiosis in the urinary microbiota has also been reported in the carcinogenesis of urinary system cancers, particularly bladder cancer. Studies have consistently reported a reduction in protective *Lactobacillus* species as well as an increased abundance of *Proteobacteria*, including *E. coli*, *K. pneumoniae*, and *Pseudomonas aeruginosa* (*P. aeruginosa*).^157^ Following disruption of the urothelial barrier by damage to tight junctions, the presence of these pathogenic bacteria may increase susceptibility to carcinogenic insults. Moreover, these bacteria stimulate chronic inflammation through the release of cytokines (e.g., IL-6 and IL-8), creating a tumor-promoting microenvironment. Additionally, these bacteria also produce carcinogenic metabolites, such as reactive oxygen species (ROS) and nitrosamines, which contribute to DNA damage and genomic instability, key processes in bladder carcinogenesis.^158^ Microbial dysbiosis has also been implicated in renal cell carcinoma. To date, the direct association of renal cell carcinoma with the urinary microbiota is poorly understood. Existing studies suggest that the gut microbiota plays a significant role in renal cell carcinoma pathogenesis through immune modulation and metabolic regulation. Dysbiosis in renal cell carcinoma patients is usually characterized by an increased abundance of proinflammatory bacteria, such as *B. fragilis* and *Clostridium difficile* (*C. difficile*), and a depletion of beneficial species, such as *A. muciniphila*. These microbial alterations may lead to impaired gut barrier function, increased systemic inflammation, and the production of secondary bile acids, all of which accelerate renal carcinogenesis. Urinary tract squamous cell carcinoma, a rare but aggressive malignancy, has also been strongly associated with chronic bacterial infections. Pathogens such as *Schistosoma hematobium*-associated bacteria or recurrent *Proteus mirabilis* infections can cause persistent inflammation, urothelial hyperplasia, and eventual malignant transformation. Additionally, recurrent urinary tract infections are also suspected to contribute to carcinogenesis in urinary tract squamous cell carcinoma through the release of proinflammatory mediators and nitrosamines.^159^

Future research should prioritize elucidating the precise roles of the microbiota in genitourinary malignancies, particularly in the interactions of local and systemic microbiota with the TME. This understanding provides novel or additional insights for microbiota-targeted diagnostic tools and personalized therapeutic strategies.

#### Melanoma and bone-related tumors

Dysbiosis in both the skin and the gut microbiome can also interfere with the progression of melanoma. Melanoma tissues have been shown to have increased abundances of genera such as *Fusobacterium*, *Staphylococcus*, and *Streptococcus*, whereas healthy skin has increased levels of *Lactobacillus* and *Corynebacterium*. These microbial imbalances may disrupt local immune responses and amplify inflammation to trigger tumor development. Alterations in the gut microbiota have been associated with melanoma progression, suggesting a systemic interaction between distant microbial communities and skin cancer.^160,161^

Existing evidence has revealed links between the gut microbiome and bone metastasis in cancers such as breast and prostate cancer, despite insufficient direct studies on bacterial involvement in primary bone tumors. Disruptions in gut microbial communities can disturb bone remodeling and create a favorable environment for metastatic cancer cells. Mechanisms include the modulation of the immune system and alterations in the homeostasis of the bone microenvironment.

### Fungal microbiota in cancer

The fungal microbiota, collectively known as the mycobiome, represents a critical yet less-studied component of the human microbiome, with increasing evidence supporting its involvement in cancers. Fungal communities are present across various sites of the body and interact with other microbial populations, the host immune system, and the TME, resulting in both oncogenic and antioncogenic profiles.^162^ Similarly, dysbiosis in the mycobiome can influence carcinogenesis through mechanisms such as chronic inflammation, immune suppression, and metabolic reprogramming.^163^

#### Head and neck cancers

In HNSCC, *Candida albicans* (*C. albicans*) is a frequently occurring fungal species associated with chronic inflammation and immune evasion. By adhering to and invading mucosal surfaces, *C. albicans* may disrupt epithelial integrity and promote a protumorigenic environment. This invasion is facilitated by the fungus’s ability to transition from yeast to hyphal forms, increasing its pathogenicity.^83^ The hyphal forms secrete hydrolytic enzymes, such as proteases and phospholipases, which degrade epithelial barriers and extracellular matrix components, aiding in tissue invasion.^164^ The carcinogenic nature of *C. albicans* may be further highlighted, as it can produce acetaldehyde, a carcinogenic metabolite that can induce DNA damage in epithelial cells. The interaction of *C. albicans* with the host immune system is also important in HNSCC progression.^165^ The fungal cell wall component β-glucan can activate pattern recognition receptors on immune cells, such as dendritic cells and macrophages, triggering an inflammatory response characterized by the release of proinflammatory cytokines (e.g., IL-6 and TNF-α). Chronic inflammation creates a microenvironment conducive to tumor development by promoting cellular proliferation and survival.^166^ Moreover, *C. albicans* can modulate immune responses to favor tumor progression. It might cause dysfunction of natural killer cells and cytotoxic T lymphocytes, key players in antitumor immunity, thereby enabling tumor cells to evade immune surveillance. This immune modulation is, in part, mediated by the expression of fungal proteins that interfere with host immune pathways.^167^ The presence of *C. albicans* in the oral cavity has been demonstrated to be correlated with increased expression of genes associated with metastasis and epithelial‒mesenchymal transition in oral squamous cell carcinoma (OSCC) cells.^168^ Exposure to *C. albicans* in vitro upregulated the expression of matrix metalloproteinases, a family of endopeptidases that can degrade extracellular matrix components, facilitating tumor invasion and metastasis. *C. albicans* can also influence the metabolic profile of OSCC cells, promoting the production of oncometabolites that support tumor growth.^168,169^

In summary, *C. albicans* may be partially responsible for the pathogenesis of head and neck cancers through mechanisms involving epithelial invasion, induction of chronic inflammation, modulation of immune responses, and promotion of metastatic gene expression. Targeting *C. albicans* and its interactions with host tissues may offer additional solutions for preventing or mitigating the progression of HNSCC.

#### Esophageal cancer

*C. albicans* is one of the most frequently detected fungal species in the esophageal mucosa of esophageal cancer patients. This fungus adheres to damaged epithelial surfaces, exploiting disruptions caused by conditions such as gastroesophageal reflux disease and Barrett’s esophagus, both of which are precursors to esophageal adenocarcinoma.^170^ *C. albicans* adheres to epithelial cells through adhesins, such as Als3, and forms biofilms to exert a protective effect on the host immune system. These biofilms can produce ROS and acetaldehyde, metabolites known to cause DNA damage and genomic instability—key steps in carcinogenesis.^171^ In addition to its direct genotoxic effects, *C. albicans* can stimulate the release of proinflammatory cytokines, such as IL-6, IL-8, and TNF-α, from epithelial and immune cells to create a chronic inflammatory environment. In this environment, there may be epithelial cell proliferation and angiogenesis, both of which are critical for tumor growth and progression.^172^ In addition to *Candida* species, other fungi, including *Aspergillus* spp. and *Malassezia* spp., have been identified in esophageal cancer tissues. *Aspergillus fumigatus* (*A. fumigatus*) can produce aflatoxins, which are potent carcinogens that induce DNA adduct formation and inhibit DNA repair mechanisms. All these effects may result in the accumulation of mutations and the initiation of carcinogenesis. Moreover, *Malassezia* spp., which are traditionally associated with skin diseases, have recently been implicated in esophageal cancer by interacting with bacterial communities to enhance inflammatory responses.^173^

Clinically, fungal dysbiosis in the esophagus may be beneficial for the early detection of esophageal cancer. Importantly, antifungal treatments have shown promise in preclinical models of esophageal cancer because they reduce inflammation and retard tumor growth. Probiotic therapies targeting fungal‒bacterial interactions in the esophageal microbiota are also being investigated as potential strategies for preventing esophageal cancer in high-risk individuals, such as those with gastroesophageal reflux disease or Barrett’s esophagus.^74,174^

#### Breast cancer

Breast tissue, once thought to be sterile, harbors its own distinct microbiota, including fungal communities. An increased abundance of pathogenic fungi, such as *Malassezia globosa* (*M. globosa*) and *C. albicans*, has been detected in breast cancer tissues compared with healthy controls.^175^

*M. globosa* can interact with macrophages in breast tissue, polarizing them into tumor-promoting M2-like macrophages, thereby suppressing antitumor immunity and increasing angiogenesis.^176^ *C. albicans*, which is commonly present in the oral and gastrointestinal microbiota, may translocate to breast tissue under conditions of immunosuppression or systemic inflammation. In breast tissues, *C. albicans* secretes enzymes such as aspartyl proteases to degrade extracellular matrix components, thus facilitating tumor invasion and metastasis. Moreover, *C. albicans* can also produce acetaldehyde to trigger genomic instability in breast epithelial cells.^165,177^

Fungal-derived metabolites, such as SCFAs, also play nonnegligible roles in the immune landscape in breast cancer. Specifically, SCFAs produced by beneficial fungi may enhance antitumor immune responses, whereas dysbiosis can shift the fungal balance toward proinflammatory metabolites that promote tumor progression. Fungi within the breast TME can modulate immune responses to favor tumor growth. By interacting with pattern recognition receptors such as Dectin-1, fungal β-glucans can activate immune checkpoint pathways, leading to immune evasion by tumor cells. Fungal biofilms in breast tissue can also create a protective niche for both fungi and tumor cells, enhancing resistance to chemotherapy and immunotherapy.^178^

#### Lung cancer

The respiratory tract, which is traditionally considered sterile, harbors a diverse fungal community that can interact with the TME to influence cancer initiation, immune modulation, and progression. Pathogenic fungi such as *A. fumigatus*, *C. albicans*, and *Cryptococcus neoformans* (*C. neoformans*) have been detected in lung cancer tissues. Their presence is associated with inflammation, immune suppression, and metabolic alterations that favor tumor growth.^83,179^

Fungal dysbiosis in the lung is characterized by an increased abundance of pathogenic fungi, often accompanied by a reduction in commensal or beneficial species. *A. fumigatus* is frequently detected in lung cancer tissues and can produce aflatoxins that cause DNA damage and inhibit DNA repair mechanisms, leading to genomic instability. These aflatoxins also induce oxidative stress to trigger chronic inflammation, a critical driver of lung cancer carcinogenesis.^180^ Similarly, *C. albicans* may colonize damaged respiratory epithelia and form biofilms to promote lung cancer development. These biofilms protect fungal cells from immune clearance and provide a reservoir for the persistent production of ROS and acetaldehyde, which damage epithelial cells and create a protumorigenic environment.^181^ *C. neoformans*, another opportunistic fungal pathogen, is also involved in lung cancer progression by evading immune responses and inducing granulomatous inflammation, which disrupts normal tissue architecture and facilitates tumor growth. Fungi in the lung TME can suppress antitumor immunity through various mechanisms. Specifically, fungal cell wall components (e.g., β-glucans and mannans) can interact with pattern recognition receptors on immune cells, including dendritic cells and macrophages, to activate pathways such as Dectin-1 and TLR2 signaling. While these interactions can initially stimulate immune responses, persistent fungal exposure often leads to immune exhaustion or polarization toward tumor-promoting phenotypes. For example, fungal β-glucans can recruit regulatory T cells (Tregs) and MDSCs to the TME, both of which suppress cytotoxic T-cell activity and inhibit effective antitumor immunity.^182^ Fungi can also produce metabolites such as gliotoxins and ergosterol derivatives, which further dampen immune responses. Gliotoxins, for example, inhibit the activation of natural killer cells and induce apoptosis in lymphocytes, thereby reducing immune surveillance in lung cancer.^183^

Addressing fungal dysbiosis in the lung may offer potential therapeutic opportunities for lung cancer treatment. Antifungal agents targeting pathogenic fungi such as *A. fumigatus* and *C. albicans* are being explored as adjunctive therapies to alleviate inflammation and improve therapeutic outcomes.^184^ Fungal markers, such as β-glucans or specific fungal metabolites, can serve as diagnostic biomarkers for lung cancer or as predictors of treatment response. In addition, the integration of antifungal strategies with conventional lung cancer therapies, such as chemotherapy or immunotherapy, may provide additional ways to enhance therapeutic efficacy by mitigating fungal-mediated immune suppression and inflammation.^185^

#### Gastric cancer

Fungal microbiota, particularly species such as *Candida* spp. and *Saccharomyces cerevisiae* (*S. cerevisiae*), are increasingly recognized as contributors to gastric cancer development. These fungi can create a protumorigenic environment by interacting with the gastric microbiome, epithelial cells, and immune system.

*C. albicans*, a common fungal inhabiting the gastrointestinal tract, is frequently detected in gastric cancer tissues. It can metabolize dietary sugars to produce ethanol, a compound that damages gastric epithelial cells by disrupting cellular membranes and inducing oxidative stress.^83^ The inflammatory microenvironment created by *C. albicans* also facilitates the colonization of *H. pylori*.^186^ *H. pylori* exploits the epithelial disruption and altered immune landscape caused by *C. albicans* to facilitate its establishment within the gastric mucosa more effectively.^187^ In addition to *C. albicans*, *S. cerevisiae*, commonly known as baker’s yeast, is also implicated in gastric cancer. While typically considered a benign or even beneficial fungus, certain strains of *S. cerevisiae* can produce β-glucans, which are components of their cell walls that modulate immune responses. In the gastric TME, fungal β-glucans may activate immune checkpoints that suppress antitumor immunity.^188^ By interacting with pattern recognition receptors such as Dectin-1 on immune cells, β-glucans can induce an immunosuppressive state, allowing tumor cells to evade immune surveillance.^189^

Clinically, potential diagnostic and therapeutic implications can be determined on the basis of the presence of fungal dysbiosis in gastric cancer. The detection of fungal-specific markers, such as *C. albicans* adhesins or β-glucans, may facilitate early diagnosis or risk stratification. Therapeutically, antifungal treatments aimed at reducing the fungal load combined with *H. pylori*-targeted antibiotics have shown promise in preclinical models.^190^ Additionally, probiotics containing beneficial yeast strains, such as nonpathogenic *S. cerevisiae*, are being explored for their potential to restore microbial balance and mitigate fungal-mediated carcinogenesis.^191^

#### Hepatobiliary cancers

Fungi, although less studied than bacteria are, are increasingly recognized as contributors to hepatobiliary carcinogenesis. Fungal species such as *C. albicans* and *A. fumigatus* have been detected in the liver and biliary tract of patients with hepatocellular carcinoma and cholangiocarcinoma. These fungi can produce proinflammatory metabolites and activate immune pathways that disrupt normal tissue homeostasis, thereby exacerbating inflammation to create a microenvironment conducive to tumor growth.^192^

*C. albicans*, in particular, has been shown to enhance immune tolerance in the liver through the secretion of β-glucans, which interact with pattern recognition receptors on immune cells, suppressing the activation of antitumor immune responses.^193^ Fungal biofilms, which form in both the liver and biliary tract, can further protect pathogens from immune clearance to facilitate tumor development.^194^

#### Pancreatic cancer

Fungal dysbiosis in PDAC is characterized by the enrichment of *Malassezia* spp., a genus of fungi generally linked to skin and sebaceous gland disorders. *Malassezia* spp. can translocate from the gastrointestinal tract to the pancreas, where they colonize the TME.^195^ This colonization is facilitated by the breakdown of the intestinal barrier, which is often exacerbated by conditions such as chronic pancreatitis or systemic inflammation. Once established in the pancreas, *Malassezia* spp. can activate the complement system, particularly the C3 pathway, to support tumor growth. In mouse models, antifungal treatments targeting *Malassezia* spp. increase the tumor burden, underscoring the antioncogenic role of Malassezia spp. in pancreatic carcinogenesis.^195^ Moreover, *C. albicans* has been found to be involved in the progression of PDAC. *C. albicans* can produce acetaldehyde, a carcinogenic metabolite that induces DNA damage and promotes genomic instability in pancreatic epithelial cells. Additionally, fungal biofilms formed by *C. albicans* create a protective niche that shields both fungi and tumor cells from immune clearance and therapeutic agents, leading to tumor persistence and treatment resistance.^196^

The interplay between fungal and bacterial communities in the pancreas further complicates the understanding of pancreatic carcinogenesis. Fungal dysbiosis can exacerbate bacterial overgrowth and vice versa, creating a synergistic effect that amplifies inflammation and tumor-promoting signals. Fungi may alter the metabolic landscape of the TME to facilitate the proliferation of pathogenic bacteria that produce carcinogenic metabolites.^197^ Consequently, this mutualistic relationship between fungi and bacteria establishes a highly inflammatory and immune-suppressive microenvironment conducive to tumor growth.

#### Colorectal cancer

In colorectal cancer, the gut mycobiome is markedly imbalanced, i.e., an overabundance of pathogenic fungi such as *C. albicans* and *Malassezia* spp. Specifically, *C. albicans* can adhere to and invade colonic epithelial cells to trigger and amplify inflammation resulting from disruption of the intestinal barrier.^198^ The fungus secretes aspartyl proteases and phospholipases that degrade the epithelial layer, facilitating the translocation of microbial products into the underlying tissue and triggering immune responses. *C. albicans* can also produce ethanol and acetaldehyde during its metabolic processes, both of which are genotoxic and can induce DNA damage in colonic epithelial cells.^199^ Moreover, *Malassezia* spp., which are traditionally associated with skin conditions, have been increasingly detected in colorectal cancer tissues. *Malassezia* spp. may promote colorectal cancer progression by activating complement pathways such as C3 signaling.^197^

With respect to the above findings, colorectal cancer pathogenesis may be significantly affected by the interplay between fungi and bacteria in the gut microbiome. Fungal species such as *C. albicans* can synergize with pathogenic bacteria such as *F. nucleatum* to increase inflammation and disrupt the intestinal barrier. *C. albicans* biofilms provide a protective mechanism, allowing bacterial pathogens to evade immune clearance and persist in the gut. Eventually, the proposed mutualistic relationship exacerbates epithelial damage and promotes a protumorigenic microenvironment.^200^

#### Genitourinary cancers

The balanced vaginal mycobiome is a guardian for maintaining a healthy genital environment, but its disruption may imply an increased risk of cervical cancer. *C. albicans*, one of the most common fungal species in the vagina, has been implicated in cervical carcinogenesis, particularly in the context of coinfection with HPV. Chronic *C. albicans* infections may create a sustained inflammatory microenvironment to facilitate persistent HPV existence and integration into the host genome. This can be attributed to the ability of the fungus to produce proteolytic enzymes that degrade epithelial barriers and expose basal cells to HPV infection.^201^ *C. albicans* can also modulate immune responses by recruiting Tregs and suppressing cytotoxic T-cell activity, allowing HPV-infected cells to evade immune surveillance. Furthermore, the presence of fungal biofilms can exacerbate this process by creating a microenvironment conducive to viral replication and epithelial transformation.^202^

#### Melanoma and bone-related tumors

The skin, as a primary barrier organ, harbors a rich fungal community, with *Malassezia* spp. and *C. albicans* being the most prevalent.^203^ *Malassezia* spp., which are traditionally linked to dermatological conditions, have been detected in melanoma lesions, where they interact with keratinocytes and immune cells to promote a protumorigenic environment. *M. globosa* can activate complement pathways, particularly C3, within the melanoma microenvironment, promoting tumor cell proliferation and angiogenesis. In addition, fungal metabolites [e.g., lipases and fatty acids] can modulate lipid metabolism in melanoma cells to increase their ability to invade and metastasize.^204^

To date, few studies have focused on primary bone tumors such as osteosarcoma and chondrosarcoma in the context of the fungal microbiota. However, significant microbial influences have been revealed in research on metastatic bone diseases in cancers such as breast, prostate, and lung cancer. In metastatic bone tumors, fungal-derived β-glucans and mannans can interact with osteoblasts and osteoclasts to modulate bone resorption and deposition. These interactions may induce a “vicious cycle” of bone metastasis, where tumor cells promote osteoclast activity, leading to bone degradation and the release of growth factors that further fuel tumor progression.^205^

### Viral microbiota in cancer

Acting as critical components of the human microbiota, viruses play significant roles in carcinogenesis and progression. Certain oncogenic viruses, such as HPV and EBV, have been conclusively linked to specific cancers. Their oncogenic mechanisms may include integration into the host genome, disruption of cellular pathways, immune evasion, and induction of chronic inflammation.^206^

HPV is a double-stranded DNA virus and one of the most well-studied oncogenic viruses reported mostly in cervical cancer, as well as in oropharyngeal, anal, and vulvar cancers. High-risk HPV types (e.g., HPV-16 and HPV-18) account for the majority of HPV-related cancers. With respect to oncogenic mechanisms, HPV-induced carcinogenesis begins with the infection of epithelial cells, often at sites of microabrasions. Following infection, the viral DNA integrates into the host genome to disrupt normal cell function. E6 and E7 are two key viral oncoproteins involved in this process. The E6 protein binds to and promotes the degradation of the tumor suppressor protein p53, inhibiting apoptosis and enabling the accumulation of genetic mutations, and it can also activate telomerase, contributing to cellular immortality. The E7 protein inactivates the retinoblastoma protein, a key regulator of the cell cycle, thus driving uncontrolled cellular proliferation and disrupting cell cycle checkpoints. The interaction of these oncoproteins with the host cellular machinery leads to genomic instability, increased cell division, and evasion of immune surveillance, all of which are hallmarks of cancer.^207^ Moreover, HPV infection also creates a proinflammatory microenvironment, resulting in proinflammatory (e.g., IL-6 and IL-8)-driven chronic inflammation, thereby facilitating angiogenesis and tumor progression. In addition, this virus also suppresses immune responses by downregulating antigen presentation pathways to help infected cells evade immune detection.^208^ Prophylactic vaccines targeting high-risk HPV types, such as Gardasil and Cervarix, have proven effective in preventing HPV-associated cancers.^209^ However, further investigations are still needed to identify therapeutic approaches, including checkpoint inhibitors and adoptive T-cell therapies, that target established HPV-driven tumors.

EBV from the herpesvirus family is another well-known oncogenic virus implicated in various cancers, including nasopharyngeal carcinoma, gastric cancer, and certain lymphomas, such as Burkitt lymphoma and Hodgkin lymphoma.^210^ EBV establishes latent infection in B cells and epithelial cells, contributing to carcinogenesis through its ability to manipulate host cell signaling and evade the immune system.^210^ LMP1 acts as a constitutively active receptor that mimics CD40 signaling, leading to the activation of the NF-κB, Janus kinase/signal transducer and activator of transcription (JAK/STAT), and PI3K/AKT pathways. It may eventually promote cell proliferation, inhibit cell apoptosis, and increase the metastatic potential.^211^ Epstein‒Barr nuclear antigen 1 ensures viral genome maintenance in host cells and causes genomic instability by inducing DNA damage. EBV-encoded small RNAs and microRNAs (miRNAs) can modulate host immune responses, suppressing T-cell activation and facilitating immune evasion.^212^ EBV infection can also result in the recruitment of Tregs and MDSCs, thus creating an immunosuppressive TME that provides a niche, allowing EBV-infected cells to evade immune clearance and progress to malignancy.^213^ At present, no vaccines for EBV exist. Clinical trials are currently in progress to verify the effects of immune-based therapies such as EBV-specific T-cell therapy and ICIs targeting the PD-1/PD-L1 pathway.^214^

HBV and HCV, both hepatotropic viruses, are leading causes of hepatocellular carcinoma. Chronic infections with these viruses may cause and amplify persistent inflammation, fibrosis, and cirrhosis, all of which may promote the development of liver cancer.^215^ HBV encodes a viral oncoprotein, i.e., an X protein known as HBx, which may disrupt cellular processes by interacting with transcription factors, pathways, and tumor suppressors. Moreover, HBx can lead to the activation of pathways such as the Wnt/β-catenin and PI3K/AKT pathways to promote cell proliferation and survival. HBV integration into the host genome may induce genomic instability, insertional mutagenesis, and dysregulation of host genes involved in cell growth and apoptosis.^216^ Moreover, despite the absence of a direct oncogenic nature, HCV infection may play a tumor-promoting role by creating a chronic inflammatory environment in the liver. Persistent inflammation may further lead to hepatocarcinogenesis owing to the presence of oxidative stress and DNA damage.^215^ HCV proteins, such as the core protein and nonstructural protein 5 A, can increase cell survival and proliferation by modulating pathways such as the MAPK and JAK/STAT pathways.^217^ Antiviral therapies, such as nucleoside analogs for HBV and direct-acting antivirals for HCV, have been documented to significantly reduce the incidence of hepatocellular carcinoma in treated populations.^218^

### Other microbial components in cancer

Other microbial components (e.g., archaea and protozoa), in addition to bacteria, fungi, and viruses, play intriguing roles in carcinogenesis, progression, and therapeutic response. Emerging evidence suggests that these microorganisms, despite less data on their contributions to carcinogenesis than the major microbial groups do, may significantly influence the TME and host‒pathogen interactions in unique ways.

Archaea are a vast group of distinct groups of microorganisms associated frequently with extreme environments and are also present in the human microbiota, particularly in the gut.^219^ Methanogenic archaea, such as *Methanobrevibacter smithii* and *Methanosphaera stadtmanae*, are among the top species in the human body.^220^ These methanogens may influence host physiology by producing methane during anaerobic digestion of substrates such as hydrogen and carbon dioxide. Methane production is associated with alterations in gut motility and microbiota composition, which can create a dysbiotic environment that favors carcinogenesis, particularly in patients with colorectal cancer.^221^ Archaea may also play a tumor-promoting role through the modulation of inflammation and oxidative stress. Specifically, methanogens can interact with bacterial communities to promote the production of inflammatory mediators (e.g., IL-6 and TNF-α) to mediate the progression of carcinogenesis.^222^ *Entamoeba histolytica* (*E. histolytica*) is the causative agent of amoebiasis and is associated with the occurrence of colorectal cancer. Chronic colonization and tissue invasion by *E. histolytica* may induce persistent inflammation and oxidative stress, resulting in DNA damage and epithelial dysplasia.^223^ *Trichomonas vaginalis* (*T. vaginalis*) is a sexually transmitted protozoan that may be related to prostate and cervical cancers. Persistent infection with *T. vaginalis* may create a favorable environment for carcinogenesis owing to the stimulation of chronic inflammation and immune evasion. This protozoan produces adhesins and proteases that disrupt epithelial barriers and enhance cellular proliferation, potentially synergizing with HPV and other oncogenic viruses in cervical cancer.^224^ Another protozoan, *Toxoplasma gondii* (*T. gondii*), has been proposed as a potential contributor to carcinogenesis because of its ability to establish chronic infections. *T. gondii* may indirectly promote cellular proliferation and survival by modulating host cell pathways, such as those involving STAT3 and PI3K/AKT. In addition, its immunomodulatory effects, including the induction of Tregs, may suppress antitumor immune responses to increase tumor progression.^225^

Therapeutic strategies targeting archaea and protozoa are still in their infancy but hold promise. Existing cancer therapies can be complemented by probiotic formulations designed to balance archaeal populations or antiprotozoal treatments aimed at mitigating chronic infections.^226^ Further research into these less-studied microbial groups will further increase our understanding of their contributions to cancer biology and create a strong foundation for innovative microbiota-targeted interventions.

## Signaling pathways and regulatory mechanisms involved in microbiota-mediated cancer progression

**Fig. 3:**
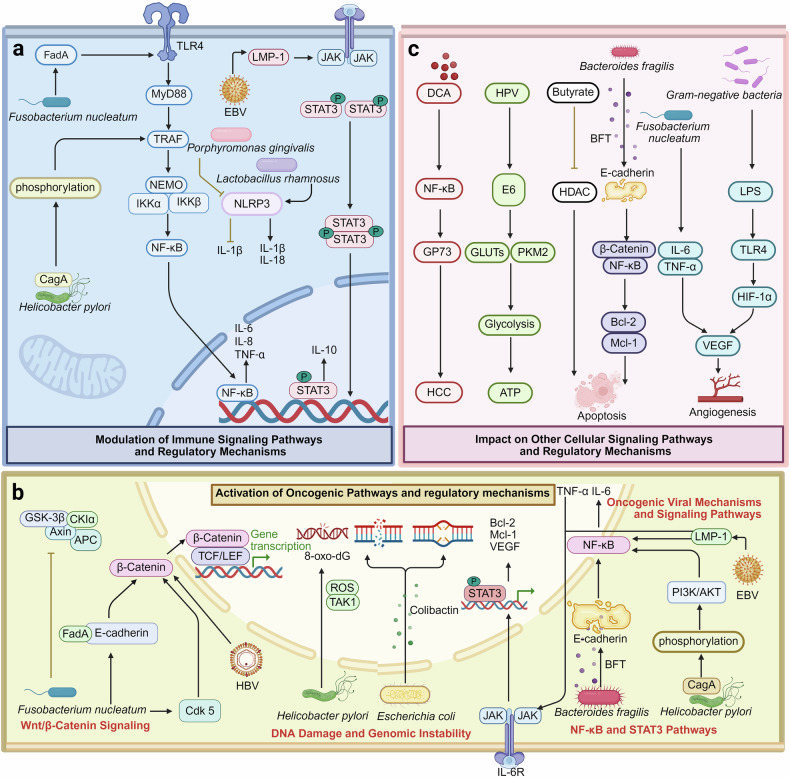
Microbial contributions to cancer progression via various pathways and mechanisms. **a** Specific microbial species and their metabolites can modulate immune responses through distinct mechanisms, impacting pathways such as the TLR, NF-κB, JAK/STAT, and inflammasome pathways. **b** Microbiota can drive tumorigenesis by inducing DNA damage and genomic instability. Oncogenic progression is further accelerated via persistent activation of the Wnt/β-catenin, NF-κB and STAT3 signaling cascades, as well as by infection with oncogenic viruses. **c** Specific microbes and their metabolites influence tumor progression by reprogramming metabolism, promoting angiogenesis, modulating apoptosis, and altering the epigenetic landscape. 8-oxo-dG 8-oxo-7,8-dihydroguanine, DCA deoxycholic acid, HCC hepatocellular carcinoma, VEGF vascular endothelial growth factor, BFT *Bacteroides fragilis* toxin, HDAC histone deacetylase. Created in BioRender. Wang, Y. (2025) https://BioRender.com/7bt0e3l

Critically, the interplay between the microbiota and cancer progression involves intricate and dynamic regulatory networks that influence critical processes such as oncogenesis, tumor immune evasion, and therapeutic resistance. These direct and indirect interactions create a dynamic feedback loop, with the microbiota influencing the tumor and, in turn, being shaped by the metabolic and immunological milieu of the TME.^227^ This bidirectional relationship highlights the importance of understanding microbiota‒host signaling networks, as they offer potential therapeutic targets for disrupting tumor-promoting pathways and enhancing anticancer responses (Fig. 3).

### Activation of oncogenic pathways and regulatory mechanisms

Microbial communities have evolved diverse strategies to interact with host cells, and these interactions, in the context of cancer, can activate oncogenic pathways to drive carcinogenesis and progression. The mechanisms involve direct molecular actions, such as the production of genotoxins, as well as indirect effects mediated by chronic inflammation, immune modulation, and metabolic reprogramming (Fig. 3b).

#### DNA damage and genomic instability

The microbiota can contribute to cancer through the induction of DNA damage and genomic instability, representing one hallmark mechanism.^207^ A notable example is the genotoxin colibactin, which is produced by *E. coli* strains harboring the polyketide synthase genomic island. By forming covalent DNA adducts, colibactin may cause double-strand breaks and interstrand crosslinks (Fig. 3b). This damage may trigger an error-prone repair process, increasing the mutation burden in host cells and driving colorectal carcinogenesis.^228^ Studies, both in vitro and in vivo, have demonstrated that mice colonized with polyketide synthase-positive *E. coli* exhibit increased tumor formation in the colon, reinforcing the causal role of these bacteria in colorectal cancer progression.^229^ In addition to colibactin, certain gut bacteria can also produce ROS and reactive nitrogen species, which can oxidatively damage DNA bases such as guanine, forming 8-oxo-dG lesions. The accumulation of such oxidative lesions, combined with deficiencies in DNA repair mechanisms such as mismatch repair, creates a promutagenic environment. For example, *H. pylori*, which is implicated in gastric cancer, can induce ROS through neutrophil recruitment and activation, linking chronic infection to increased mutation rates in gastric epithelial cells.^230^

#### Wnt/β-catenin signaling

*F. nucleatum* has been increasingly recognized for its role in colorectal cancer progression, particularly through the activation of the Wnt/β-catenin pathway.^231^ This bacterium expresses the adhesin FadA, which binds to E-cadherin on colonic epithelial cells, disrupting cell‒cell adhesion and initiating intracellular signaling cascades. This interaction may induce the activation and nuclear translocation of β-catenin, resulting in the transcription of oncogenes (e.g., c-MYC and cyclin D1) to stimulate cells to proliferate and survive.^232^ Accumulating evidence has elucidated the mechanisms by which *F. nucleatum* influences β-catenin signaling. Li et al. demonstrated that *F. nucleatum* could enhance colorectal cancer progression through cyclin-dependent kinase (CDK) 5-mediated activation of Wnt/β-catenin signaling. In their investigation, *F. nucleatum* infection increased the expression of β-catenin, *c-MYC*, and *cyclin D1* in colorectal cancer cell lines, and Cdk5 knockdown significantly abrogated these effects, highlighting the role of Cdk5 in this process. Additionally, *F. nucleatum* was reported to promote colorectal cancer by inducing the Wnt/β-catenin modulator Annexin A1.^231^ Moreover, *F. nucleatum* stimulates the growth of colorectal cancer cells by upregulating Annexin A1, which in turn ultimately activates Wnt/β-catenin signaling to promote tumor development.^233^ Clinical studies have corroborated these findings, showing that colorectal cancer patients with a high *F. nucleatum* load exhibit increased β-catenin activity and worse prognoses. The presence of *F. nucleatum* in tumor tissues has been associated with increased tumor stages, increased proliferation indices, and reduced overall survival, underscoring the clinical relevance of this bacterium in colorectal cancer progression.^234^ In summary, *F. nucleatum* contributes to colorectal carcinogenesis through multiple mechanisms involving the activation of the Wnt/β-catenin pathway. Its interactions with host cell receptors and modulation of intracellular signaling cascades underscore the complex role of the microbiota in carcinogenesis and highlight its potential therapeutic targets for colorectal cancer treatment.

#### NF-κB and STAT3 pathways

Chronic inflammation is a well-established driver of cancer, with the microbiota playing a pivotal role in sustaining inflammatory signaling through the NF-κB and STAT3 pathways.^235^ *H. pylori*, the first bacterium classified as a human carcinogen, exemplifies this mechanism. Upon infection, *H. pylori* utilizes a type IV secretion system to inject the virulence factor CagA into gastric epithelial cells.^236^ Once inside the host cell, CagA undergoes tyrosine phosphorylation and interacts with SHP-2 phosphatase, further activating downstream NF-κB signaling.^237^ Upon activation, it may promote the production of proinflammatory cytokines such as TNF-α and IL-6, contributing to gastric mucosal inflammation, epithelial proliferation, and eventual malignant transformation.^237^ The activation of NF-κB has been confirmed to precede STAT3 activation in response to *H. pylori* infection, suggesting that sequential activation of NF-κB may be necessary for subsequent STAT3 activation. Persistent STAT3 activation can induce antiapoptotic proteins (e.g., Bcl-2 and Mcl-1) and angiogenic factors [e.g., vascular endothelial growth factor (VEGF)], forming a microenvironment conducive to tumor growth.^238^ Similar mechanisms have been observed with gut-residing *B. fragilis*, particularly enterotoxigenic strains that produce a metalloprotease toxin known as BFT. BFT can disrupt E-cadherin on colonic epithelial cells, which may subsequently activate the β-catenin and NF-κB pathways. This disruption results in the secretion of proinflammatory cytokines (e.g., IL-8), promoting a protumorigenic inflammatory environment.^239^ In murine models, enterotoxigenic strain colonization can induce colitis and promote colorectal carcinogenesis through the IL-17R, NF-κB, and STAT3 pathways. Collectively, these findings underscore the intricate relationship of microbial infections and chronic inflammation with carcinogenesis. The activation of the NF-κB and STAT3 pathways by bacterial pathogens such as *H. pylori* and *B. fragilis* highlights potential therapeutic targets for interrupting the inflammation‒cancer axis.

#### Oncogenic viral mechanisms and signaling pathways

Oncogenic viruses play multifaceted roles in cancer by hijacking the host cellular machinery, disrupting critical regulatory pathways, and fostering a tumor-supportive microenvironment. Their well-documented ability is to integrate into the host genome and produce viral oncoproteins. Moreover, these viruses (e.g., HPV, EBV, HBV, and HCV) can exploit and modulate a broad range of host signaling cascades to drive oncogenesis.^211,240^

Specifically, HPV exploits host pathways with its viral oncoproteins E6 and E7, but their effects extend beyond the degradation of the tumor suppressors p53 and the retinoblastoma protein. For example, E6 activates PI3K/AKT signaling by linking to the p85 regulatory subunit of PI3K to promote cell survival and growth. E7 dysregulates CDKs, driving the phosphorylation of the retinoblastoma protein and forcing cells into uncontrolled S-phase entry.^241^ Together, these mechanisms create an environment primed for transformation by enhancing cellular proliferation while evading apoptosis. Similarly, EBV utilizes its LMP1 protein to establish a chronic protumorigenic state. LMP1 mimics CD40 receptor signaling to activate the canonical and noncanonical NF-κB pathways. It may further upregulate prosurvival genes such as *Bcl-2* and antiapoptotic factors, along with cytokines that recruit MDSCs.^242^ LMP1 may also activate PI3K/AKT and JAK/STAT signaling, promoting metabolic reprogramming, angiogenesis, and immune evasion.^243^ Both HPV and EBV can induce epigenetic modifications in host cells. HPV infection induces global DNA hypomethylation while causing hypermethylation of tumor suppressor genes such as *p16INK4A*.^244^ EBV’s Epstein‒Barr nuclear antigen 1 and viral miRNAs target DNA methyltransferases, enhancing the silencing of tumor suppressors and promoting immune escape.^245^ In addition, these viruses also amplify inflammatory feedback loops, with HPV enhancing IL-6/STAT3 signaling and EBV upregulating IL-10, creating a tumor-promoting immunosuppressive milieu.^246^

Unlike HPV and EBV, hepatotropic viruses (e.g., HBV and HCV) exert oncogenic effects depending primarily on chronic inflammation and oxidative stress.^247^ HBV encodes HBx, a multifunctional protein that activates β-catenin and STAT3 signaling.^248^ HBx also disrupts mitochondrial function, generating ROS and inducing DNA damage. Moreover, the ability of HBV to integrate into the host genome may exacerbate genomic instability and disrupt tumor suppressor loci.^249^ HCV, without direct integration into host DNA, can induce persistent endoplasmic reticulum stress and oxidative damage on the basis of its core protein and nonstructural protein 5 A. These proteins can modulate MAPK and JNK signaling, promoting hepatocyte survival and proliferation in a proinflammatory microenvironment.^250^ HCV infection also skews immune signaling by downregulating interferon (IFN) responses to trigger immune evasion.^251^

### Modulation of immune signaling pathways and regulatory mechanisms

The microbiota can interact with immune pathways to function significantly in shaping the TME, influencing cancer initiation, progression, and therapeutic responses. Specific microbial species and their metabolites can modulate immune responses through distinct mechanisms, impacting pathways such as the TLR, NF-κB, JAK/STAT, and inflammasome pathways. These interactions may further alter cytokine production, immune cell activation, and the balance between protumorigenic and antitumorigenic immune responses^252^ (Fig. 3a).

#### Toll-like receptor pathways

Certain microbiota may activate or suppress immune signaling through TLRs, a family of pattern recognition receptors critical for detecting microbial-associated molecular patterns. For example, *F. nucleatum* interacts with TLR4 via its FadA adhesin. This interaction can further activate downstream MyD88 and NF-κB pathways to stimulate the secretion of proinflammatory cytokines such as IL-6 and TNF-α. Consequently, a chronic inflammatory environment may be created to facilitate tumor cell proliferation and immune evasion.^253^ Conversely, beneficial commensals such as *A. muciniphila* can stimulate TLR2 to trigger the release of anti-inflammatory cytokines such as IL-10. This process can activate T cells and restore the balance between immune suppression and activation, thereby enhancing the efficacy of immune checkpoint blockade (ICB).^254^

#### NF-κB pathway

The NF-κB pathway is a master regulator of inflammation and immunity and is often hijacked by both the microbiota and tumor cells to create a protumorigenic environment. For example, *H. pylori* exploits this pathway through the participation of its virulence factor CagA. Upon delivery into gastric epithelial cells via a type IV secretion system, CagA can activate NF-κB by interacting with SHP-2 phosphatase and TRAF6. This activation may stimulate the release of IL-8 and other proinflammatory cytokines, which recruit neutrophils and monocytes, driving chronic inflammation and DNA damage that facilitate tumor progression.^255^ Similarly, enterotoxigenic strains may produce BFT to cleave E-cadherin in colonic epithelial cells. This disruption can further trigger β-catenin and NF-κB activation, resulting in increased secretion of IL-17 and recruitment of Th17 cells. Proinflammatory IL-17 can further amplify inflammatory signaling to promote colorectal carcinogenesis.^239^

#### JAK/STAT signaling

The JAK/STAT pathway is another critical immune signaling cascade modulated by the microbiota that affects tumor immunity. *E. coli* strains carrying the polyketide synthase pathogenicity island can produce colibactin, a genotoxin that causes DNA damage and activates STAT3 signaling in epithelial cells.^256^ Persistent STAT3 activation can create a tumor-supportive microenvironment by increasing the expression of antiapoptotic proteins (e.g., Bcl-2 and survivin).^257^ In the context of the viral microbiota, EBV is known to modulate STAT3 through its LMP1, which mimics CD40 receptor signaling. This activation may induce the secretion of IL-10, a cytokine that suppresses cytotoxic T lymphocytes and dendritic cells, further contributing to immune evasion and tumor progression.^258^

#### Inflammasome pathways

Inflammasomes are a group of cytosolic complexes that detect microbial products and cellular stress and are tightly regulated by the microbiota to influence tumor immunity.^52^ *P. gingivalis* has been shown to suppress the NOD-like receptor family pyrin domain containing 3 (NLRP3) inflammasome in macrophages. This suppression can reduce the secretion of IL-1β, a cytokine critical for recruiting and activating neutrophils to clear tumor cells. Through the inactivation of inflammasomes, *P. gingivalis* can create a permissive environment for tumor growth.^259^ Conversely, certain commensals, such as *Lactobacillus rhamnosus* (*L. rhamnosus*), may enhance NOD-like receptor family pyrin domain containing 3 inflammasome activation, promoting a robust immune response against tumor cells by increasing IL-1β and IL-18 levels.^260^ Collectively, all these related studies have documented the dual role of inflammasomes in cancer biology, depending on the microbial context.

#### Immunosuppressive pathways

The microbiota may play a regulatory role in the recruitment and function of Tregs and MDSCs, which are key immunosuppressive players in the TME. For example, microbial metabolites, including SCFAs and bile acids, can regulate Treg differentiation and TGF-β secretion.^261^ Similarly, *C. difficile* infection has been associated with the expansion of MDSCs, which contribute to immune suppression and can enhance tumor progression. MDSCs can inhibit T-cell activation through the activity of enzymes such as arginase-1 and inducible nitric oxide synthase (iNOS). Furthermore, arginase-1 depletes L-arginine, an amino acid essential for T-cell function, leading to reduced T-cell proliferation and activation. iNOS produces nitric oxide to facilitate the suppression of T-cell responses and promote cell apoptosis. Together, these mechanisms contribute to the formation of an immunosuppressive environment associated with MDSC activity.^262^

### Impact on other cellular signaling pathways and regulatory mechanisms

The microbiota is significantly involved in the process of cancer progression by modulating cellular pathways, in addition to direct oncogenic and immune-related mechanisms. These effects include metabolic reprogramming, angiogenesis, apoptosis modulation, and epigenetic alterations, depending specifically on the specific microbial species and their metabolites. These pathways create a permissive environment for tumor growth, metastasis, and resistance to therapy^29^ (Fig. 3c).

#### Metabolic reprogramming and cross-talk

The central roles of microbial metabolites in the metabolic reprogramming of cancer cells, which can mediate subsequent carcinogenesis and progression, have been underscored by emerging evidence. Secondary bile acids, such as deoxycholic acid, and SCFAs, such as butyrate, produced by the gut microbiota have been implicated in these processes.^263,264^

Deoxycholic acid, a secondary bile acid resulting from microbial metabolism, has been shown to be related to the development of hepatocellular carcinoma. Elevated levels of deoxycholic acid can induce oxidative stress and DNA damage in hepatocytes to promote hepatic carcinogenesis.^265^ Deoxycholic acid can also upregulate Golgi protein 73 expression by activating NF-κB, suggesting a mechanistic link between deoxycholic acid-induced oxidative stress and hepatocellular carcinoma progression.^266^ Deoxycholic acid can also induce hepatocyte apoptosis through mechanisms involving sodium taurocholate cotransporting polypeptide. The expression levels of this protein in hepatocellular carcinoma cells can influence the response to deoxycholic acid, indicating that it may play a role in deoxycholic acid-induced alterations in hepatocellular carcinoma cell growth.^265^ Furthermore, butyrate is a prominent SCFA produced by the fermentation of dietary fibers by the gut microbiota and plays a dual role in colorectal cancer. In normal colonic epithelial cells, butyrate serves as a primary energy source to maintain cellular health and intestinal homeostasis.^267^ Conversely, in colorectal cancer cells, butyrate accumulates due to the Warburg effect, where cancer cells preferentially utilize glycolysis over oxidative phosphorylation, leading to its role as a histone deacetylase inhibitor. This inhibition results in hyperacetylation of histones, altering gene expression levels to promote cell cycle arrest, differentiation, and apoptosis in cancerous cells.^268^ This phenomenon, known as the “butyrate paradox”, highlights the various effects of butyrate in a context-dependent manner.^269^ Furthermore, butyrate can inhibit the activation of the IFN-gamma (IFN-γ)/STAT1 pathway to suppress colonic inflammation. The mechanism may involve the inhibition of histone deacetylase 1 activity bound to the Fas gene promoter in T cells, leading to the upregulation of the Fas receptor and promoting the apoptosis of T cells in colonic tissue, thereby relieving inflammation.^270^ Interestingly, butyrate has been recently shown to bind directly to the TLR5 receptor on CD8^+^ T cells, inducing its activity through the activation of NF-κB signaling, which improved anti-programmed cell death protein 1 (anti-PD-1) efficacy. However, in lung cancer, it can induce M2 macrophages that promote tumor metastasis.^271^

Viruses universally reprogram host metabolism to meet the biosynthetic and energy demands of proliferating cancer cells. HPV-driven cancers exhibit increased glucose uptake and glycolysis through E6-mediated upregulation of glucose transporters (GLUTs) and pyruvate kinase M2 (PKM2).^272^ Similarly, EBV and HBV manipulate lipid metabolism; EBV enhances fatty acid synthesis through sterol regulatory element-binding protein activation, whereas HBV promotes cholesterol biosynthesis via the mevalonate pathway.^273^

In summary, microbial metabolites (e.g., deoxycholic acid and butyrate) play complicated roles in cancer metabolism and progression. Their effects are context-dependent, influencing various pathways, inducing oxidative stress, modulating epigenetic landscapes, and affecting immune responses. Importantly, these intricate interactions may provide reference data for the development of therapeutic interventions that target metabolic reprogramming in cancer.

#### Angiogenesis

Angiogenesis is defined as the process of new blood vessel formation, which is a critical step in tumor growth and metastasis, during which certain microbial populations play certain roles. Lipopolysaccharides from gram-negative bacteria can activate TLRs (e.g., TLR4) on endothelial cells, stimulating the hypoxia-inducible factor-1 alpha (HIF-1α) pathway. HIF-1α promotes the expression of VEGF, a key proangiogenic factor, facilitating the formation of blood vessels to supply tumors with nutrients and oxygen.^274^ For example, *H. hepaticus*, which is linked to liver tumors in mouse models, can promote angiogenesis through the upregulation of VEGF in the liver microenvironment.^275^ Similarly, *F. nucleatum* can induce angiogenesis indirectly by increasing the levels of inflammatory cytokines (e.g., IL-6 and TNF-α), further increasing VEGF production and endothelial cell proliferation.

#### Modulation of apoptosis and cell survival

The microbiota can alter apoptotic pathways, tipping the balance between cell death and survival in favor of tumor growth. The gut bacterium *B. fragilis*, particularly its enterotoxigenic strains, produces BFT, which disrupts E-cadherin signaling. This disruption can activate host survival pathways through β-catenin and NF-κB, leading to the upregulation of antiapoptotic proteins such as Bcl-2 and Mcl-1.^276^ In contrast, some commensal bacteria, such as *L. rhamnosus*, can promote cancer cell apoptosis. These beneficial microbes may stimulate the production of intratumoral ROS, activating intrinsic apoptotic pathways and reducing tumor cell survival.^260^ Therefore, the findings highlight the dual role of the microbiota in either promoting or inhibiting apoptosis, which depends on the microbial context and the host environment.

#### Epigenetic modifications

The microbiota can also alter the epigenetic landscape of cancer cells, altering DNA methylation and histone modification patterns that regulate related gene expression levels. For example, *F. nucleatum* can promote global DNA hypomethylation while inducing hypermethylation of specific tumor suppressor genes, such as MLH1, via microbial metabolites, resulting in genomic instability and immune evasion.^277^ Similarly, butyrate and other SCFAs may have an impact on histone acetylation and DNA methylation. Butyrate acts as a histone deacetylase inhibitor, reversing aberrant histone deacetylation and restoring the expression of genes critical for tumor suppression. However, its effects can vary depending on the metabolic status of the tumor and the composition of the surrounding microbiota.^268^ In gastric cancer, *H. pylori* infection is related to the hypermethylation of CpG islands in tumor suppressor genes such as *E-cadherin* and *RUNX3*, which is mediated by increased activity of DNA methyltransferases.^278^

The microbiota impacts a wide array of cellular pathways beyond immune and direct oncogenic effects, creating an environment conducive to tumor initiation, growth, and metastasis. In this context, the microbiota may serve as a critical modulator of cancer progression through reprogramming host metabolism, promoting angiogenesis, modulating apoptosis, and altering epigenetic landscapes. A comprehensive explanation of these mechanisms may offer novel insights into microbiota-targeted therapeutic strategies, such as metabolic inhibitors, antiangiogenic agents, and epigenetic therapies. These findings underscore the importance of integrating microbiota research into precision oncology frameworks.

## Pro- and antitumor functions of the microbiota

Given their multifaceted roles in cancer biology, the microbiota can perform both pro- and antitumor functions, depending on the specific microbial species, host context, environmental factors, etc.^279^ On the one hand, certain microbial communities and their metabolites may create favorable conditions for carcinogenesis by driving chronic inflammation, suppressing antitumor immune responses, inducing genomic instability, and facilitating processes such as angiogenesis and metastasis. On the other hand, from the perspective of tumor suppression, distinct microbial populations or metabolites may exhibit protective effects by enhancing anticancer immunity, producing metabolites that inhibit tumor growth, or interfering with oncogenic pathways.^280^

**Fig. 4:**
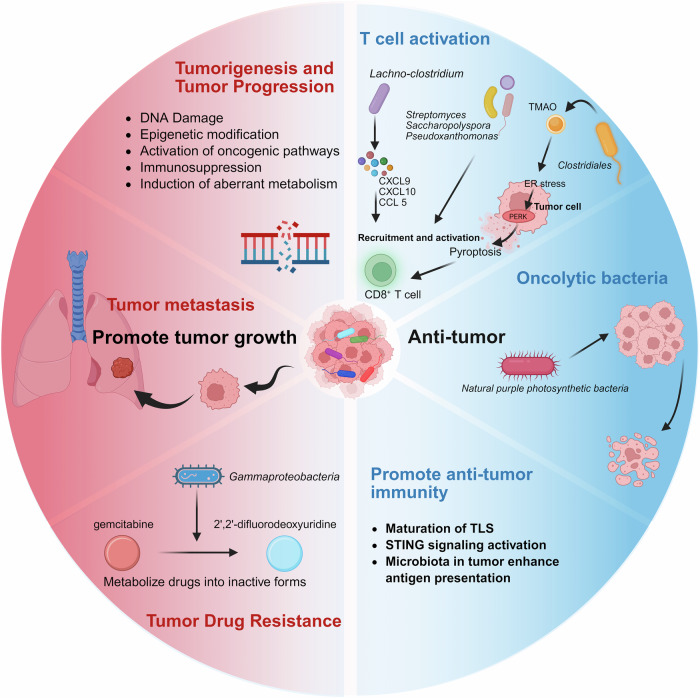
Pro- and antitumor functions of the microbiota. The cancer microbiota possesses both tumor-promoting and tumor-suppressing capabilities. These microbes can drive tumor progression or elimination through diverse mechanisms, including disrupting genes, modulating epigenetic modifications, and secreting bioactive products. ER endoplasmic reticulum, TMAO trimethylamine N-oxide, TLS tertiary lymphoid structure. Created in BioRender. Wang, Y. (2025) https://BioRender.com/o1vy9hz

The duality of these effects mirrors the complex interplay between the microbiota and host signaling networks, including immune modulation, metabolic reprogramming, and epigenetic regulation^280^ (Fig. 4). To derive the greatest value of their therapeutic benefits, understanding the precise mechanisms underlying these pro- and antitumor functions is essential for identifying microbial targets and pathways. On the basis of mechanistic exploration, researchers can develop strategies to mitigate the harmful effects of tumor-promoting microbes while amplifying the beneficial, antitumor properties of the commensal microbiota, thus providing valuable data for the formulation of novel approaches for cancer prevention and treatment. As described above, the microbiota can also inhibit cancer progression in certain contexts, in addition to promoting tumor occurrence and development through various mechanisms. For example, antitumor properties have been explored in Cladosporium species, a genus of endophytic fungi. Notably, secondary metabolites such as fusarubin and anhydrofusarubin, which are isolated from Cladosporium species, exhibited significant cytotoxic effects on various cancer cell lines. Fusarubin and anhydrofusarubin were found to inhibit proliferation and induce apoptosis in hematological cancer cell lines, including acute myeloid leukemia (AML) OCI-AML3, HL-60, U937, and Jurkat cells. The relevant mechanism involves the upregulation of p21 expression in a p53-dependent manner, leading to cell cycle arrest and the activation of apoptotic pathways.^281^ Another study categorized melanoma patients into low and high *Lachnoclostridium* load groups on the basis of the median abundance of this microbiota within their tumors. Further survival analysis revealed that patients in the high *Lachnoclostridium* load group exhibited significantly improved overall survival rates compared with those in the low *Lachnoclostridium* load group.

The microbial communities within tumors may elicit antitumor immune responses by activating immune cells. For example, the presence of certain microbiota might predict better prognoses in PDAC patients. Patients with better prognoses were found to have characteristic microbes (e.g., *Pseudoxanthomonas*, *Streptomyces*, *Saccharopolyspora* and *Bacillus*) within their tumors. These microbes are related to the activation of immune responses and the infiltration of T cells within the tumor. Therefore, some microbiota may accumulate and activate CD8^+^ T cells in tumor tissues, thereby enhancing the ability of the immune system to kill tumor cells.^282,283^

Trimethylamine N-oxide, which is secreted by *Clostridium* species, can activate protein kinase RNA-like endoplasmic reticulum kinase-mediated endoplasmic reticulum stress, increasing antitumor immune responses and improving the efficacy of immunotherapy for triple-negative breast cancer.^284^ Furthermore, *Lachnoclostridium* affects CD8^+^ T-cell infiltration by regulating the levels of the chemokines CXCL9, CXCL10, and CCL5, thereby influencing survival rates in patients with cutaneous melanoma. Moreover, the oncolytic microbiota, which is related to natural purple photosynthetic bacteria isolated from solid tumors, has demonstrated biocompatibility and potent immunogenic anticancer effects.^285^ These microbes might preferentially grow and multiply in the targeted tumor environment, promoting immune cell infiltration and eliciting strong anticancer responses in various syngeneic mouse models, including those of colorectal cancer, metastatic lung cancer, and extensively drug-resistant breast cancer. Mice treated with these bacteria exhibited superior anticancer responses and effective immune memory, significantly increasing survival rates.^285^ Consequently, these functional microbes demonstrate high tumor selectivity, environmental sustainability, and cost effectiveness, sparking researchers’ interest in their potential as natural active anticancer agents for innovative treatments.

For enhanced antitumor immune responses, relevant mechanisms include the activation of immune cells such as T cells, stimulation of the stimulator of interferon genes (STING) pathway, maturation of tertiary lymphoid structures, and enhancement of antigen presentation by tumor-intrinsic microbiota. Tumor-associated *Bifidobacteria* can activate the STING pathway, increase the cross-presentation ability of dendritic cells, and eventually enhance the efficacy of anti-CD47 antibody therapy.^286^ Hepatic *Helicobacter* species stimulate antitumor follicular helper T cells, B cells, and natural killer cell responses while promoting the maturation of tertiary lymphoid structures around the tumor.^287^ Tumor cells can present intracellular bacteria-derived peptides to activate specific T-cell responses. Additionally, orally administered *Lactobacillus reuteri* (*L. reuteri*) can migrate to melanoma tumor clusters, release indole-3 aldehyde (I3A), and enhance the therapeutic efficacy of ICIs.^161^

## Relationship between the microbiota and metabolism

**Fig. 5:**
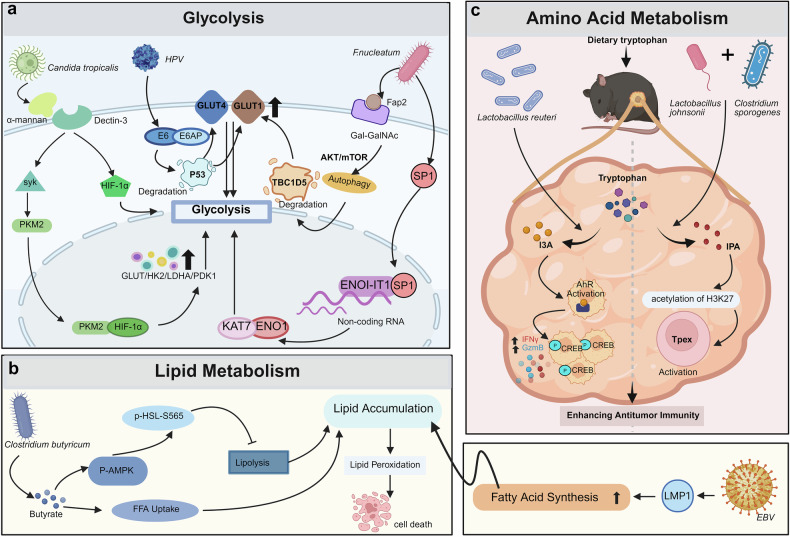
Intratumoral bacteria influence various metabolic processes within the body. **a** *F. nucleatum* can activate the transcription of ENO1-IT1 by increasing the binding efficiency of the transcription factor SP1 and promote KAT7 binding to the promoter region of the ENO1 gene to activate glycolysis. In OSCC, *F. nucleatum* binds to N-acetylgalactosamine (GalNAc) on the cell surface, inducing autophagy, degrading TBC1D5, upregulating GLUT1, and promoting glucose metabolism to lactate, thereby driving the formation of M2-like TAMs. The HPV virus promotes the degradation of p53, thereby increasing the expression and accumulation of GLUT4 and other proteins on the cell membrane. In fungi, *Candida albicans* can bind to Dectin-3 on the cell surface, activating HIF-1α-dependent glycolysis or increasing the production of glycolytic enzymes. **b** Lactobacillus reuteri can colonize melanoma, converting tryptophan to indole-3-aldehyde (I3A) to activate CD8 + T cells via AhR signaling, enhancing the ICI effects. Lactobacillus johnsonii and Clostridium sporogenes convert tryptophan to indole-3-propionic acid (IPA), increasing H3K27 acetylation in the Tcf7 gene superenhancer region, promoting precursor exhausted T-cell differentiation and enhancing the efficacy of ICB therapy in melanoma and breast and colorectal cancers. **c** Butyrate increases the absorption of free fatty acids by upregulating free fatty acid transport proteins or by activating the AMPK-phospho-HSL (S565) axis, thereby inhibiting lipolysis. Additionally, Epstein–Barr virus (EBV) can promote fatty acid synthesis. GalNAc N-acetylgalactosamine, OSCC oral squamous cell carcinoma, ICI immune checkpoint inhibitor, IPA indole-3-propionic acid, Tpex precursor exhausted T cells, ICB immune checkpoint blockade, FFA free fatty acids, EBV Epstein–Barr virus. Created in BioRender. Wang, Y. (2025) https://BioRender.com/6ca0ke1

Generally, cancer cells undergo extensive metabolic reprogramming to accommodate the demands of rapid proliferation, survival in hostile microenvironments, and metastasis. This metabolic shift, a defining hallmark of cancer, involves significant alterations across multiple metabolic pathways. Moreover, the complex interplay between tumors and their associated microbiota reveals a diverse spectrum of mutual influences. A comprehensive investigation of intricate metabolic interactions may reveal the complexities underpinning the tumor-associated microbiome (Fig. 5).

Notably, according to a metabolomic analysis of intrahepatic cholangiocarcinoma in murine models, marked variations were found in metabolite profiles between tumors in the experimental and control cohorts. These findings suggest that *Fusobacterium* may hinder tumor progression through the modulation of amino acid synthesis and metabolic pathways. In contrast, a recent study (2024) demonstrated that colibactin-producing *E. coli* bacteria significantly diversify lipid metabolism in tumors, thereby promoting both the progression of colon cancer and the development of resistance to chemotherapy.^288^

In HPV-associated cancers, the viral proteins E5 and E2, alongside E6 and E7, may induce the Warburg effect and increase resistance to radiotherapy and chemotherapy. This can be explained by the increase in glycolytic enzyme activity as well as the suppression of the Krebs cycle and the respiratory chain. These metabolic alterations create a favorable environment for rapid ATP production, thereby satisfying the heightened energy demands of proliferating cancer cells and ultimately driving tumor progression.^289^ Similarly, Byrd et al. reported that the microbiome associated with tissue affected by benign breast disease was associated with increased metabolism of cysteine and methionine, as well as increased glycosyltransferase activity and fatty acid biosynthesis. In contrast, within breast cancer tissue, the microbiome (e.g., *Fusobacterium*, *Atopobium*, *Hydrogenophaga*, *Gluconacetobacter*, and *Lactobacillus*) was found to suppress inositol phosphate metabolism, highlighting the elicitation of distinct metabolic alterations depending on different microbial species.^290^

### Glycolysis

The Warburg effect, proposed by Otto Warburg for the first time in the 1920s, is the most prominent metabolic alteration in cancer cells.^37^ This metabolic process, also called aerobic glycolysis, is defined by the conversion of glucose into lactate via glycolysis, even in the presence of an adequate oxygen supply.^291^ In this process, a unique feature of cancer cells is the conversion of glucose into lactate via aerobic glycolysis, facilitating the rapid production of ATP and the metabolic intermediates necessary for biosynthetic processes. This metabolic adaptation provides cancer cells with a distinct growth advantage. An increasing body of recent research has demonstrated the pivotal roles of tumor-associated microbes in promoting glycolysis through diverse mechanisms.

Recently, several researchers have underscored the ability of certain bacteria, including *F. nucleatum*, to stimulate glycolysis within the TME. For example, in the progression of colorectal cancer, metabolic reprogramming was found to supply energy to tumor cells.^292^ *F. nucleatum* specifically increased the expression of the long noncoding RNA *ENO1-IT1* by promoting the interaction between the transcription factor SP1 and the *ENO1-IT1* promoter region. This mechanism regulates histone modifications in genes such as *ENO1*, driving metabolic reprogramming in colorectal cancer cells, activating glycolysis, and facilitating oncogenic processes. Moreover, elevated levels of *ENO1-IT1* serve as a scaffold for the histone acetyltransferase KAT7, thereby modulating histone modifications in target genes, including *ENO1*, and influencing the biological functions of colorectal cancer cells. Moreover, Sun et al. demonstrated that *F. nucleatum* accelerated OSCC development by activating the GalNAc-autophagy-TBC1D5 pathway. This activation further upregulated GLUT1 expression and resulted in lactate accumulation, thereby driving the progression of OSCC.^90^

In accordance with prior studies, gut bacteria, particularly *Salmonella enterica* serovar Typhimurium, can regulate glycolysis levels in macrophages, potentially modulating their function and advancing the development of various gastrointestinal diseases.^293^ Recently, Tingting Wang et al. emphasized deciphering the role of symbiotic fungi in cancer metabolism. Through experimental studies in mice deficient in the c-type lectin receptor Dectin-3, macrophages infected with *C. albicans* were shown to increase glycolysis via a HIF-1-dependent pathway and secrete IL-7. This secretion further stimulates innate lymphoid cells to produce IL-22, thereby driving colorectal cancer progression.^294^

*Candida tropicalis* (*C. tropicalis*) can induce the upregulation of glycolysis in MDSCs, thereby supporting their survival and immunosuppressive functions. Metabolomic analyses further revealed that *C. tropicalis* can significantly increase glycolysis in MDSCs. In 2022, Tingting Wang et al. delved into the underlying mechanisms and innovatively demonstrated that *C. tropicalis* could strengthen the Syk-PKM2 interaction in MDSCs. They discovered that PKM2, which functions as a coactivator of HIF-1α, facilitated the expression of HIF-1α-dependent glycolytic enzymes, which in turn amplified aerobic glycolysis, the immunosuppressive activity of MDSCs, and the progression of colorectal tumors.^295^ Their subsequent research revealed that *C. tropicalis* might activate the NOD-like receptor family pyrin domain containing 3 inflammasome via the Dectin-3-glycolysis pathway, leading to increased IL-1β production by MDSCs.^296^

Research on virus-related mechanisms revealed that, in lung cancer patients, EBV infection of lung epithelial cells increased glucose uptake and upregulated glycolysis by increasing the expression of GLUTs and glycolytic enzymes via the viral protein LMP1. This interaction between EBV and cellular metabolism creates a microenvironment conducive to tumor occurrence and progression.^76^ Furthermore, Delgado et al. discovered that the use of glycolysis inhibitors, such as oxalate and 2-deoxyglucose, resulted in the selective induction of KSHV-infected endothelial cell apoptosis while sparing mock-infected cells. Therefore, latent viral infection and survival may be sustained in KSHV-transformed endothelial cells via glycolysis.^297^ In HPV16-associated cancers, the E5 protein indirectly modulates the Warburg effect via the epidermal growth factor-EGFR (EGF-EGFR) axis, thereby augmenting glycolytic metabolism in oral cancer cells.^298^ In addition, HPV E6 oncoprotein-mediated degradation of p53 results in the overexpression of GLUT1, leading to increased glucose uptake in cervical cancer cells.^289,299^

### Lipid metabolism

Lipid metabolism is a prerequisite for tumor cells to meet their energy demands and synthesize the lipids required for constructing cell membranes. Microorganisms can modulate fatty acid metabolism to regulate fatty acid synthesis, oxidation, and storage within tumor cells.^300^ Key enzymes involved in lipid biosynthesis, including fatty acid synthase, acetyl-CoA carboxylase, and ATP citrate lyase, have been confirmed to be involved in tumor development and progression.^301^

According to recent researches, the presence of butyrate-producing bacteria and their metabolic byproduct, butyrate, increases intracellular oxidative stress in PDAC cells. Butyrate has been demonstrated to impede tumor cell proliferation and modulate T-cell activation.^302^ They further reported that butyrate could mediate lipid metabolism by enhancing the uptake of free fatty acids while inhibiting lipid breakdown within lipid droplets via hormone-sensitive lipase (HSL). Moreover, butyrate results in elevated oxidative stress and subsequent lipid peroxidation owing to the disruption of mitochondrial energy metabolism.^303^ Therefore, butyrate may promote intracellular lipid accumulation through two key mechanisms, i.e., the upregulation of free fatty acid transport proteins to increase free fatty acid uptake and the suppression of lipolysis via the activation of the AMPK‒phospho-HSL axis.^303^ Furthermore, colibactin-producing *E. coli* have been identified within the TME, particularly in right-sided colon cancers, where they reduce tumor immunogenicity by establishing a glycerophospholipid-rich milieu. The genotoxin colibactin, produced by Colibactin-producing *E. coli*, may induce DNA double-strand breaks and foster genomic instability. This pathogenic mechanism is linked to the reprogramming of lipid metabolism in tumor cells, which affects the synthesis of key metabolites such as phosphatidylcholine and other glycerophospholipids. These metabolites are thought to significantly modulate cellular signaling and intercellular communication within the TME. Additionally, infection with colibactin-producing *E. coli* may impair the infiltration of CD8^+^ T cells and inhibit IFN-γ production, thereby creating an immune-suppressive environment that enables tumors to evade immune detection.^304^ In virus-related studies, microbial communities such as EBVs can strengthen the fatty acid synthesis pathway via the viral protein LMP1. It may further provide essential substrates for lipid synthesis and energy storage, either through direct viral metabolites or by stimulating lipid biosynthesis in host cells.

### Amino acid metabolism

Like glutaminase, tumor cells can catalyze the deamination of glutamine into glutamate, which serves as a vital nitrogen source for the biosynthesis of various nonessential amino acids.^305^ The microbiota can interfere with nitrogen metabolism within the TME by modulating glutamine processing, thereby promoting protein synthesis and tumor cell proliferation.^306^

Using 16S rRNA sequencing, Xue and colleagues characterized bacterial profiles in 47 pairs of hepatocellular carcinoma and adjacent liver tissues. Their research aimed to uncover the relationships among bacterial communities, differentially expressed genes, and metabolites.^307^ KEGG pathway enrichment analysis revealed that distinct metabolites are involved primarily in amino acid metabolism, including arginine and proline metabolism; purine and pyrimidine metabolism; and fatty acid biosynthesis.^308^ Similarly, based on 16S amplicon sequencing, as well as the eggNOG and KEGG databases, Xiaoqiang Chai et al. implemented bacterial functional predictions. These functional analyses highlighted metabolism as the predominant biological function of the bacterial communities, with amino acid metabolism emerging as the most prominent pathway.^309^

In another study carried out by Camila, *F. nucleatum* facilitated tumor cell growth via the L-glutamate degradation pathway, with a metabolic byproduct of butyrate playing a pivotal role in enhancing tumor spheroid growth.^310^ Moreover, the intricate relationship between bacteria and amino acid metabolism is evident in their ability to modulate immune responses against tumors through dietary amino acid intake. For example, *Lactobacillus johnsonii* (*L. johnsonii*), a commensal gut bacterium, collaborates with *Clostridium sporogenes* to convert dietary tryptophan into indole-3-propionic acid (IPA), a gut microbiota-derived metabolite. Furthermore, IPA significantly enhanced the acetylation of H3K27 at the superenhancer region of the *Tcf7* gene. This epigenetic modulation might influence the stem cell program of CD8^+^ T cells and promote the differentiation of precursor exhausted T cells. By enhancing the synthesis of the tryptophan-derived metabolite IPA, this process further augmented the activity of precursor exhausted T cells and improved the efficacy of ICB therapy in cancers such as melanoma, breast cancer, and colorectal cancer. We anticipate that *L. johnsonii* and bacterially derived IPA hold promise as potential adjuvants for personalized anticancer immunotherapy, offering new avenues to improve therapeutic outcomes.^311^

In a prior experiment exploring the impact of dietary tryptophan levels on the antitumor effects mediated by *L. reuteri*, mice were fed diets with varying tryptophan concentrations prior to tumor cell implantation. Compared with the control group, the tryptophan-rich diet group (1.19%) presented significantly greater tumor suppression and improved survival rates. This study revealed that a high-tryptophan diet could increase aryl hydrocarbon receptor activity within the TME to bolster antitumor immunity. Specifically, the metabolism of tryptophan by *L. reuteri* can increase the level of indole-3-aldehyde within tumors, which in turn stimulates IFN production by CD8^+^ T cells and increases the efficacy of ICIs.^161^

In Kaposi’s sarcoma, cells infected with KSHV depend on glutamine and asparagine to supply nitrogen to increase the biosynthesis of purines and pyrimidines.^312^ Through high-throughput RNA sequencing, KSHV infection has been demonstrated to upregulate the expression of several enzymes involved in the glutamine pathway, including glutaminase 2, glutamate dehydrogenase 1, and aspartate aminotransferase 2. Accordingly, KSHV may exploit host cell glutaminolysis to facilitate the proliferation of KSHV-transformed cells.^312^

In addition to their effects on primary metabolic processes and products, numerous microbial metabolites can also interact with tumor cells to significantly affect their developmental trajectories. For example, alcohol consumption may produce potential toxicity, which makes it a well-established risk factor for cancer, partially attributed to the oxidation of ethanol into acetaldehyde, a class 1 carcinogen that drives mutagenesis by binding directly to DNA.^313^ Compared with strains isolated from noncancer individuals, *Candida* species isolated from patients with oral cancer presented increased acetaldehyde production from ethanol.^314^ Moreover, strains derived from oral cancer patients presented heightened virulence characteristics, such as increased biofilm formation and hydrolytic enzyme production. Inosine, a nucleoside involved in purine metabolism, is another microbial metabolite with significant implications for cancer biology. In the TME, which is characterized by limited glucose availability, inosine can meet the energy requirements of antitumor T cells. Notably, in a mouse model of colorectal cancer, inosine produced by *Bifidobacterium pseudolongum* (*B. pseudolongum*) was found to improve the immunotherapy response and enhance antitumor immunity.^199^ Additionally, gut microbes can ferment dietary components to release various bioactive polyphenols, i.e., plant-derived compounds found in fruits and vegetables. Polyphenols have definite and potent anticancer effects despite their structural diversity and limited bioavailability.^315^ In addition, isoflavones, a type of polyphenol present in soy and soy products, are metabolized by gut bacteria into equol, a compound associated with a reduced risk of colorectal cancer and a strong inhibitory effect on colorectal cancer cells.^316^

## Interactions between the microbiota and TME components

**Fig. 6:**
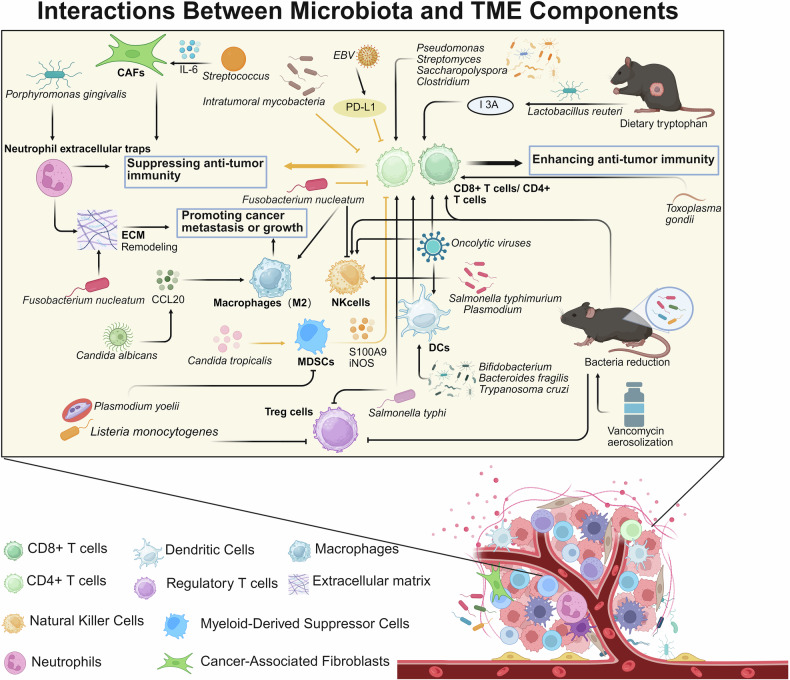
Interactions between the microbiota and TME components. The tumor microenvironment is orchestrated by a complex interplay of microbiota, extracellular matrix components, immune cells, the vasculature, and a myriad of signaling molecules. Diverse microbes within the body play various roles, with some promoting tumor metastasis and proliferation and others enhancing antitumor immune responses, working together with the host to combat cancer. I3A indole-3-aldehyde, iNOS inducible nitric oxide synthase. Created in BioRender. Wang, Y. (2025) https://BioRender.com/4zek8ww

Within the TME, tumor growth, invasion, and metastasis may be significantly influenced by the interplay among extracellular matrix components, immune cells, blood vessels, and diverse signaling molecules.^317^ Notably, the microbiota has recently gained attention for its profound impact on the TME. In addition to colonizing tumor tissues, these microorganisms may also interact with various elements of the TME to shape tumor progression^16^ (Fig. 6 and Table 1).

**Table 1:** Interactions between the microbiota and tumor microenvironment components

Microbiota	TME component	Mechanism of action	Impact on tumor	Ref
*Fusobacterium nucleatum*	T cells	Reduce the infiltration of tumor-infiltrating T cells	Promote tumor growth and metastasis	^484^
Intratumoral mycobacteria	T cells	Induce dysfunction of CD8^+^ tissue-resident memory cells	Promote tumor progression	^324^
EBV	T cells	High expression of PD-L1, inhibiting T-cell activity.	Promote tumor progression	^326^
Coriobacteriaceae	T cells	Inhibiting the infiltration of CD8+ tumor-infiltrating lymphocytes	Inhibiting the anti-tumor immune response	^346^
Pasteurella	T cells	Inhibiting the infiltration of CD8^+^ tumor-infiltrating lymphocytes	Enhancing the anti-tumor immune response	^346^
Bifidobacterium pseudolongum	T cells	The generated inosine induces the expression of Th1 regulatory genes in CD4^+^ T cells.	Enhancing the efficacy of anti-tumor immunotherapy	^327^
*Lactobacillus reuteri*	T cells	The release of indole-3-aldehyde enhances CD8^+^ T-cell-mediated immunity.	Inhibiting tumor growth and enhancing the efficacy of ICIs.	^176^
Plasmodium spp.	natural killer cells	Upregulating the expression of CD69 and CD25, and inducing the activation of natural killer cells.	The release of IFN-γ by natural killer cells triggers their cytotoxicity and activates tumor antigen-specific T cells.	^333^
*Bacteroides fragilis*	dendritic cells	Promoting the maturation of dendritic cells and stimulating IL-12-dependent Th1 cell responses.	Enhancing the anti-tumor efficacy of CTLA-4 blockade.	^338^
Bifidobacterium	dendritic cells	Enhancing the STING/IFN-β signaling pathway.	Enhancing T-cell-mediated anti-tumor responses.	^286^
*Fusobacterium nucleatum*	M2 macrophages	Polarizing M2 macrophages via a TLR4-dependent mechanism and promoting their infiltration through the NF-κB/miR-1322/CCL20 cascade	Promoting the growth and metastasis of colorectal cancer.	^343^
*Malassezia globosa*	Macrophages	Triggering M2 polarization of macrophages via the TLR4/NF-κB signaling pathway	Promoting tumor growth.	^176^
*Candida albicans*	Macrophages	Inducing Th17 cells to produce IL-17A, which activates the IL-17RA signaling pathway in tumor cells, leading to the release of CCL2 that recruits TAMs.	Promote tumor immune evasion and growth.	^344^
*Acinetobacter seifertii*	M1 macrophages	Promote the expression of M1 macrophage markers (TNF-α, iNOS), while inhibiting the migratory capacity of macrophages.	Exerts a dual impact on the activation and function of M1 macrophages, potentially diminishing their anti-tumor effects.	^345^
Pasteurella	M2 macrophages	Inhibit the infiltration of M2 macrophages.	Inhibit tumor progression	^346^
Coriobacteriaceae	M2 macrophages	Promote the infiltration of M2 macrophages	Promote tumor progression	^346^
*Fusobacterium nucleatum*	Tregs	Stimulate the production of CCL20, thereby promoting the infiltration of Tregs in TME.	Drive immunosuppression and accelerate tumor progression.	^347^
*Salmonella typhi* CVD 915	Tregs	Reduce the activity of Tregs while enhancing the secretion of IFN-γ.	Enhance the anti-tumor immune response.	^349^
*Plasmodium yoelii*	Tregs MDSCs	Release exosome-like vesicles to inhibit the recruitment of Tregs and MDSCs.	Reduce immune suppression and enhance anti-tumor immune responses.	^350^
*Candida tropicalis*	MDSCs	Recruit and activate MDSCs, thereby promoting the expression of S100A9, arginase-1, and iNOS.	Inhibit the activity of CD8+ and CD4 + T cells, thereby promoting immune suppression.	^485^
*Listeria monocytogenes*	MDSCs Tregs	Reduce the abundance and suppressive activity of MDSCs and Tregs, and reprogram MDSCs to produce IL-12.	Enhance the anti-tumor immune response.	^355^
*Porphyromonas gingivalis*	Neutrophils extracellular matrix	Stimulate the formation of neutrophil extracellular traps; induce fibrotic changes in extracellular matrix, thereby sequestering cytotoxic T cells within a dense stromal barrier.	Promote tumor cell invasion and impede anti-tumor immune responses.	^360,486^
*Helicobacter pylori*	extracellular matrix	Facilitate the degradation of extracellular matrix through the upregulation of MMP and the secretion of urease.	Promote tumor progression.	^361^
*Fusobacterium nucleatum*	CAFs	Interact with CAFs, induce the secretion of pro-inflammatory cytokines and chemokines by CAFs, thereby recruiting TAMs with a tumor-promoting phenotype.	Suppress the anti-tumor immune response.	^365^

The use of oncolytic viruses, a unique class of viruses that selectively infect and destroy cancer cells, has demonstrated their ability to effectively enhance systemic antitumor immunity. Within the TME, tumor lysis mediated by oncolytic viruses may stimulate the release of inflammatory cytokines and the expression of damage-associated molecular patterns. It may further increase the frequency and cytotoxic activity of peripheral natural killer cells and CD8^+^ T cells. During tumor lysis, the release of proinflammatory factors and chemokines, including IFN, TNF-α, IL-12, and IL-6, can activate macrophages, dendritic cells, and T cells through autocrine and paracrine signaling mechanisms.^318^ Furthermore, activated dendritic cells can enhance the presentation of viral antigens, whereas natural killer cells play a critical role by targeting oncolytic virus-infected tumor cells. Interestingly, certain fungal and parasitic infections within tumors can elicit similar immune-modulatory effects.van Tong, Brindley, Meyer and Velavan.^319,320^ Collectively, the above interpretations underscore the potential of the microbiota to influence immune evasion, metabolic reprogramming, and resistance to therapeutic interventions. To improve our knowledge of cancer biology and identify novel therapeutic targets, further efforts are needed to deepen our comprehensive understanding of the intricate interactions between the microbiota and TME components.

### T cells

The presence of an intratumoral microbiome may significantly influence T-cell function, particularly cytotoxic T-cell activity. Generally, a higher bacterial load within tumors is correlated with reduced T lymphocyte infiltration. In a study on nasopharyngeal carcinoma tissues, tumors with lower bacterial loads exhibited increased infiltration of CD8^+^ T cells, natural killer cells, and cytotoxic T lymphocytes than those with higher bacterial loads.^321^ In murine models treated with vancomycin/neomycin aerosolized, a reduction in bacterial presence in the lung led to a decrease in regulatory T cells (Tregs), increased activation of T cells and natural killer cells, and a diminished incidence of melanoma lung metastasis.^322^ Additionally, elevated levels of SCFAs in the lung, which are produced by anaerobic bacteria in the lower airways, suppress the production of IFN-γ^+^CD4^+^ and IFN-γ^+^CD8^+^ T cells, contributing to effector T-cell exhaustion and facilitating tumor growth.^323^ Further studies in OSCC and colorectal cancer have demonstrated that bacteria-positive tumors exhibit decreased expression levels of CD4 and CD8 but upregulated expression levels of immune-suppressive molecules such as CTLA-4 and ARG1.^37^ *F. nucleatum* notably reduced tumor-infiltrating T-cell infiltration, which facilitated the growth and metastasis of breast cancer. Similarly, in gastric cancer, intratumoral mycobacteria can induce dysfunction in CD8^+^ tissue-resident memory cells within the TME, thereby advancing tumor progression.^324^

Simultaneously, by binding to Dectin-1, commensal fungi have been shown to increase the population of tumor-promoting macrophages and reduce T-cell counts, thereby suppressing the radiotherapy-mediated antitumor immune response.^325^ Moreover, compared with EBV-negative gastric cancer, EBV-associated gastric cancer expresses PD-L1 more frequently.^326^ Mechanistically, PD-L1 can interact with PD-1 on the surface of T cells, delivering inhibitory signals that suppress T-cell activity and impair their ability to recognize and eliminate tumor cells effectively. Conversely, the tumor microbiome can contribute to antitumor immunity by promoting the activation of T and natural killer cells. For example, the presence of bacteria (e.g., *Pseudomonas*, *Streptomyces*, *Saccharopolyspora*, and *Clostridium*) within pancreatic adenocarcinoma tissues enhances the recruitment and activation of CD8^+^ T cells, thereby bolstering the antitumor immune response. Furthermore, increased densities of CD8^+^ T cells and granzyme B+ cells were observed in patients with long-term survival, highlighting the critical role of adaptive immunity in achieving favorable outcomes. Moreover, compared with short-term survivors, long-term survivors of pancreatic cancer presented a tumor microbiome characterized by greater alpha diversity, suggesting a potentially pivotal role of microbial diversity in shaping the tumor immune microenvironment and influencing patient survival.^282^

To investigate the relationship between intratumoral bacteria and CD8^+^ T-cell infiltration, Gongjian Zhu et al. analyzed 369 cutaneous melanoma patients via advanced algorithms and stringent bacterial identification criteria. Their findings revealed 18 bacterial genera associated with infiltrating CD8^+^ T cells, two of which (*Algibacter* and *Epilithonimonas*) were negatively correlated, whereas the other 16 genera were positively correlated.^282^ In mouse models, intratumoral *L. reuteri* was observed to colonize and persist within melanoma, releasing the dietary tryptophan metabolite indole-3-aldehyde. This metabolite can enhance CD8^+^ T-cell-mediated immunity, increase IFN-γ production, and improve the efficacy of ICIs.^161^ Additionally, a *Clostridium*-derived metabolite, trimethylamine N-oxide, can activate the endoplasmic reticulum stress kinase protein kinase RNA-like endoplasmic reticulum kinase, leading to gasdermin E-mediated pyroptosis in tumor cells and promoting CD8^+^ T-cell-driven antitumor responses. *B. pseudolongum*-produced inosine induced the expression of Th1 regulatory genes in CD4^+^ T cells in a colorectal cancer mouse model, further enhancing antitumor immunotherapy.^327^ Moreover, Pushalkar et al. reported that the PDAC microbiome could modulate the host immune system to exert carcinogenic effects. Specifically, the PDAC microbiome can activate TLRs, suppress the activation of CD4^+^ T cells and CD8^+^ T helper (Th) 1 cells, and inhibit M1 macrophage differentiation, thereby inducing a tolerogenic immune program. Removal of the PDAC microbiome reshaped the TME and improved the efficacy of immunotherapy, suggesting a potential therapeutic strategy for treating PDAC. However, other studies present contrasting findings. For example, in a study exploring the microbial composition and function in PDAC patients via 16S rRNA gene sequencing, specific bacterial genera, including *Saccharopolyspora*, *Pseudomonas*, and *Streptomyces*, promoted antitumor immune responses by recruiting and activating CD8^+^ T cells. These findings may suggest dual and often contradictory roles of the PDAC microbiome in affecting immune responses and disease progression.^328^ Overall, the effects of intratumoral bacteria are complex across different tumor types, illustrating their varied impacts on immune cell activity. At this stage, further tumor-specific investigations are needed to harness microbial interactions for therapeutic purposes.

In addition to intratumoral bacteria and fungi, exogenous parasitic infections can also modulate tumor-associated immunity. A study in 2022 demonstrated that the combination of an attenuated *T. gondii* strain that does not replicate Toxoplasma uracil auxotrophs and anti-programmed death 1 therapy significantly increased CD8^+^ T-cell infiltration in the TME of patients with PDAC.^329^ This effect is mediated by the secretion of IL-12 by dendritic cells, which also stimulates the production of IFN-γ within the TME. Therefore, certain parasites can increase T-cell infiltration and improve the immunogenicity of tumors. Similarly, increased proportions of CD4^+^ and CD8^+^ T cells were detected in mice immunized with attenuated live *Leishmania* parasites, indicating a robust cellular immune response.^330^

### Natural killer cells

The microbiota in cancer can modulate the cytotoxicity and immune surveillance functions of natural killer cells, potentially creating an immunosuppressive TME. For example, *F. nucleatum* expresses the Fap2 protein, which interacts with T-cell immunoglobulin and immunoreceptor tyrosine-based inhibition motif domain proteins on immune cells such as natural killer and T cells, thereby inhibiting natural killer cell-mediated lysis of human tumor cells. Conversely, certain bacteria can increase the abundance and activity of natural killer cells within the TME. CD11c^+^ cells containing *Salmonella*-derived flagellin can activate natural killer cells to produce IFN-γ via the IL-18–IL-18 receptor and MyD88 pathways, independent of TLRs. *Salmonella typhimurium* (*S. typhimurium*) engineered to express IL-2 can induce both local and systemic proliferation of natural killer cells, effectively suppressing the metastasis of osteosarcoma.^331^ Parasitic infections, such as *Plasmodium infections*, can also influence natural killer cell activation.^332^ Through the upregulation of CD69 and CD25 expression, *Plasmodium* infection can induce the activation of natural killer cells, triggering their cytotoxicity via IFN-γ production. Once activated, natural killer cells can target and kill specific lung cancer cells, leading to the release of tumor antigens. Consequently, this release drives the systemic activation of tumor antigen-specific T cells in the peripheral blood, spleen, and lymph nodes.^333^ Interestingly, a high-salt diet can increase the abundance of *Bifidobacterium*, with these bacteria localizing within tumors and enhancing natural killer cell function by increasing the levels of the metabolite hippurate, thereby inducing melanoma regression.^334^ In addition, existing experiments in mouse models have shown that bacteria engineered to carry immunostimulatory molecules, such as IL-18 or endosialin DNA vaccines, can increase the presence and cytotoxicity of natural killer and T cells in tumors.^335^

### Dendritic cells

Dendritic cells, as professional antigen-presenting cells, are vital in initiating and polarizing tumor antigen-specific immunity. Despite the lack of direct killing effects on tumor cells,^336^ dendritic cells can extract and transport specific tumor antigens to activate T cells, thereby forming the cornerstone of antitumor immune responses.^336^ By regulating the antigen-presenting function of dendritic cells, certain microorganisms have been confirmed to modulate T-cell activation. Specifically, *Bifidobacterium*, a gut microorganism, can selectively localize to tumor sites, where it enhances STING/IFN-β signaling in intratumoral dendritic cells. This mechanism further strengthens T-cell-mediated antitumor responses in murine models.^286^ A genetic modification of the *E. coli* strain Nissle 1917 (*EcN*) to deliver cyclic di-AMP, a STING agonist, has been shown to increase antigen presentation by dendritic cells and induce type I IFN production in mice. This enhanced dendritic cell activity promotes robust T-cell activation and reinforces antitumor immunity.^337^ Similarly, by promoting dendritic cell maturation and stimulating IL-12-dependent Th1 cell responses, *B. fragilis* has been found to increase the antitumor effects of CTLA-4 blockade.^338^ Moreover, some protozoan parasites can also facilitate dendritic cell maturation and activation to play a role in immune responses. Ligands from parasites, such as glycosylphosphatidylinositol from *Leishmania major*, *Trypanosoma cruzi*, and *Plasmodium falciparum*, as well as profilin-like proteins from *T. gondii*, have been revealed to activate dendritic cells via TLR binding.^339,340^ In a mouse model in which Lewis lung carcinoma cells were implanted, malaria infection enhanced T-cell activation and dendritic cell maturation by increasing the expression of CD80 and CD86.^341^ These findings indicate that parasites can directly impact dendritic cell function and indirectly shape immune responses by modulating T-cell activity.

### Macrophages

TAMs represent a plastic and heterogeneous cell population within the TME. Currently, TAMs are broadly categorized into two phenotypes, M1 and M2, with their own specific functions. Classically, the M1 phenotype is proinflammatory and characterized by the secretion of cytokines such as IL-1, which can stimulate antitumor immune responses and inhibit tumor growth. In contrast, the M2 phenotype has an oncogenic nature by promoting tumor growth, invasion, and immune suppression. During the progression of cancers, the microbiota may regulate macrophage polarization and thus alter their functional roles in immune responses. Existing data indicate that by modulating the behavior of TAMs, the microbiota within primary tumor tissues functions significantly in shaping the immunosuppressive environment in the TME.^342^ For example, tryptophan-derived metabolites produced by *Lactobacillus* can induce immunosuppression by influencing macrophage activity within the tumor, further supporting tumor growth and immune evasion.^342^

In colorectal cancer, *F. nucleatum* drives the polarization of M2 macrophages and stimulates tumor growth through a TLR4-dependent mechanism. By activating the NF-κB/miR-1322/CCL20 cascade, *F. nucleatum* promotes the polarization and infiltration of M2 macrophages to consequently enhance colorectal cancer metastasis.^343^ In oral cancer, infection with *C. albicans* induces the production of IL-17A by Th17 cells. Activation of the IL-17RA pathway in tumor cells leads to the release of C-C motif chemokine ligand 2 (CCL2), which attracts macrophages to the TME. These macrophages acquire an immunosuppressive phenotype that induces tumor immune evasion and growth.^344^ Furthermore, *Acinetobacter seifertii* (*A. seifertii*), a bacterial species negatively associated with M1 macrophages, can impair macrophage migration, potentially influencing the composition and function of the TME.^345^ In experiments involving *A. seifertii* and ID8 ovarian cancer cells, compared with untreated cells, *A. seifertii*-treated cells presented significantly elevated expression of M1 macrophage markers, includin*g* TNF-α and iNOS. However, as indicated by subsequent Transwell assays, *A. seifertii* reduced macrophage migration, particularly in the ID8-As-10-cell group. These findings suggest a dual role for *A. seifertii*: while it promotes the expression of M1 macrophage activation markers, it concurrently inhibits macrophage migration, revealing a negative correlation with M1 macrophages.^345^

In another mouse study, *Pasteurella* was positively correlated with cytotoxic CD8^+^ tumor-infiltrating lymphocytes and negatively correlated with M2-like macrophages. Conversely, *Coriobacteriaceae* exhibited the opposite pattern, with a negative correlation with CD8^+^ tumor-infiltrating lymphocytes and a positive correlation with M2-like macrophages. These contrasting associations influence the progression of lung tumors, underscoring the diverse roles of the tumor microbiota in modulating immune cell activity and shaping tumor development.^346^

### Regulatory T cells

Tregs, a subset of immunosuppressive T cells, are recruited to the TME through interactions with chemokines secreted by various tumors. Specific microorganisms can enhance the suppressive properties of Tregs, thereby inhibiting antitumor immune responses. For example, in esophageal cancer, *F. nucleatum* promotes tumor progression by stimulating the production of CCL20, facilitating the recruitment of Tregs into the TME and promoting immune suppression.^347^

Tregs are also found in the peripheral blood and tumors of patients with HNSCC. Notably, patients with HPV-positive HNSCC exhibit a greater frequency of intratumoral Tregs than do those with HPV-negative HNSCC.^348^ Conversely, certain microorganisms can counteract the immunosuppressive functions of Tregs to enhance antitumor immunity. For example, the highly immunogenic strain CVD 915 of *S. typhi* could reduce Treg activity while enhancing the functionality of CD4^+^ and CD8^+^ T cells that secrete IFN-γ in lymph nodes near the tumor site.^349^ Similarly, in tumor-bearing mice infected with *Plasmodium yoelii*, malaria infection suppressed major pathways that recruited Tregs to the TME by releasing exosome-like vesicles, resulting in the downregulation of cytokines and growth factors involved in Treg and MDSC recruitment.^350^ Additionally, a modified *Listeria monocytogenes* (*L. monocytogenes*) strain (ΔactA/ΔinlB) also reduced the population of Tregs in the TME of CT26 tumor-bearing mice. This reduction increased the ratio of CD8^+^ T cells to Tregs, thereby increasing antitumor immune responses.^351^

### Myeloid-derived suppressor cells

MDSCs are a heterogeneous population of immunosuppressive cells that play pivotal roles in promoting tumorigenesis and tumor progression. These cells are broadly classified into granulocytic and monocytic MDSCs, both of which can suppress antitumor immune responses. Notably, *F. nucleatum* has been identified as a key modulator of the tumor immune landscape by promoting the selective accumulation of diverse myeloid cell populations, including CD11b^+^ myeloid cells, MDSCs, TAMs, conventional myeloid dendritic cells, and CD103^+^ regulatory dendritic cells. This orchestrated accumulation fosters an immunosuppressive TME to promote tumor development.^352^ Fungal dysbiosis may also induce MDSC-mediated tumor progression. In colorectal cancer patients, the abundance of MDSCs at the tumor site positively correlates with the fungal load.^353^ In *caspase recruitment domain 9* (CARD9) −/− mice, fungal overgrowth results in the accumulation of MDSCs in the colon and further exacerbates colorectal cancer progression. Further treatment with fluconazole, an antifungal drug, in these mice inhibited colorectal cancer development, an effect linked to reduced MDSC accumulation.^354^ Additionally, *C. tropicalis* recruits and activates MDSCs in colorectal cancer tissues, promoting the expression of immunosuppressive mediators such as S100A9, arginase-1, and iNOS. These factors can inhibit the activity of CD8^+^ and CD4^+^ T cells, triggering immune suppression within the TME. Conversely, specific bacterial strains can counteract the immunosuppressive functions of MDSCs. For example, *L. monocytogenes*, which produces listeriolysin O, can reduce the abundance and suppressive activity of MDSCs and Tregs within the TME. Listeriolysin O also reprograms MDSCs to produce the immunostimulatory cytokine IL-12, thereby enhancing antitumor immune responses in diverse murine cancer models.^355^

### Neutrophils

Neutrophils, which reside primarily in the systemic circulation, are highly mobile cells capable of rapidly infiltrating tissues, making them the first responders to sites of injury. In the TME, tumor-associated neutrophils may infiltrate tumors and significantly promote tumor growth.^356^ Tan et al. reported that *P. gingivalis* plays a pivotal role in the tumor immune microenvironment by stimulating the formation of neutrophil extracellular traps.^217^ Their study further revealed that across a range of tumors, the proportion of neutrophils increased significantly in samples expressing Fas ligand. Notably, in the consensus molecular subtype classifications, consensus molecular subtype 4 tumors, characterized by their mesenchymal phenotype, exhibited the strongest correlation between Fas ligand expression and neutrophil incidence. This relationship might be attributed to elevated levels of CXCL8, a key neutrophil chemoattractant, within this subtype.^357^ Further investigations into the interaction between intratumoral bacteria and immune or epithelial cancer cells were conducted by coculturing colorectal cancer epithelial spheroids with *F. nucleatum* isolates obtained from colorectal cancer patients. These spheroids were encapsulated in a collagen matrix uniformly distributed with neutrophils. In the absence of *F. nucleatum*, neutrophils displayed unrestricted migration within the spheroids under live-cell confocal microscopy. However, the presence of *F. nucleatum* impaired neutrophil migration, causing neutrophil retention within infected cancer cell spheroids. This recruitment and retention of neutrophils in response to *F. nucleatum* suggested that the intratumoral microbiota actively shaped the neutrophil-enriched microecological niches in tumors colonized by bacteria, as evidenced by spatial analysis. The formation of neutrophil clusters coincided with significant increases in the phosphorylation of extracellular signal-regulated kinase and p38 MAPK pathways, both of which are activated in response to *F. nucleatum*. Therefore, the activation of these pathways within tumor microecological environments might be partially driven by myeloid reactions to intratumoral bacteria.^37^

### Extracellular matrix

Certain microbial populations within tumors can profoundly shape the remodeling of the extracellular matrix, a process that is pivotal to tumor progression and metastatic dissemination. These microbiota can either secrete or induce the production of various enzymes (e.g., collagenases, elastases, and hyaluronidases) that degrade extracellular matrix components, thereby facilitating cancer cell invasion and migration.^358^ In colorectal cancer, for example, *F. nucleatum* promoted extracellular matrix remodeling by enhancing the activation of critical pathways, including extracellular signal-regulated kinase and p38 MAPK, two well-established mediators of cell migration, tumor invasion, and immune suppression.^359^ In pancreatic cancer, *P. gingivalis* leads to extracellular matrix reconstruction through the activation of neutrophil extracellular traps. In addition to inducing fibrotic changes in the extracellular matrix to promote tumor cell invasion, these neutrophil extracellular traps can also impede antitumor immune responses by entrapping cytotoxic T cells within the dense stromal matrix.^360,361^ Similarly, in gastric cancer, *H. pylori* facilitates extracellular matrix degradation by upregulating matrix metalloproteinases and secreting urease, thereby creating a microenvironment that favors tumor growth.^361^ Such extracellular matrix degradation is closely intertwined with chronic inflammation, which further promotes immune evasion and accelerates tumor progression.

### Cancer-associated fibroblasts (CAFs)

CAFs within the TME are recognized as central mediators of tumor progression through complex interactions with various cellular and microbial components. Notably, emerging evidence has revealed a novel dimension of these interactions, wherein specific intratumoral bacteria establish direct crosstalk with CAFs, significantly influencing tumor growth, immune evasion, and resistance to therapeutic strategies. In colorectal cancer, the B2 phylogroup *E. coli* was identified as a key contributor to tumor progression.^362^ Its bacterial strain produces the genotoxin colibactin to induce DNA damage, thereby promoting carcinogenesis. Furthermore, *E. coli* interacts with CAFs by stimulating their role in extracellular matrix remodeling, thus facilitating tumor growth. Coculture studies demonstrated that *E. coli* enhanced the tumor-supportive functions of CAFs, increasing the deposition of extracellular matrix components that promoted tumor cell invasion. This interaction is particularly relevant in colorectal tumors, where CAFs modify the extracellular matrix to create a favorable niche for bacterial persistence and proliferation, establishing a feedback loop that increases tumor aggressiveness.^362^ In ESCC, *Streptococcus* species are involved in CAF interactions through the secretion of IL-6,^363^ a cytokine with a pivotal role in promoting fibroblast activation. Activated fibroblasts, in turn, promote tumor development by suppressing antitumor immune responses.^364^ In this context, *Streptococcus* species are translocated from the gut to the esophageal tumor site, where they stimulate CAFs to produce IL-6. This interaction further fostered an immunosuppressive microenvironment that enhanced tumor growth and conferred resistance to treatment. Consequently, targeting IL-6 signaling represents a promising therapeutic approach for ESCC. In OSCC, *F. nucleatum*, a bacterium commonly found in the oral cavity, interacts extensively with CAFs. This bacterium induces CAFs to secrete proinflammatory cytokines and chemokines, which recruit TAMs with protumorigenic phenotypes. These TAMs further suppress antitumor immune responses and contribute to the establishment of an immunosuppressive TME. The interplay between *F. nucleatum* and CAFs has been associated with increased tumor invasiveness and poor prognosis in OSCC patients.^365^

Additionally, oncolytic viruses exert their effects by exploiting the crosstalk between CAFs and cancer cells to penetrate the densely collagenous extracellular matrix. Tumor cells secrete high levels of fibroblast growth factor 2 to increase their susceptibility to viral infection while also releasing transforming growth factor-beta 1 to facilitate oncolytic virus infection of CAFs. This dynamic interaction underscores the therapeutic potential of oncolytic viruses in overcoming the physical and immunological barriers within the TME.^366^

## Relationships between the microbiota and the treatment response of tumors

### Role of the microbiota in chemotherapy and radiotherapy

**Fig. 7:**
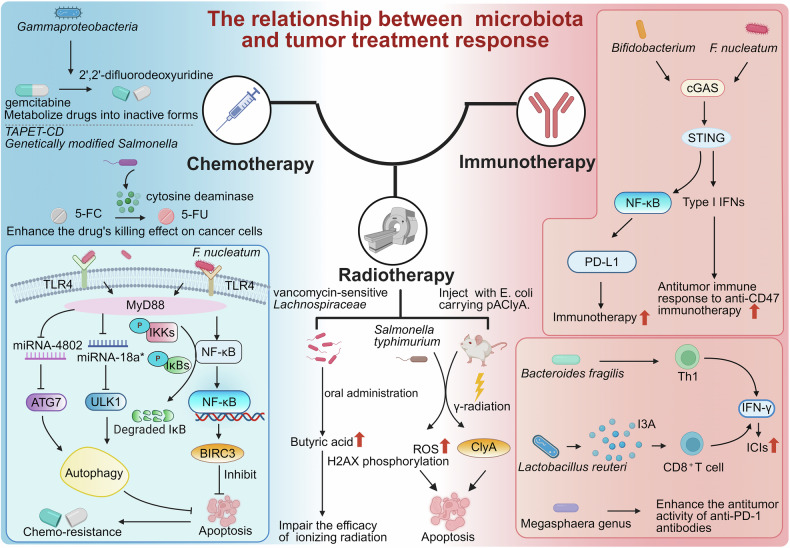
The complex interplay of the microbiota in cancer patients with different treatment modalities. Certain bacteria can metabolize drugs into inactive compounds, reducing their therapeutic impact, whereas others can convert prodrugs into active chemotherapeutic agents, increasing their antitumor properties. In addition, *F. nucleatum* can directly target tumor cells to increase drug resistance, which activates autophagy and evades the apoptosis induced by chemotherapeutic drugs. Combining specific bacteria with radiation can increase reactive oxygen species (ROS) production and induce tumor cell apoptosis. For immunotherapy, *Fusobacterium nucleatum* can activate the cGAS‒STING pathway, leading to the upregulation of PD-L1 expression in tumor cells, which may improve the efficacy of PD-L1 immunotherapy. MyD88 myeloid differentiation factor 88, 5-FC 5-fluorocytosine, 5-FU 5-fluorouracil, ROS reactive oxygen species, BIRC3 baculoviral IAP repeat containing 3, ClyA Cytolysin A, I3A indole-3-aldehyde. Created in BioRender. Wang, Y. (2025) https://BioRender.com/0 szoxnc

In combination with radiotherapy, chemotherapy, a common therapeutic option for cancer treatment, employs agents such as gemcitabine, fluorouracil, and platinum-based compounds. These chemotherapeutic agents function as genotoxic substances, inflicting damage to the DNA of cancer cells and concurrently disrupting the synthesis of new DNA during cell replication.^367^ However, the efficacy of chemotherapy is frequently compromised by the emergence of chemoresistance, which poses a significant challenge to successful cancer treatment. The mechanisms underpinning chemoresistance include genetic mutations, enhanced DNA repair mechanisms, epigenetic modifications, dysregulated apoptosis, altered autophagy, and changes within the TME.^368^ Existing findings have highlighted the presence of microorganisms within tumor tissues and their multifaceted roles in cancer progression. These intratumoral microbiota can significantly impact the efficacy of chemotherapeutic interventions, in addition to modulating the host immune response. Elucidation of the intricate interplay between the microbiota and chemotherapy can benefit the integration of microbiota-focused strategies into cancer therapy, thus mitigating drug resistance and improving treatment outcomes (Fig. 7).

Some microorganisms may trigger tumor drug resistance to stimulate chemoresistance actively. Sitthirak et al. categorized cholangiocarcinoma cells into resistant and sensitive groups on the basis of their response to gemcitabine and cisplatin. Their analysis of intratumoral bacterial species revealed distinct microbial compositions and abundances in resistant tumors compared with their sensitive counterparts.^369^ Metabolomic profiling further revealed elevated levels of metabolites such as acetylcholine and carnitine, compounds known to support tumor progression, in the resistant group.^370^ Furthermore, intratumoral bacteria can metabolize chemotherapeutic agents into inactive derivatives to exacerbate drug resistance. For example, in pancreatic tumors, Gammaproteobacteria expressing cytidine deaminase enzymatically converted gemcitabine, a common chemotherapeutic agent, into an inactive metabolite, 2’,2’-difluorodeoxyuridine, thereby diminishing its therapeutic efficacy. In addition, antibiotic treatment restored sensitivity to gemcitabine in a colorectal cancer mouse model, underscoring the pivotal role of intratumoral bacteria in mediating drug resistance.^371^

Conversely, certain bacterial enzymes may result in the biotransformation of chemotherapeutic agents into active antitumor agents to increase their efficacy. Nemunaitis et al. demonstrated that engineered *Salmonella* strains modified to express cytosine deaminase while attenuating virulence could convert 5-fluorocytosine, an antifungal agent, into the chemotherapeutic agent 5-fluorouracil within the TME. This innovative approach proved more effective than administering 5-fluorouracil alone, highlighting the potential of leveraging engineered bacteria as adjuncts to conventional chemotherapy.^372^

In addition to modulating the activity and concentration of drugs at the tumor site, microorganisms can modulate the development of drug resistance through direct interactions with tumor cells. For example, *F. nucleatum* has been implicated in enhancing chemoresistance in colorectal cancer. Yu et al. demonstrated the enrichment of *F. nucleatum* in tumor tissues from colorectal cancer patients who experienced postchemotherapy recurrence. By targeting TLR4 and MyD88, this bacterium can suppress the expression of two miRNAs, miR-18a* and miR-4802. The suppression of these miRNAs maintains active autophagy in tumor cells, enabling them to evade the apoptosis induced by chemotherapeutic agents such as 5-fluorouracil and oxaliplatin.^373^ Consequently, interventions targeting *F. nucleatum* could mitigate tumor chemoresistance. The presence of *F. nucleatum* may also induce chemoresistance via additional mechanisms. Recently, *F. nucleatum* was found to regulate the TLR4/NF-κB pathway, increasing the expression of BIRC3, a protein strongly associated with colorectal cancer resistance to 5-fluorouracil. Similarly, *E. coli* strains producing colicin promoted colorectal cancer progression and chemoresistance by altering the TME to an immunosuppressive, lipid-overloaded state.

The existing findings concerning the relationship between fungal communities and chemotherapy outcomes are limited, and some of these findings link fungal species to chemoresistance. For example, the colorectal cancer-associated pathogenic bacterium *C. tropicalis* has been shown to increase chemoresistance by producing lactic acid, which inhibits the mismatch repair protein MLH1. Conversely, inhibiting lactic acid production can restore mismatch repair protein expression via the GPR81-cAMP-PKA-CREB pathway, thereby reducing drug resistance.^374^ The impact of microbial communities on chemotherapy efficacy and toxicity can be interpreted on the basis of various mechanisms, including metabolic processes, enzymatic degradation of drugs, ecological alterations within the TME, and modulation of immune responses. Chemotherapeutic agents, in turn, can negatively impact the composition of healthy gut microbiota.^375^ For example, cyclophosphamide, an alkylating agent used in both hematological malignancies and solid tumors, can disrupt the small intestinal epithelium. This barrier damage triggers a microbiota dependent, tumor-specific Th cell-mediated antitumor response.^376^ Despite these insights, little is known about the mechanisms underlying these microbe‒chemotherapy interactions. Chemotherapy also raises additional concerns, such as the potential promotion of antibiotic-resistant gut bacteria, underscoring the need for continued research to elucidate and mitigate these effects.^309^

Similarly, radiotherapy is a cornerstone of cancer treatment that uses ionizing radiation to destroy the DNA of tumor cells locally, thereby inhibiting their proliferation and offering palliative care. However, the microbiota can also modulate the effectiveness of radiotherapy, with certain bacterial and fungal species either attenuating or enhancing therapeutic outcomes. For example, oral administration of vancomycin-sensitive *Lachnospiraceae* bacteria may reduce the efficacy of ionizing radiation, as it increases the levels of butyric acid in both systemic circulation and tumors. Conversely, specific bacterial elimination through antibiotic treatment can impair the effectiveness of chemotherapy, whereas fungal eradication appears to enhance radiotherapy outcomes. Commensal fungi, in particular, may dampen the immune response following radiotherapy by binding to the receptor Dectin-1, which increases the number of tumor-promoting macrophages and suppresses antitumor T-cell activity.^325^ Stephen et al. demonstrated the improved efficacy of radiotherapy by employing antifungal treatment with fluconazole in murine breast tumor models. While antifungal treatment did not alter tumor cell proliferation after radiotherapy, it significantly increased tumor cell death, delayed tumor growth, and improved survival rates. Accordingly, commensal fungi are critical in modulating the immune landscape of the TME during radiotherapy.^325^ Despite its therapeutic benefits, radiotherapy can cause severe side effects, such as bone marrow and gastrointestinal toxicity, which are collectively termed acute radiation syndrome, also called radiation toxicity.^37^ Importantly, the use of probiotics may mitigate radiation-induced gastrointestinal toxicity. In mouse models, FMT increased the production of prostaglandin F2α, which activated the MAPK/NF-κB axis, inhibited the apoptosis of irradiated cells, and alleviated pulmonary inflammation. Therefore, microbiota-based interventions are valuable for reducing the adverse effects of radiotherapy. Innovative strategies combining radiotherapy with microbial agents have demonstrated synergistic effects in preclinical studies.

Yoon et al. reported that attenuated *S. typhimurium* enhances radiotherapy efficacy by increasing ROS levels and inducing H2A histone family member X phosphorylation, which triggers tumor cell apoptosis via the caspase-3 and bcl-2 pathways.^377^ Similarly, Jiang et al. established a BALB/c mouse model bearing CT26 tumors to evaluate the antitumor effect of radiotherapy combined with a genetically engineered *E. coli* strain carrying the plasmid pAClyA. With the production of the cytotoxic protein ClyA, this strain was proven to significantly improve the therapeutic efficacy of radiotherapy, emphasizing the potential of microbial agents to improve radiotherapeutic outcomes.^378^

### Role of the microbiota in immunotherapy

In recent years, immunotherapy has emerged as a transformative adjunct to conventional cancer treatments for eliminating malignancies. To impede cancer progression, this therapy is designed to enhance the patient’s immune system or employ immune agents to specifically target cancer cells expressing foreign antigens. This advancement has profoundly influenced clinical cancer management. Notably, ICB therapy has revolutionized the treatment of various solid tumors, achieving overall response rates ranging from 15% to 20%.^379^ Intratumoral bacteria may enhance or hinder the efficacy of immunotherapy, highlighting their intricate role within the TME and their potential to interfere with treatment outcomes^286,380^ (Fig. 7). For example, clostridial bacteria were detected to be more abundant in melanomas of patients who respond to ICIs than in those of nonresponders.^381^ Similarly, in patients with locally advanced ESCC undergoing neoadjuvant chemotherapy combined with immunotherapy, Wu et al. revealed variations in bacterial α diversity between tumors and adjacent normal tissues, as well as between responders and nonresponders. Despite no significant difference in the total bacterial load, specific bacterial taxa were differentially abundant, with the presence of *Streptococcus* in the ESCC TME correlating with an activated tumor immune microenvironment and improved therapeutic efficacy of neoadjuvant chemotherapy combined with immunotherapy.^379^

Shi et al. reported that systemic administration of *Bifidobacterium* led to its accumulation within tumors, enhancing local responses to anti-CD47 immunotherapy through STING-dependent and IFN-dependent mechanisms. Moreover, *F. nucleatum*, a common pro-oncogenic bacterium in tumors, can activate the cGAS‒STING pathway, further leading to NF-κB signaling activation and PD-L1 expression upregulation in tumor cells. This increase in PD-L1 expression might increase the effectiveness of PD-L1 blockade therapies.^382^ In melanoma models, the probiotic *Lactobacillus reuteri* produces indole-3-aldehyde, a dietary tryptophan metabolite that enhances local IFN-γ production by CD8^+^ T cells, thus increasing antitumor immunity and improving the efficacy of ICIs.^161^ Similarly, the response to PD-1 blockade therapy was also positively correlated with an increased abundance of *Akkermansia* following FMT in mouse models, which was associated with increased recruitment of CCR9^+^CXCR3^+^CD4^+^ T cells.^383^ Clinical trials in melanoma have demonstrated that FMT can overcome resistance to anti-PD-1 therapy by reconfiguring the TME.^384^ Additionally, Sivan et al. reported that the administration of *Bifidobacterium* enhanced the antitumor efficacy of anti-PD-L1 therapy in a melanoma mouse model, which suppressed PD-1 expression, activated natural killer cells, and mediated tumor destruction via perforin and IFN-γ secretion.^385^ Similarly, the monoclonal antibody ipilimumab, which targets CTLA-4, a critical negative regulator of T-cell activation, benefits from interactions with *B. fragilis*. This bacterium could potentiate the efficacy of CTLA-4 blockade immunotherapy by stimulating Th1 immune responses.^338^ Furthermore, the administration of *Megasphaera* species to mice with 4T1 breast tumors, a therapy that prolongs the survival of PDAC patients, enhanced the antitumor effects of anti-PD-1 antibodies.^386^ However, not all microbial influences are beneficial. For example, in NSCLC patients receiving anti-PD-1 therapy, *H. pylori* suppressed antitumor CD8^+^ T-cell responses by impairing the cross-presentation activity of dendritic cells, thereby reducing the efficacy of this therapy.^387^

All the above findings underscore the importance of the microbiota in immunotherapy outcomes and support the potential of manipulating microbial communities to increase therapeutic efficacy. Oral antibiotics combined with ICIs represent a promising solution for improving therapeutic outcomes. It is highly important to comprehensively evaluate the interplay between the microbiota and cancer therapies, particularly chemotherapy and immunotherapy, which can provide critical insights for developing more effective and personalized treatment modalities. One of the primary concerns with antibiotic-based strategies is their potential off-target effects on the commensal microbiota, particularly in the gut. The gut microbiota is pivotal for maintaining immune homeostasis, modulating inflammation, and influencing the efficacy of ICIs. Systemic antibiotic administration may disrupt this microbial balance, leading to unintended immunosuppressive effects, as observed in studies where pretreatment with antibiotics was associated with reduced survival in cancer patients receiving ICIs.^388^ This highlights the need for precise delivery systems that selectively target the tumor-infiltrating microbiota while minimizing collateral damage to beneficial microbial communities. Potential strategies may include nanocarrier-based antibiotic delivery [e.g., endogenous proteo-ellagic acid nanocomplexes], which can restrict antibiotic activity to the tumor site.^389^ However, further optimization is needed to achieve effective tumor-infiltrating microbiome clearance without altering the systemic microbiota composition when these technologies are utilized.

## Clinical research progress in targeting the microbiota for cancer treatment

### Potential therapeutic approaches and targets

**Fig. 8:**
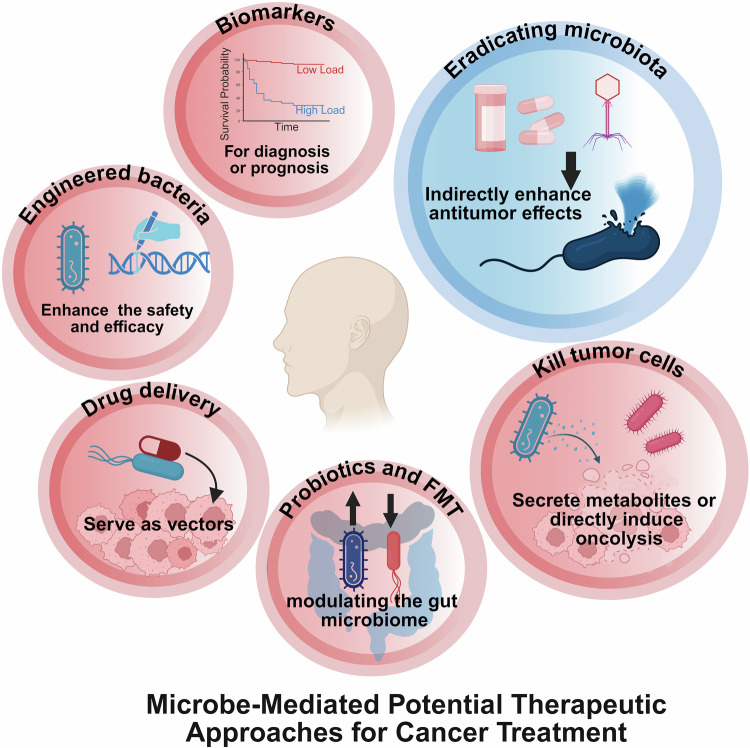
Potential therapeutic approaches and targets. The load or diversity of specific microorganisms can be utilized to predict therapeutic outcomes or to serve as biomarkers for diagnosis. Additionally, specific microbiota in cancer can combat tumors directly or indirectly. The use of bacteriophages or antibiotics to eradicate bacteria within tumors might become a potent approach for cancer therapy. FMT fecal microbiota transplantation. Created in BioRender. Wang, Y. (2025) https://BioRender.com/pum3c7u

Surgery, chemotherapy, and radiotherapy represent the fundamental pillars of cancer treatment. Each modality has its own unique advantages and limitations. While surgery is highly effective for localized tumors, it is often intricate and time intensive. On the other hand, chemotherapy and radiotherapy can achieve significant cancer control and progression inhibition but lack specificity, as their target is not only cancer but also healthy cells. The two therapies fall short in precisely targeting tumor areas, leading to risks of incomplete cancer cell removal and posttreatment recurrence. Furthermore, the adverse effects associated with these treatments can damage healthy DNA, potentially triggering secondary cancer transformations or drug resistance, thereby diversely compromising treatment outcomes. In light of these challenges, scholars are committed to pursuing more effective, safer, and cost-efficient cancer therapies. Microbial therapy has emerged as a promising area of research, integrating live bacteria with conventional treatments to address their inherent limitations. Advances in synthetic biology have revealed the role of bacteria within tumors, highlighting their intimate relationship with tumor progression and their potential as innovative predictive and diagnostic biomarkers. Certain bacterial strains engineered for reduced virulence demonstrate precise tumor-targeting capabilities, robust stimulation of antitumor immunity, and notable anticancer effects. These bacteria can act as drug carriers within the body, enabling localized delivery of therapeutic agents to tumor sites. Additionally, the targeted eradication of intratumoral bacteria via antibiotics also has therapeutic benefits. This dual utility, either leveraging bacteria for treatment or eliminating them to modify the TME, underscores the transformative potential of microbial therapy in the development of next-generation cancer treatments (Fig. 8).

#### Harnessing the microbiota for the diagnosis and treatment of cancer

##### *Biomarkers for diagnosis or prognosis*

Compared with healthy tissues, tumor tissues harbor a distinct microbiota, with specific bacterial species directly associated with carcinogenesis. The intratumoral microbiota has emerged as a promising biomarker for cancer screening.^109^ For example, individualized data derived from the intratumoral microbiota can distinguish patients with esophageal cancer^390^, pancreatic cancer^391^, lung cancer,^392^ and oral cancer from healthy individuals. In addition to cancer diagnosis, the microbiota profile also has certain value for predicting prognosis considering its variation according to tumor stage, tumor grade, cancer score, and genetic mutations (e.g., estrogen receptor, progesterone receptor, and HER2)^393^. Recently, increased diversity within the intratumoral microbiota has been revealed to correlate with improved survival rates in pancreatic cancer patients. A specific microbial signature, encompassing *Pseudomonas*, *Bacillus*, and *Streptomyces*, has been identified as a predictor of long-term survival in cancer patients.^282^ This was evident in a retrospective cohort study where a high bacterial load in nasopharyngeal carcinoma was linked to a less favorable prognosis, indicating the potential of the intratumoral bacterial load as a robust prognostic indicator for nasopharyngeal carcinoma patients.^321^ Moreover, in cases of colorectal cancer, *F. nucleatum* is associated with a microsatellite instability phenotype or BRAF mutations.^394^ In gastric cancer and colorectal adenoma, intratumoral *H. pylori* was related to increased disease risk and poorer disease status, which was positively correlated with the level of the CagA protein.^395^ In gastrointestinal tumors, the detection of *Candida* DNA can predict reduced survival rates. Narunsky-Haziza et al. proposed the prognostic and diagnostic potential of fungi, supported by significant insights obtained from comparisons of fungal communities with corresponding bacterial and immune profiles.^163^ For example, Gao et al. reported an elevated ratio of *Ascomycota* to *Basidiomycota* in patients with colorectal cancer compared with healthy controls, alongside the enrichment of fungal families such as *Microascaceae* and *Sordariaceae*.^396^ Patients with advanced colorectal cancer also presented a decrease in fungal families such as *Pleosporaceae*. With respect to alterations in fungal composition in other cancers, Banerjee et al. reported significant differences in the abundance of *Cladosporium* and *Pneumocystis* in ovarian cancer samples, suggesting potential prognostic implications.^397^ Collectively, these findings underscore the utility of the intratumoral microbiota as a biomarker for cancer diagnosis, prognosis, and stratification, offering potential guidance for therapeutic decision-making on the basis of patient risk profiles.

Notably, challenges remain despite the aforementioned advancements. The low microbial biomass within tumor tissues and the risk of false positives from environmental contaminants complicate microbiota-based diagnostics. Nevertheless, intratumoral bacteria and fungi can still serve as reliable biomarkers for cancer diagnosis and prognosis, benefiting from optimized sampling techniques and rigorous data analysis. Future advancements may involve integrating microbiome-based diagnostics with advanced imaging modalities to better predict tumor progression and patient outcomes. The spatial distribution and intracellular localization of these microbes can be mapped for more precise prognostication and tailored treatment strategies in clinical settings, ultimately improving cancer management.

##### *Exploiting the microbiota for anticancer therapy*

Chemotherapy, radiotherapy and other conventional anticancer modalities often face challenges, including poor tumor penetration, drug resistance, and tumor recurrence or metastasis. Inspired by natural systems, certain bacteria can provide a potent solution for addressing these limitations. After dispersing and thriving in the hypoxic core of tumors,^398^ these microorganisms can resensitize tumor cells to treatments and activate the immune system of the animal to achieve sustained anticancer effects.^399^ As early as 1891, William Coley reported the anticancer therapeutic potential of intratumoral microbes. A modern example of this approach is the use of *Bacillus Calmette-Guérin*, a standard treatment already applied for localized bladder cancer.^400^

The tumor-resident microbiota can induce cancer cell death through various mechanisms. Bacteria consume essential nutrients to support their proliferation within tumor cells or the TME, causing metabolic stress to trigger cancer cell death.^401^ In mouse models, intracellular *Salmonella* might retard tumor growth, as it can induce cancer cell apoptosis or autophagy^332^. Moreover, certain microorganisms secrete toxic metabolites that directly target tumor cells. For example, *Staphylococcus epidermidis* produces 6-N-hydroxyaminopurine, a molecule that inhibits DNA polymerase activity and selectively suppresses skin tumor proliferation in mice.^402^ Other microbial molecules also exhibit unique anticancer effects. For example, *P. aeruginosa*-produced azurin can suppress cancer progression, which is supported by its ability to stabilize p53 by inhibiting constitutive photomorphogenesis protein 1-mediated ubiquitination and inducing cell cycle arrest at the G2/M phase after penetrating tumor cells.^403^ Similarly, nisin A, an antibiotic, disrupts cancer cell membranes by altering their integrity and forming pores, leading to changes in the membrane potential and inhibition of tumor growth.^404^

Another widespread mechanism involves modulating immune infiltration to interfere with tumor growth. Flagellin, a component of bacterial flagella, was reported to inhibit cancer cell proliferation by activating TLR5.^405^ *L. monocytogenes* has direct cytotoxic effects on cancer cells by inducing NADPH oxidase activity and increasing intracellular calcium (Ca²⁺) levels, which drive ROS production.^406^ Furthermore, microbial therapies also target immunosuppressive pathways within the TME. For example, indoleamine 2,3-dioxygenase 1, which catabolizes tryptophan, results in immune tolerance by activating Tregs, deactivating effector T cells, and inducing immune cell apoptosis.^407^ In a melanoma mouse model, attenuated *S. typhimurium* expressing small interfering RNA targeting indoleamine 2,3-dioxygenase 1 led to extensive tumor cell death, accompanied by increased tumor infiltration by polymorphonuclear neutrophils.^408^ Similarly, *Streptococcus* abundance in the TME of ESCC is associated with an activated tumor immune microenvironment, enhancing the efficacy of neoadjuvant chemotherapy combined with immunotherapy.^379^ In addition to bacteria, parasites have demonstrated anticancer potential. Researchers at the University of Edinburgh Medical School reported that intratumoral injection of an attenuated strain of *Leishmania* in a mouse model of 4T1 breast cancer promoted M1 macrophage activation and the release of proinflammatory cytokines, inducing a protective Th1 immune response.^409^ A study by Nguyen et al. in a medulloblastoma mouse model revealed that infection with *T. gondii* attracted T cells to the tumor, transforming the TME into a T-cell-supportive environment. This shift was accompanied by increased IFN-γ levels and expression of IFN-γ-driven genes, indicative of a robust Th1 immune response.^410^

Recently, Japanese scientists isolated intratumoral bacteria with strong biocompatibility and immunogenic anticancer properties from colon cancer tissues, further advancing development in this field. These bacteria, related to natural purple photosynthetic bacteria (*A-gyo*, *UN-gyo*, and their combination, *AUN*), preferentially proliferate in tumor tissues, triggering robust immune cell infiltration and potent anticancer responses in various mouse cancer models, including those of colorectal cancer, sarcoma, metastatic lung cancer, and drug-resistant breast cancer. In addition to stimulating immune memory, these bacteria can also possess photosynthetic capabilities, enabling them to absorb specific wavelengths of light, such as near-infrared light, for growth and metabolism. This property can be leveraged to enhance tumor detection through fluorescence imaging, thereby improving the diagnostic sensitivity and specificity for cancers. Moreover, these bacteria can be cultured cost-effectively, addressing the economic and environmental concerns of traditional anticancer drugs.^285^ Oncolytic *Clostridium* strains have also demonstrated promising anticancer potential. These bacteria exhibit robust tumor colonization and lysis across multiple tumor types.^411^ For example, in a study of successfully treated advanced leiomyosarcoma, *Clostridium novyi-NT* spores resulted in significant tumor destruction, gas production, and extensive tumor lysis, as observed via CT scans. Notably, oncolytic bacterial therapy combined with other therapeutic modalities appears to be a more effective strategy, as its use alone may not completely eliminate tumor cells.^411^ However, it is worth noting that there may still be risks of tumor progression or recurrence. Considering that the mechanism by which C. novyi NT destroys tumor cells does not overlap with other treatment mechanisms, a combined therapy involving multiple treatment methods appears to be more effective.^412^

Oncolytic viruses have recently emerged as promising modalities in cancer immunotherapy, exhibiting significant potential for clinical application. While activating the host’s systemic antitumor immune response, oncolytic viruses can selectively infect and lyse tumor cells simultaneously, thereby enhancing immune surveillance against cancer. A notable example is Talimogene laherparepvec, a genetically engineered herpes simplex virus (HSV) modified to express granulocyte–macrophage colony-stimulating factor. Talimogene laherparepvec has demonstrated remarkable efficacy in clinical trials for melanoma. Similarly, other oncolytic viruses, such as the adenovirus H101, Teserpaturev (HSV-1), and Nadofaragene firadenovec (adenovirus), have shown promising therapeutic potential in various solid tumors.^413,414^ Oncolytic viruses can amplify their proinflammatory functions by modulating the polarization of TAMs, thereby fostering robust antitumor immune responses. For example, a genetically engineered HSV-1 expressing OX40L was used to enhance the proinflammatory phenotype of TAMs and synergized effectively with anti-IL-6 therapy. Furthermore, the application of oncolytic viruses combined with ICIs, such as anti-PD-1 and anti-CTLA-4 therapies, significantly augmented TAM-mediated antitumor activity, highlighting the potential of combinatorial approaches in immunotherapy.^415^ Despite these advancements, the interaction between oncolytic viruses and TAMs presents challenges. Indeed, TAMs can enhance proinflammatory responses and promote antitumor immunity. Activated TAMs may also facilitate the clearance of intratumoral oncolytic viruses, resulting in compromised therapeutic efficacy. Additionally, the specificity of oncolytic viruses remains a critical concern, as certain viruses lack tumor-specific targeting, potentially causing off-target replication and systemic toxicity.

The microbiota holds significant promise as a therapeutic agent, and genetic engineering and nanotechnology are needed during conventional anticancer treatments to increase the efficacy and safety of bacterial therapies. Native pathogenic bacteria tend to exhibit limited therapeutic properties and inherent pathogenicity, necessitating their attenuation and optimization for medical use. Advances in genetic and synthetic biology have enabled the customization of bacteria to enhance their anticancer efficacy and safety profiles. Specifically, attenuated bacteria can be engineered to selectively target tumors, induce cancer cell death, and inhibit tumor angiogenesis.

Strategically, key virulence genes can be eliminated, or nutrient-deficient mutant strains can be developed to achieve safe attenuated bacterial therapy. These modifications, while preserving therapeutic benefits, ensure a reduced risk of adverse effects. Furthermore, attenuated bacteria can potentiate the host’s antitumor immune response by promoting the proliferation and activation of cytotoxic T lymphocytes and natural killer cells, reducing the intratumoral population of Tregs, and neutralizing the immunosuppressive activity of MDSCs. An example of such an advance is VNP20009, an attenuated strain of *S. typhimurium* engineered through the deletion of the *msbB* and *purI* genes. This strain has been extensively studied in murine tumor models and has demonstrated specific tumor-targeting capabilities and tumor-suppressive effects.^416^ Moreover, the deletion of *msbB* reduces the myristoylation of the lipid A component of lipopolysaccharide, significantly lowering lipopolysaccharide-induced TNF production and the risk of infectious shock. However, clinical trials of VNP20009 in cancer patients revealed a lack of tumor specificity and limited therapeutic benefit, underscoring the need for further optimization.^417^ In addition to *S. typhimurium*, bacteria have been genetically modified to produce cytotoxic molecules that disrupt cancer cell function and induce apoptosis.^418^ For example, bacteria engineered to express cytolysin A inhibited tumor growth in murine models by forming pores in cancer cell membranes, leading to apoptosis.^378,419^ Similarly, genetically modified *L. monocytogenes* directly eliminates cancer cells by activating NADPH oxidase and increasing intracellular Ca²⁺ levels.^420^ *Listeria* strains could be tailored to target HER2-expressing tumor cells through biotin-streptavidin binding systems.^421^ Additionally, engineered bacteria capable of producing antiangiogenic peptides, such as Tum-5 (a fragment of tumstatin)^422^ or thrombospondin-1^423^, can suppress tumor growth in mouse models of hepatocellular carcinoma and melanoma by reducing tumor microvascular density.

Engineered bacteria have demonstrated diverse and innovative strategies for combating cancer beyond the direct destruction of tumor cells. For example, Canale et al. developed an engineered probiotic strain of *E. coli*, Nissle 1917, capable of colonizing tumors and metabolizing ammonia in the TME into L-arginine. This metabolic conversion strengthens the host’s defense mechanisms against cancer by enhancing the T-cell-mediated antitumor immune response.^424^ Similarly, Chowdhury et al. engineered a nonpathogenic *E. coli* strain to produce CD47 nanobodies that self-destruct in vivo. This mechanism facilitates the binding of nanobodies to CD47 proteins on cancer cells, disrupting their ability to evade immune surveillance and rendering them more vulnerable to immune-mediated destruction.^425^ Preclinical models have also shown promise with *S. typhimurium* engineered to secrete heterologous flagellar protein B derived from *Vibrio cholerae* or with attenuated *L. monocytogenes*. These engineered bacteria can repolarize M2 macrophages in the TME into proinflammatory M1 phenotypes, thereby promoting robust antitumor immunity.^426^ Furthermore, *Listeria* strains engineered to deliver galactosylceramide have been shown to increase natural killer T-cell activity and reduce metastasis in mouse breast tumor models.^427^ Zichun Hua’s team developed a novel therapeutic approach based on a dual-engineered macrophage‒microbe system for treating lung metastatic tumors. By integrating modified macrophages with engineered antitumor bacteria, this system could leverage the chemotactic and camouflaging properties of macrophages to enhance bacterial enrichment and biocompatibility within the tumor. The experimental results demonstrated that this therapy effectively halted the progression of lung metastatic tumors in three mouse tumor models. It activates the TME; suppresses protumor M2 macrophages, MDSCs, and Tregs; and enhances antitumor M1 macrophages, mature dendritic cells, and effector T cells. This study provides a new strategy for the targeted enhancement of antitumor immunotherapy.^428^ Another novel approach involves the use of bacterial enzymes to modify the tumor stroma, thereby overcoming the barriers posed by solid tumors resistant to conventional therapies. Ebert et al. engineered an attenuated, tumor-targeting strain of *S. typhimurium* to secrete functional bacterial hyaluronidase. This enzyme effectively degrades human hyaluronan deposits within the TME, significantly increasing the permeability of chemotherapeutic agents in orthotopic human PDAC mouse models. This work highlights the potential of engineered bacteria as safe and effective tools for enhancing cancer treatment and drug delivery.^429^

Probiotics and FMT as adjuncts have also been investigated in cancer therapy. The U.S. The Food and Drug Administration and the World Health Organization define probiotics as “live microorganisms that, when administered in adequate amounts, confer a health benefit on the host.” Probiotics have been shown to suppress colorectal cancer by modulating innate immune responses, promoting apoptosis, reducing oxidative stress, and improving the composition of the gut microbiota.^430^ The genus *Lactobacillus*, which has been recognized as the most commonly studied probiotic in clinical trials, can reduce the abundance of Enterobacteriaceae and modulate gut immune responses in patients with colorectal cancer. In contrast, Bifidobacterium longum has no such effects.^431^ Furthermore, *H. pylori* infections, often treated with amoxicillin, clarithromycin, and proton pump inhibitors, can significantly alter the indigenous gut microbiota to exert long-term impacts.^432^ A randomized controlled trial comparing conventional *H. pylori* therapy with and without probiotic cotreatment revealed more pronounced disruptions in the gut microbiome and a higher prevalence of antibiotic-resistant bacteria after conventional *H. pylori* treatment alone. Therefore, coadministration of probiotics could mitigate the adverse effects of *H. pylori* infection by preserving the gut microbial balance and enhancing host resilience.^433^

The efficacy and safety of probiotics remain a topic of ongoing debate. At present, further elaboration is needed to provide additional data for addressing critical issues such as colonization ability, therapeutic effectiveness, and long-term adverse effects. Most probiotic anticancer therapies are still in the preclinical stage. For example, a prior clinical trial surveyed the lifestyle, dietary habits, and health status of 306 breast cancer patients and 662 controls aged 40–55 years. A previous study revealed that the regular consumption of the probiotic *Lactobacillus casei* Shirota and soy isoflavones reduces the risk of breast cancer in Japanese adolescent females.^434^ Probiotics also show potential in preventing postoperative complications, mitigating treatment-associated toxicity, and improving quality of life in cancer patients. A 12-year cohort study involving 45,241 volunteers revealed that the regular intake of yogurt containing *Streptococcus thermophilus* and *Lactobacillus delbrueckii* subsp. *bulgaricus* was significantly associated with a reduced risk of colorectal cancer. Despite this promising evidence, there are still inadequate clinical studies in humans.^435^ For example, in a clinical study of patients with AML undergoing chemotherapy, supplementation with *Lactobacillus acidophilus* or *Saccharomyces boulardii* did not result in significant improvements in immune suppression. Alarmingly, probiotic supplementation in this cohort was associated with an increased incidence of systemic infections in supplemented patients.^436^

The most radical yet effective method for modulating the gut microbiome may be FMT, which is defined as the transfer of the entire gut microbiota from a donor, often a responder to ICIs, to the recipient. FMT preparations can be administered orally via freeze-dried capsules or directly through colonoscopy or gastroscopy. This approach has demonstrated remarkable efficacy in treating recurrent *C. difficile* infections by replacing the dysbiotic microbiota with healthy microbial communities.^437^ FMT can also alter the composition of fungal communities in the gut to mediate tumor treatment outcomes. For example, a high abundance of yeasts and *Aspergillus* in donor feces was associated with FMT efficacy, whereas an increased presence of *C. albicans* in donor feces would indicate reduced therapeutic success. Metabolic analyses further revealed a negative correlation of *Candida* abundance with total saturated fatty acids and a positive correlation with carbohydrate levels, whereas *Aspergillus* abundance showed an inverse relationship with recently ingested SCFAs. These metabolites may directly or indirectly modulate the therapeutic effect of FMT on tumors.^438^ Recently, two first-in-human clinical trials demonstrated that FMT from ICI-responsive melanoma patients to ICI-resistant melanoma patients reversed nonresponsiveness to ICIs in a subset of recipients.^384,439^ Partial remission in these trials might be associated with donor selection and successful engraftment of the donor microbiota in the recipient’s gastrointestinal tract. Even so, several clinical, regulatory, and scientific uncertainties remain, particularly regarding effective donor selection and long-term safety concerns. In 2019, two patients in separate clinical trials developed bacteremia caused by extended-spectrum beta-lactamase-producing *E. coli* following FMT from the same donor, resulting in the death of one patient.^440^ Because of this incident, the U.S. Food and Drug Administration issued a safety warning of FMT-associated infection risk. Therefore, regular screening of donor feces is essential for the strict restriction of the transmission of microorganisms that may cause adverse infectious events, especially for immunocompromised patients. To ensure successful ICI-FMT combination therapy, further clinical studies are crucial for better understanding the sources of donor FMT, the transplantation procedures, and the recipient phenotypes.

A significant challenge remains in the clinical application of engineered bacteria, primarily due to the considerable genetic and physiological disparities between model organisms and humans, as well as the inherent complexity of human diseases. The administration of live bacterial agents, unlike conventional small-molecule drugs and proteins, is further complicated by atypical pharmacokinetic profiles and nonlinear dose‒response dynamics, particularly challenging the determination of optimal dosing regimens. Furthermore, the use of live bacterial products carries the inherent risk of provoking infections or sepsis, especially for individuals with compromised immune function. Moreover, oncolytic bacterial or oncolytic virotherapy may convert tumor sites into localized, destructive infections,^411^ which, if mishandled, may result in severe infections or life-threatening consequences. Conventional aseptic testing and regulatory protocols are impractical given that live bacteria and bacterial spores are impervious to standard sterilization methods such as heat or filtration. While sterility cannot be guaranteed, stringent measures must be enacted to prevent the presence of other pathogens in the product. Crucially, stringent precautions are essential to prevent the dissemination of live bacteria into the clinical environment.

##### *Microbiota serving as vectors to aid in drug delivery*

Currently, two primary strategies are employed for utilizing microbes in tumor treatment. The first involves the use of live or dead bacteria to activate antitumor immune responses via specific antigens. The second leverages bacteria as carriers to deliver therapeutic agents, such as toxins, immunostimulants, and pharmaceutical compounds. Microorganisms, which are microscopic in size and have diverse functional capabilities, hold immense potential as programmable “robotic factories.” Advances in nanomaterials and bioengineering have enabled the genetic modification of bacteria to reduce toxicity while enhancing their precision in targeting tumor tissues.^441^ These modified bacteria can penetrate deep into necrotic and hypoxic regions of tumors to exert innate cytotoxic and immune-stimulating effects. Bacteria can also proliferate within cancerous tissues to continuously produce and maintain anticancer agents at therapeutic levels.^418,442^ Tumor-targeting bacteria also play a pivotal role in cancer therapy by serving as efficient payload delivery systems.^44^ Notably, microorganisms can be utilized as carriers to deliver toxins, immune stimulants, and pharmaceutical agents.^443,444^ In addition to exhibiting high precision in targeting tumor tissues, an optimal cancer therapy should also respond to external cues, regulate the host’s internal environment, and sustain therapeutic activity within the body. The success of therapeutic bacteria, whether they function autonomously or as living delivery vehicles, depends on achieving high targeting specificity and minimal toxicity. These bacteria can exert their respective therapeutic effects by penetrating tumors alongside cytokines, cytotoxic agents, regulatory molecules, prodrug-converting enzymes, or siRNAs. Generally, engineered bacteria are constructed by modifying natural bacterial strains by cutting-edge bioengineering techniques to meet specific therapeutic needs. These multifunctional and versatile microorganisms represent significant advancements in the development of highly effective and innovative cancer treatment modalities.^445^

Genetically modified bacteria tailored to specific therapeutic requirements can act as vehicles capable of infiltrating necrotic and hypoxic regions within tumor tissues. These bacteria can express cytokines; anticancer agents such as prodrug-transforming enzymes; immunomodulators in the form of DNA, RNA, or proteins; and exhibit inherent tumor-killing and immune-boosting properties. For example, Phan et al. recently engineered an attenuated strain of *S. typhimurium* carrying a plasmid that encodes a small hairpin RNA targeting indoleamine 2,3-dioxygenase. Treatment with this engineered strain significantly increased neutrophil infiltration into tumors and induced cancer cell death in a mouse model of colorectal cancer.^446^ Attenuated strains of *S. typhimurium* and their derivatives, which are tailored to transport anticancer agents, have demonstrated favorable tolerance in cancer patients and have been evaluated in early-phase clinical trials.^447^ Various genetically engineered bacterial strains capable of delivering prodrug-transforming enzymes (e.g., cytosine deaminase, herpes simplex virus type 1 thymidine kinase, β-glucuronidase, etc.) have shown efficacy in inhibiting tumor growth in preclinical models. These tumor-targeting bacteria can convert prodrugs such as 5-fluorocytosine, ganciclovir, and 9-aminocamptothecin glucuronide into their active chemotherapeutic forms (5-fluorouracil, ganciclovir-3-phosphate, and 9-aminocamptothecin, respectively), potentially increasing treatment specificity and minimizing systemic toxicity.^447,448^ Similarly, in mouse models, the genetically engineered *S. typhimurium* VNP20009 strain expressing the carboxypeptidase G2 has demonstrated anticancer effects when used in combination with substrate prodrugs.^449^ An innovative advancement in bacterial therapy involves engineering bacteria to respond to external stimuli, such as ultrasound waves. Upon injection into mice, these bacteria migrated to the tumor core, where ultrasound exposure activated a genetic switch that triggered the release of anticancer nanobodies.^450^ This strategy, which uses bacteria as carriers to deliver therapeutic payloads directly to tumor tissues during colonization, enables precise tumor eradication while preserving the microbiota.

In tumor therapy, the development of the engineered bacterial system N-V-J exemplifies a novel therapeutic approach. The VNP20009 strain, a facultative anaerobe *Salmonella* species, targets hypoxic regions of tumors. By surface-modifying VNP20009 with NHS-N782 heptamethine cyanine dye, researchers enhanced its binding to tumor cells, enabling photothermal therapy and imaging applications. Upon exposure to near-infrared light, the dye induced a photothermal effect that eradicated tumor cells directly. Additionally, the integration of JQ-1 derivatives into N-V-J suppressed PD-L1 expression within the TME, reducing immune suppression and augmenting T-cell-mediated antitumor responses. The inherent immunogenicity of this system, coupled with near-infrared-triggered tumor antigen release, elicited robust and sustained immune responses against both local and distant tumors.^451^ In addition to bacteria, certain viruses, such as parvoviruses and retroviruses, are also being explored as vectors for immunotherapy and cancer gene therapy. These viral vectors, which express antigens or cytokines locally, can restore tumor suppressor functions or enhance antitumor immune responses.^452^ In addition, these viruses can deliver genes encoding enzymes that convert inert prodrugs into active chemotherapeutic agents, expanding their potential utility in cancer treatment.^453^

#### Targeting the microbiota for cancer therapy

##### *Eradicating the microbiota for anticancer therapy*

Certain microorganisms can confer advantages to tumor cells, such as evading immune detection, promoting proliferation, inducing metastasis, and diminishing the efficacy of drug treatments. A significant challenge in tumor chemotherapy is drug resistance, which may develop owing to the presence of intratumoral bacteria that metabolize anticancer drugs. For example, Geller and colleagues showed that intratumoral bacteria expressing the bacterial enzyme cytidine deaminase might induce resistance by metabolizing gemcitabine, a nucleoside analog used in pancreatic cancer treatment, into an inactive form. Consequently, the eradication of relevant bacteria may indirectly enhance antitumor effects. Antibiotic treatments, such as the addition of ciprofloxacin,^371^ have been analyzed retrospectively to counteract this effect and improve survival rates in patients with PDAC.^454^ Similarly, Von Hoff et al. reported increased efficacy when antibiotics were used in combination with gemcitabine and nab-paclitaxel.^455^ In another study, actinomycin/neomycin was shown to reduce lung metastasis in B16 melanoma patients. Jin et al. reported that metronidazole administration reduced tumor growth in mouse models with KRAS mutations and TP53 deletions.^456^ In lung cancer and pancreatic cancer, treatment with antibiotics can transform an immunotolerant TME into an immunoactivated TME.^322,457^ Furthermore, Yu et al. reported that *F. nucleatum* was prevalent in colorectal cancer tissues from patients with recurrent disease after chemotherapy. In a colorectal cancer xenograft mouse model, *F. nucleatum* targeted TLR-4 and MyD88 to activate autophagy, leading to the selective loss of miR-18a* and miR-4802 expression, contributing to the occurrence of resistance to 5-fluorouracil.^373^ *F. In addition, nucleatum* can induce chemoresistance through other mechanisms, such as upregulating BIRC3 expression via the TLR-4/NF-kB pathway, which is significantly associated with 5-fluorouracil resistance in colorectal cancer patients.^36^ Therefore, regulating the microbiota with antibiotics may increase the effectiveness of anticancer treatments.

The systemic administration of antibiotics, while effective at inhibiting intratumoral bacterial growth, also disrupts the gut microbiota, leading to imbalances in other microbial communities within the body. These disturbances can adversely impact the efficacy of antitumor treatments, potentially reduce patient survival rates and increasing the risk of developing additional comorbidities. For example, in a prospective, multicenter cohort study, the use of broad-spectrum antibiotics prior to ICI therapy was associated with poorer overall survival and a higher incidence of refractory disease in patients with NSCLC, melanoma, and other tumors.^458^ Additionally, the use of antibiotics may be associated with reduced clinical benefits from ICIs in patients with renal cell carcinoma, NSCLC, or bladder cancer. A study indicated that exposure to antibiotics shortly before or after the start of ICIs might disrupt the microbiota and then cause damage to the gut microbiome, which might be detrimental to the efficacy of ICIs.^459^ Furthermore, Jing et al. reported that antibiotic treatment was associated with an increased risk of immune-related adverse events.^460^

The complex impact of antibiotics on the gut microbiota complicates the evaluation of their advantages and disadvantages in cancer treatment. Nonetheless, the clearance of the tumor-infiltrating microbiome frequently emerges as a critical aspect of cancer therapy. Consequently, methods for precisely targeting the tumor-infiltrating microbiome while minimizing disruption of the gut microbiota are essential. The use of cell membrane-permeable antibiotics, such as doxycycline, presents a potential strategy for achieving this balance. For example, Bingbing Wu et al. developed an endogenous proteo-ellagic acid nanocomplex that targets tumors via enhanced permeability and retention effects. This nanocomplex kills intratumoral bacteria to modulate the antitumor immune response.^389^ Hyunjun Choi et al. pioneered a novel approach in bacterial embolism therapy by developing an anaerobic bacteria metabolism-responsive carrier known as CA-BEM, which encapsulated Clostridium novyi-NT bacterial spores. This innovative design ensures the stability of bacterial spores during transport and facilitates their localized release within tumor tissues, thereby minimizing systemic side effects. The interaction between dipicolinic acid and Ca²⁺ in CA-BEM plays a pivotal role, as it allows rapid degradation upon the germination of C. novyi-NT, ensuring the efficient intratumoral release of vegetative cells. Once released, the metabolic products of bacteria, including oncolytic enzymes, can directly target and kill tumor cells while inducing immune responses, thereby significantly enhancing antitumor efficacy.^461^ Gao et al. developed metronidazole fluorouracil nanoparticles in a hydrophilic solution, achieving tumor-infiltrating microbiome targeting by increasing permeability and retention effects in solid tumors. To find solutions for effectively eliminating the tumor-promoting tumor-infiltrating microbiome while maintaining a balanced microbiota system, further in-depth analysis is needed to provide more insights into cancer treatment.^462^ The use of nanoantibiotics has been proposed to address the challenge of bacteria-infiltrating tumors. It involves the development of an amphiphilic small molecule, metronidazole‒fluorouracil, which self-assembles into metronidazole‒fluorouracil nanoparticles in a hydrophilic solution. Metronidazole, known for its broad-spectrum activity against anaerobic bacteria, is linked with fluorouracil. The disulfide bonds in the linker are designed to cleave in response to elevated glutathione levels characteristic of the TME. This mechanism facilitates the targeted delivery of metronidazole by nanoparticles, effectively targeting intratumoral bacteria while minimally affecting the homeostasis of the gut microbiota. The metronidazole‒fluorouracil nanoparticles exhibit a synergistic antitumor effect by concurrently targeting the intratumoral microbiome and tumor cells. This dual-targeting strategy offers significant clinical advantages, such as effective eradication of tumor-promoting bacteria and preservation of homeostatic microbiota.^462^ Another team is dedicated to exploring a drug delivery system that can effectively target anaerobic bacteria in hypoxic tumor sites with narrow-spectrum antibiotics to reduce damage to the intratumoral symbiotic microbiota.^463^ Tumor hypoxia is directly related to tumor acidosis via the Warburg effect, which is supported by the metabolism of pyruvate to lactate and ethanol by cancer cells.^464^ Menglin Wang et al. leveraged the low pH environment of hypoxic tumors, which results from elevated lactate levels, to facilitate drug release within anaerobic bacteria located in the tumor. In their study, liposomes loaded with metal and nitroimidazole antibiotic complexes were employed to target these anaerobic bacteria, thereby inhibiting tumor growth. Nitroimidazoles, a class of antimicrobial prodrugs, remain inactive before reduction by iron–oxygen reductase systems in obligate anaerobes. The nitro group is converted to an amine by an oxygen-insensitive nitroreductase, rendering the nitroimidazole nontoxic. The hypoxia-activation properties of nitroimidazoles specifically enable them to eliminate anaerobic bacteria in hypoxic tumors. Moreover, a dual-cascade-responsive drug delivery system, sNP@G/IR, was introduced in a previous study to address the challenges of limited drug penetration and potent bacteria-mediated drug inactivation in pancreatic cancer, which often lead to chemotherapy failure. To enhance anticancer efficacy, researchers have designed a nanoparticle system of sNP@G/IR, enabling the sequential stimulation of deep tumor penetration, elimination of intratumoral bacteria, and controlled release of chemotherapeutic agents. This system comprises a hyaluronic acid shell and a glutathione-responsive polymer core (NP@G/IR), encapsulating gemcitabine and a photothermal agent (IR 1048). The polymer core is tailored as an antibiotic substitute and optimized for antibacterial activity and selectivity. The hyaluronic acid shell targets CD44, enabling sNP@G/IR to home actively to the tumor site, where hyaluronidase in the extracellular matrix degrades the hyaluronic acid shell. This degradation results in a reduced size and charge-reversed NP@G/IR, facilitating deep tumor penetration. Upon cellular internalization, the guanidine exposed by NP@G/IR disrupts the cell membrane, effectively killing intracellular bacteria.^143^ To eliminate specific bacteria and improve patient survival rates, in addition to the use of antibiotics or their analogs, a novel dual-function antitumor strategy based on nitrogen-doped carbon nanodots has been proposed by some scholars. In this strategy, the carbon nanodots can act as nanoscale inhibitors of cisplatin to overcome bacterium-induced gemcitabine resistance.^465^ The incorporation of nitrogen into carbon materials can mimic the structure of porphyrins in natural peroxidases and endow the nanomaterials with various enzymatic activities, including superoxide dismutase, catalase, peroxidase, and oxidase activities.^466^ In addition to the biocatalytic functions of carbon materials, nitrogen-doped carbon nanodots can also competitively bind to the active center of cisplatin, thereby preventing intratumoral bacterium-induced gemcitabine metabolism. Furthermore, bacteriophages can precisely target and eliminate harmful intratumoral microbes, and several phages targeting Clostridium bacteria have been shown to effectively invade intratumoral bacteria.^75^ Scientists are now utilizing synthetic biology techniques to modify phages into programmable bacterial assassins capable of delivering an effective therapeutic load to attract antitumor immune cells to the attack site.^467^ For example, azide-modified phages that target *F. nucleatum* have been investigated for their ability to covalently bind irinotecan nanoparticles and enhance their delivery to mouse colon tumors.^468^ Bacteriophages may also induce host inflammation and immune responses, despite their reported safety owing to their reproduction in bacteria only.^469^ Therefore, further determination of the type, dosage, and mode of administration of bacteriophages is critical. Bacteriophages offer the advantage of specificity as custom-made bactericidal agents; however, their ability to target only particular bacterial strains is limited, thus limiting their therapeutic efficacy. Consequently, selecting and producing therapeutic phages tailored to the diverse strains encountered in different patients is essential. It is vital to expand the bactericidal spectrum of bacteriophages. Among the strategies explored, phage cocktail therapy, which involves a mixture of multiple bacteriophages, is currently regarded as the most promising approach.^470^ Nevertheless, bacteriophages, which cannot completely replace antibiotics, are best utilized as supplements to antibiotics or as a last resort when other effective treatments are unavailable.

### Clinical research progress

#### Pharmacological agents approved for clinical application

The clinical application of viruses for cancer treatment is still in its nascent stages within modern medicine. Progress has been made in this field despite the potential risks associated with the introduction of wild-type viruses, such as adverse outcomes due to viral replication in healthy tissues. Notably, in 2004, Rigvir, an oncolytic picornavirus with a degree of tumor selectivity, became the first oncolytic virotherapy approved for cancer treatment in Latvia and several other countries. This ECHO-7 virus represents a significant step forward in the development of viral therapies for oncology.^471^ Another oncolytic virus, named H101 (Oncorine), has been used in China since 2005 for solid tumor treatment. Moreover, several oncolytic viruses have been approved for clinical use and have demonstrated significant potential in cancer therapy. One example is Talimogene laherparepvec, a genetically engineered HSV expressing granulocyte‒macrophage colony‒stimulating factor. Intratumoral injection of Talimogene laherparepvec in patients with metastatic melanoma has been shown to promote T-cell infiltration and further enhance the efficacy of ICB therapies using anti-PD-1, anti-CTLA-4, etc.^414^ In addition to oncolytic viruses, certain microbial metabolites have also been approved for clinical use. p28 is a novel anticancer agent derived from azurin, a 128-amino-acid copper-containing ferredoxin secreted by the opportunistic pathogen *P. aeruginosa*. The helical motif of azurin and p28 allows preferential penetration into human cancer cells.^472,473^ As a cell-penetrating peptide, p28 is processed into the nucleus and disrupts the binding of constitutive photomorphogenesis protein 1 to p53. As an anticancer therapeutic, p28 has completed phase I clinical trials as an Investigational New Drug and has been approved by the U.S. Food and Drug Administration.^474^ Amino acid deprivation therapy utilizes enzymes secreted by microorganisms in anticancer treatments by exploiting the metabolic differences between cancerous and normal cells. Rapidly growing tumors often exhibit reduced expression of certain enzymes, leading to nutritional deficiencies in specific amino acids, making tumors susceptible to amino acid-depleting enzymes, which target them selectively. In contrast, normal cells are capable of synthesizing amino acids through their regular metabolic pathways, allowing amino acid depletion to specifically inhibit tumor growth without damaging healthy cells. Most of the enzymes employed in amino acid deprivation therapy are derived from microorganisms. Accordingly, a PEGylated form of this enzyme, marketed as Oncaspar™, was developed to mitigate the side effects associated with native *E. coli* asparaginase. This modified L-asparaginase, created by conjugating monomethoxy PEG to the enzyme, was approved by the U.S. Food and Drug Administration in 1994 and was incorporated into the first-line multidrug chemotherapy regimen for patients with acute lymphoblastic leukemia in 2006.^475^ A PEGylated form of Erwinia asparaginase is also under investigation. PEG-ADI has been confirmed in vivo to have an extended serum half-life and reduced immunogenicity.^476^ Recombinant Mycoplasma ADI-PEG 20, an “orphan drug” for the treatment of hepatocellular carcinoma, was approved by the U.S. Food and Drug Administration in 1999 and the European Medicines Evaluation Agency in 2005.^477^ Arginine deiminase and methioninase are undergoing clinical trials, and the therapeutic potential of lysine oxidase, glutaminase, and phenylalanine ammonia lyase is also being explored. Bacterial cancer therapy using attenuated live *Bacillus Calmette-Guérin* for immunotherapy in high-risk nonmuscle-invasive bladder cancer patients has also been approved for clinical use.^478^

#### Cancer microbiota in clinical trials

The impact of the microbiota on carcinogenesis has emerged as a significant field of research, characterized by increasing breadth and depth. The growing number of clinical trials, either ongoing or completed, underscores the efforts to promote the clinical application of microbial therapies. In the realm of cancer treatment research, current efforts are focused primarily on utilizing microbes to increase therapeutic efficacy. A notable approach involves the use of bacteria as vectors to boost immune responses. For example, *L. monocytogenes*, an intracellular pathogen, is capable of entering the cytoplasm of antigen-presenting cells through the hemolytic activity of listeriolysin O. This hemolytic activity disrupts the phagosome membrane, enabling the bacteria to escape into the cytoplasm. *L. monocytogenes*-induced infection serves as a classic model for inducing protective cellular immune responses, illustrating the potential of microbial-based strategies in cancer therapy.^479^ These bacteria have been widely used as vaccine vectors, with vaccines generating immune responses against breast cancer (4T1, MDA-MB-231) and other human tumors in phase I and II clinical trials. Certain microbes can also directly enhance antitumor immunity or therapeutic efficacy. For example, a clinical trial (NCT05032014) is evaluating whether the probiotic Probio-49 can augment the efficacy of PD-1 inhibitors in the treatment of liver cancer. CBM588, a bifidogenic live bacterial product, is hypothesized to enhance the response to checkpoint inhibitors through modulation of the gut microbiota. In a single-center study (NCT03829111), patients receiving combination therapy with nivolumab, ipilimumab, and CBM588 demonstrated significantly longer progression-free survival than those who did not receive CBM588. Although the findings did not reach statistical significance, the study still suggested that CBM588 may improve clinical outcomes in patients with metastatic renal cell carcinoma receiving nivolumab‒ipilimumab treatment^480^ (Table 2).

**Table 2:** Other clinical trials utilizing microbes and their derivatives for cancer therapy

NCT number	Official title	Cancer type	Intervention	Research purposes
NCT01975116	A Phase I Trial of p28 (NSC745104), a Non-HDM2 Mediated Peptide Inhibitor of p53 Ubiquitination in Pediatric Patients with Recurrent or Progressive CNS Tumors	Teratoid Tumor, Atypical Choroid Plexus Neoplasms, Anaplastic Astrocytoma	Drug: azurin-derived cell-penetrating peptide p28	This phase I trial studies the side effects and best dose of azurin-derived cell-penetrating peptide p28 (p28) in treating patients with recurrent or progressive central nervous system tumors.
NCT04167137	A Phase 1, Open-label, Multicenter Study of SYNB1891 Administered by Intratumoral Injection to Patients with Advanced/Metastatic Solid Tumors and Lymphoma Alone and in Combination with Atezolizumab	Metastatic Solid Neoplasm Lymphoma	Drug: SYNB1891 Drug: Atezolizumab	This Phase 1, open-label, multicenter, 2-arm study was designed to evaluate SYNB1891 when administered either as monotherapy (Arm 1) or in combination with atezolizumab (Arm 2) in participants with advanced/metastatic solid tumors or lymphoma. The primary objective was to evaluate the safety and tolerability of study treatment, with a secondary objective of assessing preliminary tumor response to treatment and exploratory objectives of evaluating the pharmacokinetics/pharmacodynamics (PK/PD) of study treatment.
NCT04601402	A Phase I/Ib Study to Evaluate the Safety, Tolerability, Biological and Clinical Activities of GEN-001 in Combination with Avelumab in Patients with Advanced Solid Tumors Who Have Progressed During or After Treatment with Anti-PD-(L)1 Therapy.	Solid Tumor Non-small cell lung cancer Squamous Cell Carcinoma of Head and Neck	Drug: GEN-001 Drug: Avelumab	This is a phase I/Ib, first-in-human (FIH), open-label, dose escalation and dose expansion study to evaluate the safety and tolerability, biological and clinical activities of GEN-001 in patients with locally advanced or metastatic solid tumors who have progressed on at least two lines of approved therapy for their histological subtypes which includes an anti-PD-1 or anti-PD-L1 based therapy (as mono or combination), when administered as combined with avelumab
NCT03775850	A Phase I Open-label Study of EDP1503 Alone and in Combination with Pembrolizumab in Patients with Advanced Metastatic Colorectal Carcinoma, Triple-negative Breast Cancer, and Checkpoint Inhibitor Relapsed Tumors	Colorectal Cancer Metastatic Triple Negative Breast Cancer Non-small cell lung cancer	Biological: EDP1503 Biological: Pembrolizumab	This study is being conducted to assess the safety, tolerability, and efficacy of EDP1503 alone and in combination with pembrolizumab in patients with advanced metastatic colorectal carcinoma, triple-negative breast cancer, and checkpoint inhibitor relapsed tumors
NCT03637803	A Phase I/II Open Label, Safety and Preliminary Efficacy Study of MRx0518 In Combination with Pembrolizumab in Patients with Advanced Malignancies Who Have Progressed On PD-1/PD-L1 Inhibitors.	Oncology Solid Tumor Non-small cell lung cancer	Drug: MRx0518 Drug: Pembrolizumab 25 MG/1 ML Intravenous Solution [KEYTRUDA]	A Phase I/II Open Label, Safety and Preliminary Efficacy Study of MRx0518 In Combination with Pembrolizumab in Patients with Advanced Malignancies Who Have Progressed On PD-1/PD-L1 Inhibitors
NCT03750071	VXM01 in combination with avelumab in n = 30 patients with progressive glioblastoma following standard treatment, with or without second surgery	Recurrent Glioblastoma	Biological: VXM01 Biological: Avelumab	An Open-label, Phase I/II Multicenter Clinical Trial of VXM01 in Combination with Avelumab in Patients with Progressive Glioblastoma Following Standard Treatment, With or Without Second Surgery
NCT03847519	A Phase 1/2, Open-Label Study of ADXS-503 Alone and in Combination with Pembrolizumab in Subjects with Metastatic Squamous or NSCLC	Lung Cancer, Non-Small Cell Metastatic Squamous Cell Carcinoma Metastatic Non-Squamous Cell Carcinoma	Drug: ADXS-503 Drug: Pembrolizumab	A Phase 1/2, Open-Label Study of ADXS-503 Alone and in Combination with Pembrolizumab in Subjects with Metastatic Squamous or Non-Squamous Non-Small Cell Lung Cancer
NCT02325557	A Phase 1-2 Dose-Escalation and Safety Study of ADXS31-142 Alone and of ADXS31-142 in Combination with Pembrolizumab (MK-3475) in Patients with Previously Treated Metastatic Castration-Resistant Prostate Cancer	Cancer Prostate Cancer	Drug: ADXS31-142 Drug: Pembrolizumab	A Phase 1/2 multicenter, dose determining, open-label study of ADXS31-142 monotherapy and a combination of ADXS31-142 and pembrolizumab (MK-3475) in participants with metastatic castration-resistant prostate cancer. Part A will be dose-determining part of ADXS31-142 monotherapy. Part B will be dose-determining part of ADXS31-142 and pembrolizumab (MK-3475) in combination. Part B expansion will treat additional participants with the recommended dose from Part B.
NCT02002182	Window of Opportunity Trial of Neoadjuvant ADXS 11-001 Vaccination Prior to Robot -Assisted Resection of HPV-Positive Oropharyngeal Squamous Cell Carcinoma	Head and Neck Cancer Squamous Cell Carcinoma of the Head and Neck HPV Positive Oropharyngeal Squamous Cell Carcinoma	Biological: ADXS11-001 (ADXS-HPV)	The purpose of this study is to see if an experimental vaccine, ADXS11-001, is effective in stimulating the body’s defense system against HPV-positive oropharyngeal squamous cell carcinoma before transoral (through the mouth) surgery. The experimental product ADXS11-001 uses a live strain of the *Listeria monocytogenes* (Lm) bacteria that has been genetically modified such that the risk of getting an infection is significantly reduced. Several research studies have already been conducted with ADXS11-001 in men and women with cancer. Thus far, approximately 722 doses of ADXS11-001 have been given to 290 patients with HPV associated cancers.
NCT01675765	A Phase 1B Study to Evaluate the Safety and Induction of Immune Response of CRS-207 in Combination with Pemetrexed and Cisplatin as Front-line Therapy in Adults with Malignant Pleural Mesothelioma	Malignant Pleural Mesothelioma	Biological: Immunotherapy plus chemotherapy Biological: Immunotherapy with cyclophosphamide plus chemotherapy	CRS-207 is a weakened (attenuated) form of *Listeria monocytogenes* that has been genetically modified to reduce its capacity to cause disease, while maintaining its ability to stimulate potent immune responses. CRS-207 has been engineered to elicit an immune response against the tumor-associated antigen mesothelin, which has been shown to be present at higher levels on certain tumor cells (such as mesothelioma) than on normal cells. Pemetrexed and cisplatin are the standard chemotherapy regimen to treat malignant pleural mesothelioma. This trial will evaluate whether giving CRS-207 cancer vaccine with chemotherapy will induce anti-tumor immune responses and/or objective tumor response.
NCT00623831	A Phase 1 Study of Mixed Bacteria Vaccine (MBV) in Patients with Tumors Expressing NY-ESO-1 Antigen.	Melanoma Sarcoma Gastrointestinal Stromal Tumor (GIST) 6 more	Biological: Mixed bacterial vaccine	The primary objective was to determine the safety profile of MBV in subjects with malignant tumors that expressed the NY-ESO-1 antigen and to identify the dose that induced the desired pyrogenic effect. Secondary objectives were to evaluate the immunological effects and tumor response of subjects following vaccination.
NCT03190265	A Randomized Phase 2 Study of the Safety, Efficacy, and Immune Response of CRS-207, Nivolumab, and Ipilimumab with or Without GVAX Pancreas Vaccine (With Cyclophosphamide) in Patients with Previously Treated Metastatic Pancreatic Adenocarcinoma	Pancreatic Cancer	Drug: Cyclophosphamide Drug: Nivolumab Drug: Ipilimumab	The purpose of this study is to study the safety and clinical activity of nivolumab and ipilimumab in combination with either sequential administration of CY/GVAX pancreas vaccine followed by CRS-207 (Arm A) or with administration of CRS-207 alone (Arm B) in patients with pancreatic cancer.
NCT00006254	A Phase I Trial of a Live, Genetically Modified Salmonella Typhimurium (VNP20009) for the Treatment of Cancer by Intravenous Administration	Unspecified Adult Solid Tumor, Protocol Specific	Biological: salmonella VNP20009	RATIONALE: Biological therapies such as VNP20009 use different ways to stimulate the immune system and stop cancer cells from growing. PURPOSE: Phase I trial to study the effectiveness of VNP20009 in treating patients who have advanced solid tumors.
NCT00004216	A Phase I Trial of a Live, Genetically Modified Salmonella Typhimurium (VNP20009) for the Treatment of Cancer by Intratumoral Injection	Unspecified Adult Solid Tumor, Protocol Specific	Biological: salmonella VNP20009 Drug: cefixime Drug: ceftriaxone sodium	PURPOSE: Phase I trial to study the effectiveness of VNP20009 in treating patients who have advanced or metastatic solid tumors that have not responded to previous therapy.
NCT00004988	A Phase I Trial of a Live, Genetically Modified Salmonella Typhimurium (VNP20009) for the Treatment of Cancer by Intravenous Administration	Cancer Neoplasm Neoplasm Metastasis	Drug: VNP20009	The study will examine the safety and toxicities of intravenously administering a genetically modified type of Salmonella bacteria (VNP20009) and its impact on tumor growth in advanced or metastatic cancer (cancer that has spread from the primary site).
NCT01383148	A Phase IIB/III Randomized, Double-blind, Placebo Controlled Study Comparing First Line Therapy with or Without TG4010 Immunotherapy Product in Patients with Stage IV NSCLC	Non-Small Cell Lung Carcinoma	Biological: TG4010 Drug: placebo	This is a Phase IIb/III randomized, double-blind, placebo-controlled study to compare the efficacy and safety of first-line therapy combined with TG4010 or placebo in stage IV NSCLC.
NCT01924689	Phase I Safety Study of Intratumoral Injection of Clostridium Novyi-NT Spores in Patients with Treatment-refractory Solid Tumor Malignancies	Solid Tumor Malignancies	Biological: Clostridium novyi-NT spores	This protocol will examine the safety of intratumoral administration of Clostridium Novyi-NT spores in patients with treatment-refractory solid tumor malignancies. This investigational study will measure anti-tumor activity of C. novyi-NT administered intratumoral in patients with treatment-refractory solid tumor malignancies.
NCT03435952	A Phase Ib Investigation of Pembrolizumab in Combination with Intratumoral Injection of Clostridium Novyi-NT in Patients with Treatment-Refractory Solid Tumors	Malignant Neoplasm of Breast Malignant Neoplasms of Digestive Organs Malignant Neoplasms of Eye Brain and Other Parts of Central Nervous System	Drug: Pembrolizumab Biological: Clostridium Novyi-NT Drug: Doxycycline	The goal of this clinical research study is to find the highest tolerable dose of one of these bacterial therapies (Clostridium novyi-NT spores) that can be given in combination with pembrolizumab to patients with advanced solid tumors. The safety of this drug will also be studied, as well as whether it can help to control the disease.
NCT03265080	A Phase 1 Dose-Escalation Study of ADXS-NEO Expressing Personalized Tumor Antigens, Alone and in Combination with Pembrolizumab in Subjects with Advanced or Metastatic Solid Tumors	Colon Cancer Metastatic Head and Neck Cancer Metastatic Metastatic Non-Small Cell Lung Cancer	Biological: ADXS-NEO Biological: Pembrolizumab	This is a Phase 1, open-label, multicenter study of ADXS-NEO administered alone and in combination with pembrolizumab in participants with select advanced or metastatic solid tumors.
NCT02386501	A Phase 1b Dose Escalation Study of ADXS31-164 in Subjects with HER2 Expressing Solid Tumors	HER2 Expressing Solid Tumors	Drug: ADXS31-164	This is a Phase 1b, multicenter, open-label, dose-escalation study designed to estimate the maximum tolerated dose (MTD) and determine the recommended Phase 2 dose (RP2D) of ADXS31-164. Once the RP2D has been selected, up to 4 expansion cohorts will be evaluated.
NCT02399813	Phase 2 Study of ADXS11-001 in Subjects with Persistent/Recurrent, Loco-Regional or Metastatic Squamous Cell Carcinoma of the Anorectal Canal	Anal Cancer Rectal Cancer	Drug: Axalimogene filolisbac	This is a single arm Phase 2 study. Stage 1 and 2 of the study are monotherapy evaluations of ADXS11-001 in 31 and 24 participants, respectively with persistent/recurrent, loco-regional or metastatic squamous cell carcinoma (SCCA) of the anorectal canal that have received at least 1 regimen for the treatment of advanced disease.
NCT05038150	Phase I/IIa, Open-label Study to Evaluate Safety, Tolerability and Preliminary Efficacy of Modified Salmonella Typhimurium SGN1 in Patients with Advanced Solid Tumor	Advanced Solid Tumor	Drug: SGN1	To assess the safety and tolerability followed by a dose expansion study to characterize safety, and preliminary efficacy of SGN1 in participants with refractory solid tumors.
NCT02718430	VXM01 Phase I Study in Patients with Metastatic Colorectal Cancer with Liver Metastasis Under Second or Third Line Therapy to Examine Safety, Efficacy, and Immune Biomarkers After Treatment with VXM01	Colorectal Cancer	Drug: VXM01	Phase I study in patients with metastatic colorectal cancer with liver metastasis under second or third line therapy to examine safety, efficacy, and immune biomarkers after treatment with VXM01
NCT01486329	VXM01 Phase I Dose Escalation Study in Patients with Locally Advanced, Inoperable and Stage IV Pancreatic Cancer to Examine Safety, Tolerability, and Immune Response to the Investigational VEGFR-2 DNA Vaccine VXM01	Stage IV Pancreatic Cancer	Biological: VXM01 Biological: Placebo	First-in-human phase I dose escalation study in patients with locally advanced, inoperable and stage IV pancreatic cancer to examine safety, tolerability, and immune response to the investigational VEGFR-2 DNA vaccine VXM01 to examine safety and tolerability, clinical and immunogenic response to the investigational vascular endothelial growth factor receptor 2 (VEGFR-2) DNA vaccine VXM01, and to define the maximum tolerated dose.
NCT02718443	VXM01 Phase I Pilot Study in Patients with Operable Recurrence of a Glioblastoma to Examine Safety, Tolerability, Immune and Biomarker Response to the Investigational VEGFR-2 DNA Vaccine VXM01	Glioblastoma	Drug: VXM01	VXM01 phase I pilot study in patients with operable recurrence of a glioblastoma to examine safety, tolerability, immune and biomarker response to the investigational VEGFR-2 DNA vaccine VXM01
NCT03189030	A Phase 1 Safety and Tolerability Study of Personalized Live, Attenuated, Double-Deleted Listeria Monocytogenes Immunotherapy in Adults with Metastatic Colorectal Cancer	Colorectal Neoplasms	Biological: Personalized Live, Attenuated, Double-Deleted Listeria Monocytogenes	This study will evaluate the safety and tolerability of a personalized live, attenuated, double-deleted *Listeria monocytogenes* treatment in adults with metastatic colorectal cancer.
NCT02243371	A Randomized Phase 2 Study of the Safety, Efficacy, and Immune Response of GVAX Pancreas Vaccine (With Cyclophosphamide) and CRS-207 With or Without Nivolumab in Patients with Previously Treated Metastatic Pancreatic Adenocarcinoma	Previously Treated Metastatic Adenocarcinoma of the Pancreas	Biological: CRS-207 Biological: CRS-207 Drug: nivolumab	The primary objective of this study is to compare the overall survival of subjects with previously treated metastatic pancreatic cancer treated with cyclophosphamide (CY)/nivolumab/GVAX pancreas vaccine followed by nivolumab/CRS-207 (Arm A) to subjects treated with CY/GVAX pancreas vaccine followed by CRS-207 (Arm B).
NCT02004262	A Phase 2B, Randomized, Controlled, Multicenter, Open-Label Study of the Efficacy and Immune Response of GVAX Pancreas Vaccine (With Cyclophosphamide) and CRS 207 Compared to Chemotherapy or to CRS-207 Alone in Adults with Previously Treated Metastatic Pancreatic Adenocarcinoma	2nd-line, 3rd-line and Greater Metastatic Pancreatic Cancer	Biological: GVAX Pancreas Vaccine Biological: CRS-207 Drug: Chemotherapy	Test the safety, immune response and efficacy of GVAX pancreas vaccine (with cyclophosphamide) and CRS-207 compared to chemotherapy or CRS-207 alone in adults with previously treated metastatic pancreatic adenocarcinoma
NCT05014776	A Phase 2 Study of the Safety, Efficacy, and Immune Response of CRS-207, Pembrolizumab, Ipilimumab, and Tadalafil in Patients with Previously Treated Metastatic Pancreatic Adenocarcinoma	Pancreatic Cancer	Drug: Tadalafil Drug: Pembrolizumab Drug: Ipilimumab	The purpose of this study is to evaluate the safety and clinical activity of tadalafil, pembrolizumab, ipilimumab, and CRS-207 in subjects with metastatic pancreatic adenocarcinoma who have progressed after at least 1 prior chemotherapy regimen.
NCT01417000	A Phase 2, Randomized, Multicenter, Open-Label Study of the Efficacy and Immune Response of the Sequential Administration of GVAX Pancreas Vaccine Alone or Followed by CRS-207 in Adults with Metastatic Pancreatic Adenocarcinoma	Metastatic Pancreatic Cancer	Biological: GVAX Pancreas Biological: CRS-207 Drug: Cyclophosphamide	Test the safety, immune response and efficacy of GVAX pancreas vaccine (with cyclophosphamide) and CRS-207 compared to GVAX pancreas vaccine (with cyclophosphamide) alone in adults who have failed or refused prior treatment for metastatic pancreatic cancer.
NCT06563947	A Clinical Study to Evaluate the Effectiveness of Oral Enterobacterial Capsules in Patients With Intermediate and Advanced HCC Who Have Progressed After Immune Checkpoint Inhibitor Combination With Anti-Angiogenesis Targeted Agents	Liver Cancer	Biological: Oral enterobacterium capsules	To evaluate the efficacy and safety of oral enterobacterial capsules in patients with intermediate and advanced HCC who have progressed after treating with immune checkpoint inhibitors in combination with anti-angiogenesis targeted agents.
NCT06563934	A Randomized Controlled Trial Evaluating the Effects of Oral Enterobacterial Capsules in Liver Cancer Patients Treated With Tyrosine Kinase Inhibitors in Combination with Immune Checkpoint Inhibitors (ICIs)	Liver Cancer	Biological: Oral enterobacterium capsules Drug: Lenvatinib + PD-1 monoclonal antibody Biological: Oral enterobacterium capsules placebo	To evaluate the additional efficacy and safety of oral enterobacterial capsules in patients with intermediate and advanced liver cancer and treated with tyrosine kinase inhibitors combined with immunotherapy.
NCT06448572	EXL01 in Combination with Nivolumab for Advanced NSCLC Refractory to Immunotherapy	Non-Small Cell Lung Cancer	Drug: EXL01	As treatment options are limited following progression on anti-PD-(L)1 and platinum-based chemotherapy, we propose this trial for patients who have failed to respond or have shown intolerance to standard therapies or for whom no appropriate therapies are known to provide clinical benefit. Considering the strong therapeutic rationale of an association between antineoplastic immunotherapy and EXL01 (single-strain of *F. prausnitzii*, a bacterium which is a dominant member of the healthy gut microbiota), we propose to assess this combination for NSCLC treatment. This is a pilot, Phase I/II, one-arm, monocentric study evaluating the combination of EXL01 with nivolumab treatment for Non-Small Cell Lung Cancer patients.

Numerous studies have investigated the potential of oncolytic viruses in cancer treatment (Table 3). Among these, DNX-2401 (or Delta-24-RGD) is a notable adenovirus engineered for selective replication in cells lacking the retinoblastoma protein. In a phase I clinical trial, DNX-2401 exhibited promising results, as favorable clinical responses were observed in 20% of patients with recurrent glioblastoma who were treated via intratumoral injection.^481^ In a phase II clinical trial, compared with ipilimumab monotherapy, Talimogene laherparepvec plus ipilimumab significantly increased the response rate of patients with metastatic melanoma.^482^ Recently, several studies have indicated that FMT can enhance the antitumor effects of ICIs and overcome resistance to immunotherapy^483^ (Table 4). In a phase I clinical trial (NCT03353402), FMT followed by reinduction of anti-PD-1 therapy was adopted for 10 melanoma patients who were previously unresponsive to PD-1 blockade. Three of the 10 patients exhibited tumor shrinkage, including two with a partial response and one with a complete response.^439^ Several studies have also investigated the depletion of specific pathogens alongside the utilization of beneficial microbes. For example, a clinical trial (NCT04660123) administered a quadruple therapy that included colloidal bismuth subcitrate to patients with gastric cancer to eradicate *H. pylori* while examining the incidence of adverse reactions and improvements in symptoms (Table 5). Additionally, the potential of itraconazole for treating various types of cancer has been explored in both preclinical experiments and clinical trials (NCT02749513).^29^ The anticipated outcomes of these studies may further promote the clinical application of microbiota-related therapies, although additional research is still needed to elucidate their precise underlying mechanisms.

**Table 3:** Clinical trials of cancer therapy utilizing oncolytic bacteria or oncolytic viruses

NCT number	Official title	Cancer type	Intervention	Research purposes
NCT00805376	Phase I Trial of Conditionally Replication-Competent Adenovirus (DNX-2401, Formerly Known as Delta-24-RGD-4C) for Recurrent Malignant Gliomas	Brain Cancer, Central Nervous System Diseases	Drug: DNX-2401 Procedure: Tumor Removal	The goal of this clinical research study is to find the highest tolerable dose of DNX-2401 that can be injected directly into brain tumors and into the surrounding brain tissue where tumor cells can multiply. A second goal is to study how the new drug DNX-2401 affects brain tumor cells and the body in general.
NCT03294486	Safety and Efficacy of the oncolytic virus Armed for Local Chemotherapy, TG6002/5-fluorocytosine, in Recurrent Glioblastoma Patients	Glioblastoma Brain Cancer	Drug: Combination of TG6002 and 5-flucytosine	TG6002/Flucytosine appears as a very promising therapeutic strategy in glioblastoma patients who merits consideration for early phase clinical trial.
NCT02798406	A Phase II, Multicenter, Open-label Study of a Conditionally Replicative Adenovirus (DNX-2401) With Pembrolizumab (KEYTRUDA®) for Recurrent Glioblastoma or Gliosarcoma (CAPTIVE/KEYNOTE-192)	Brain Cancer, Brain Neoplasm, Glioblastoma, Glioma	Biological: DNX-2401 Biological: pembrolizumab	The purpose of this Phase II study is to evaluate how well a recurrent glioblastoma or gliosarcoma tumor responds to one injection of DNX-2401, a genetically modified oncolytic adenovirus, when delivered directly into the tumor followed by the administration of intravenous pembrolizumab (an immune checkpoint inhibitor) given every 3 weeks for up to 2 years or until disease progression.
NCT02625857	An Open-Label, Phase 1 Study of the Safety and Immunogenicity of JNJ-64041809, a Live Attenuated Listeria Monocytogenes Immunotherapy, in Subjects With Metastatic Castration-resistant Prostate Cancer	Prostatic Neoplasms, Castration-Resistant	Biological: JNJ-64041809 (Cohort 1 A and 1B) Biological: JNJ-64041809 (Cohort 2 A and 2B)	The purpose of this study is to find and evaluate the recommended Phase 2 dose (RP2D) of JNJ-64041809, a live attenuated double deleted (LADD) *Listeria monocytogenes* (bacteria in which two virulence genes, which encode molecules that help cause disease, have been removed) when administered intravenously to participants with metastatic castration-resistant prostate cancer.
NCT01967758	Phase I Study of Safety and Immunogenicity of ADU-623, a Live-attenuated Listeria Monocytogenes Strain (ΔactA/ΔinlB) Expressing the EGFRvIII-NY-ESO-1 Vaccine, in Patients With Treated and Recurrent WHO Grade III/IV Astrocytomas	Astrocytic Tumors Glioblastoma Multiforme Anaplastic Astrocytoma	Biological: Cohort 1 Biological: Cohort 2 Biological: Cohort 3	This Phase I clinical trial will examine the safety, tolerability and immunogenicity of a novel vaccine approach using a live-attenuated strain of *Listeria monocytogenes* expressing EGFRvIII and NY-ESO-1 antigens to induce proliferation of memory and effector T cells with the overall goal of promoting an immune response against high-grade astrocytic tumors.
NCT03206073	A Phase I/II Study of Pexa-Vec Oncolytic Virus in Combination With Immune Checkpoint Inhibition in Refractory Colorectal Cancer	Colorectal Cancer Colorectal Carcinoma Colorectal Adenocarcinoma Refractory Cancer Colorectal Neoplasms	Drug: Durvalumab Drug: Tremelimumab Biological: Pexa-Vec Biological: Pexa-Vec	To determine the safety, tolerability and feasibility of Pexa-Vec oncolytic virus in combination with immune checkpoint inhibition in patients with refractory metastatic colorectal cancer.
NCT03004183	Phase II Window of Opportunity Trial of Stereotactic Body Radiation Therapy and In Situ Oncolytic Virus Therapy in Metastatic Triple Negative Breast Cancer and Metastatic Non-Small Cell Lung Cancer Followed by Pembrolizumab	Metastatic Non-small Cell Lung Cancer; Metastatic Triple-negative Breast Cancer;	Biological: ADV/HSV-tk Drug: Valacyclovir Radiation: SBRT Drug: Pembrolizumab	This is a Phase II trial to determine the efficacy and safety of stereotactic body radiation therapy (SBRT) and in situ oncolytic virus therapy used as a window of opportunity treatment before pembrolizumab in patients with metastatic triple-negative breast cancer and metastatic NSCLC.
NCT03896568	Phase I Clinical Trial of Allogeneic Bone Marrow Human Mesenchymal Stem Cells Loaded with A Tumor Selective Oncolytic Adenovirus, DNX-2401, Administered Via Intra-Arterial Injection in Patients with Recurrent High-Grade Glioma	IDH1 wt Allele Recurrent Anaplastic Astrocytoma Recurrent Glioblastoma Recurrent Gliosarcoma Recurrent Malignant Glioma	Biological: Oncolytic Adenovirus Ad5-DNX-2401 Procedure: Therapeutic Conventional Surgery	This phase I trial studies best dose and side effects of oncolytic adenovirus DNX-2401 in treating patients with high-grade glioma that has come back (recurrent). Oncolytic adenovirus DNX-2401 is made from the common cold virus that has been changed in the laboratory to make it less likely to cause an infection (such as a cold). The virus is also changed to target brain cancer cells and attack them.
NCT0325942	Phase II Neoadjuvant Trial of Nivolumab in Combination With HF10 Oncolytic Viral Therapy in Resectable Stage IIIB, IIIC, IVM1a Melanoma (Neo-NivoHF10)	Melanoma	Drug: Nivolumab Drug: HF10	This is a single-arm, open label, Phase II study evaluating the safety and efficacy of neoadjuvant Nivolumab and HF10 in resectable stage IIIB, IIIC, and IVM1a melanoma.
NCT03714334	Phase I Trial of DNX-2440 Oncolytic Adenovirus in Patients With Recurrent Glioblastoma	Glioblastoma Glioblastoma, Adult	Drug: DNX-2440 injection	Patients with first or second recurrence of GBM will be treated with stereotactic injection of the oncolytic virus DNX-2440
NCT03866525	Phase I/II Study of OH2 Injection, an Oncolytic Type 2 Herpes Simplex Virus Expressing Granulocyte Macrophage Colony-Stimulating Factor, in Malignant Solid Tumors	Solid Tumor Gastrointestinal Cancer	Biological: OH2 injection, with or without irinotecan or HX008	This phase I/II study evaluates the safety and efficacy of OH2 as single agent or in combination with HX008, an anti-PD-1 antibody, in patients with malignant solid tumors (gastrointestinal cancers, head and neck cancers, soft tissue sarcomas).
NCT03225989	Phase I/II Trial Investigating an Immunostimulatory Oncolytic Adenovirus for Cancer	Pancreatic Adenocarcinoma Ovarian Cancer Biliary Carcinoma Colorectal Cancer	Drug: LOAd703	This Phase I/II trial evaluates LOAd703 in patients with cancer (pancreatic, biliary, colorectal or ovarian) together with their standard of care chemotherapy or using gemcitabine immune-conditioning. LOAd703 is administered by intratumoral image-guided injections.

**Table 4:** Clinical trials of cancer therapy utilizing probiotics and fecal microbiota transplantation

NCT number	Official title	Cancer type	Intervention	Research purposes
NCT03829111	Pilot Study to Evaluate the Biologic Effect of CBM588 in Combination With Nivolumab/Ipilimumab for Patients With Metastatic Renal Cell Carcinoma	Advanced Renal Cell Carcinoma, Clear Cell Renal Cell Carcinoma, Metastatic Renal Cell Carcinoma	Drug: Clostridium butyricum CBM 588 Probiotic Strain Biological: Ipilimumab Biological: Nivolumab	To determine the effect of *Clostridium butyricum* CBM 588 probiotic strain (CBM588) (in combination with nivolumab/ipilimumab) on the gut microbiome in patients with metastatic renal cell carcinoma.
NCT03358511	Engineering Gut Microbiome to Target Breast Cancer	Breast Cancer	Dietary Supplement: Probiotic	The purpose of this study is to determine if using probiotics will help the body’s immune system react to breast cancer. New studies showed that diverse species of bacteria inside the bowel might help improve immune system, particularly the ability of immune system to recognize cancer. This study will investigate how probiotics will affect the subjects’ immune system on breast cancer.
NCT03072641	Using Probiotics to Reactivate Tumor Suppressor Genes in Colon Cancer	Colon Cancer	Dietary Supplement: ProBion Clinica	The purpose of the study is to determine if probiotic bacteria have a beneficial effect on the colon cancer-associated microbiota and epigenetic alterations in colon cancer. Dietary supplementation consists of two ProBion Clinica tablets, yielding a daily dose of 1.4 ×10 ˄ 10 Bifidobacterium lactis Bl-04 (ATCC SD5219), 7×10 ˄ 9 *Lactobacillus acidophilus* NCFM (ATCC 700396), and 0.63 g inulin.
NCT03290651	Resetting the Breast Microbiome to Lower Inflammation and Risk of Cancer.	Breast Cancer Female	Dietary Supplement: Probiotic Natural Health Product - RepHresh Pro-B Dietary Supplement: Placebo	The researchers would like to test their theory that taking probiotic lactobacilli by mouth can lead to these organisms reaching the breast tissue and helping displace the harmful bacteria and reducing inflammation. A study in Spain has shown that oral intake of probiotic lactobacilli can not only cure mastitis, but lead to the lactobacilli reaching the milk glands. Women who breast feed for more than six months (and thereby pass along lactic acid bacteria to the infant from the breast milk) have a reduced risk of breast cancer.
NCT01562626	A Phase I/II Safety, Pharmacokinetic, and Pharmacodynamic Study of APS001F With Flucytosine and Maltose for the Treatment of Advanced and/or Metastatic Solid Tumors	Tumors Neoplasms Cancer	Drug: APS001F Drug: 5-fluorocytosine Drug: 10% maltose	The purpose of this study is to test the safety and efficacy of an investigational drug called APS001F when given with 5-fluorocytosine for treatment of solid tumors
NCT05032027	A Randomized Controlled Clinical Study of Oral Probiotics on Radiation Enteritis Stage II Induced by Pelvic Concurrent Chemoradiotherapy	Pelvic Cancer, Enteritis, Probiotics, Chemoradiotherapy	Other: probiotics	Effect of Probiotics on Raditon Enteritis in Pelvic Tumor Patients Receiving Radiotherapy.
NCT03817125	A Multicenter Phase 1b Randomized, Placebo-controlled, Blinded Study to Evaluate the Safety, Tolerability and Efficacy of Microbiome Study Intervention Administration in Combination With Anti-PD-1 Therapy in Adult Patients With Unresectable or Metastatic Melanoma	Metastatic Melanoma	Drug: Placebo for antibiotic Drug: Vancomycin pretreatment Drug: Nivolumab	This study is designed to evaluate the safety and tolerability of treatment with oral microbiome study intervention (SER-401) or matching placebo in combination with anti-PD-1 therapy (nivolumab) in participants with unresectable or metastatic melanoma.
NCT05032014	Probiotics Enhance the Treatment of PD-1 Inhibitors in Patients With Liver Cancer	Liver Cancer	Other: M9 Other: placebo	Previous study showed that the abundance of beneficial bacteria such as lactic acid bacteria, bifidobacteria and Akkermansia Muciniphila was significantly correlated with pD-1 inhibitor response, and regulating the intestinal flora content could improve the effect of PD-1 inhibitor on mouse tumors, indicating that microbial flora was involved in regulating cancer immunotherapy.
NCT05865730	A Phase 1/2 Study of Oncobax®-AK Administered in Combination with Immunotherapy to Patients with Advanced Solid Tumors	Carcinoma, Renal Cell Carcinoma, Non-Small-Cell Lung	Other: Live Bacterial Product - Akkermansia muciniphila	In the clinical setting, it is therefore hypothesized that the oral administration of Oncobax®-AK to cancer patients under immunotherapy, but whose gut microbiota is deficient in Akkermansia will restore/improve the efficacy of immunotherapy in patients with NSCLC or renal cell carcinoma.
NCT03686202	This study is designed to assess the safety, tolerability and engraftment (cumulative relative abundance) of MET-4 strains when given in combination with ICIs.	All Solid Tumors	Biological: MET-4	This study is designed to assess the safety, tolerability and engraftment (cumulative relative abundance) of MET-4 strains when given in combination with ICIs.
NCT04208958	Phase 1 Study of VE800 and Nivolumab in Patients with Selected Types of Advanced or Metastatic Cancer	Metastatic Cancer Melanoma Gastric Cance	Biological: VE800 Drug: Nivolumab Drug: Vancomycin Oral Capsule	This study evaluated the safety and efficacy of VE800 in combination with nivolumab in patients with selected types of advanced or metastatic cancer
NCT06904573	A Multicenter, Randomized Controlled Phase II Study of Evaluating the Efficacy and Safety of Immunotherapy Combined with Oral Probiotics Compound (Biolosion) in Patients with Advanced Urothelial Carcinoma	Advanced Urothelial Carcinoma	Drug: Probiotics Compound (Biolosion) Drug: Nab-paclitaxel Drug: Cisplatin	This is a multicenter, randomized, controlled phase II Study of evaluating the efficacy and safety of immunotherapy combined with probiotics compound (Biolosion) in patients with advanced urothelial carcinoma.
NCT06186089	Effects of Total Gastrectomy or Double Track Reconstruction on Gut Microbiome and Cognitive Function in Patients with Proximal Gastric Cancer	Perioperative Neurocognitive Disorders Gastric Cancer	Dietary Supplement: probiotics	In this study, the investigators will compare the differences of gut microbiota between total gastrectomy and double-tract reconstruction, to investigate the effect of gastric acid on the gut microbiota colonizing, and the effect of different surgical procedures on the postoperative cognitive function of proximal gastric cancer patients.
NCT06245486	Study on the Efficacy of Crispact® (Probiotic Containing Lactobacillus Crispatus M247) in the Sterilization of HPV-HR and in the Reconstitution of the Normal Vaginal Microbiota	Human Papillomavirus Infection	Dietary Supplement: Crispact® Dietary Supplement: Placebo	There is growing scientific interest in probiotic supplementation as a possible therapy for clearing the human papillomavirus infection and reducing the risk of development of cervical cancer.
NCT06428422	The Impact of Bifidobacterium Lactis Supplementation on Survival and Treatment Response in Metastatic Non-small Cell Lung Cancer Patients Receiving Immunotherapy (Nivolumab)	Metastatic Non-small Cell Lung Cancer	Dietary Supplement: *Bifidobacterium animalis* subsp. lactis Bl-04 Other: Placebo	The aim of this study is to evaluate the effect of a probiotic supplement containing *Bifidobacterium animalis* lactis BL-04 on the clinical effectiveness of immunotherapy in patients diagnosed with metastatic non-small cell lung cancer who are receiving immunotherapy.
NCT06039644	To Evaluate the Efficacy of Probiotics in Improvement and Prevention of Chemotherapy Associated Side Effects in Patients with the Breast Cancer	Breast Cancer	Dietary Supplement: Probiotic Other: Placebo	Chemotherapy-associated side-effects would affect therapeutic effect, quality of life, and cause permanent harm to breast cancer patients. This study is designed to explore after consumption of probiotics of lactobacillus composite strain powder sachets for 6 months in breast cancer chemotherapy, and whether the improvement of meliorate the side effects, further assists patients completing the chemotherapy.
NCT06865521	Safety and Efficacy of Akkermansia Probiotics Combined with Anti-PD-1 Monoclonal Antibody in MSS/pMMR Advanced Colorectal Cancer	Colorectal Cancer	Dietary Supplement: Akkermansia Probiotics	The investigators propose to conduct a single-center, single-arm, Phase I clinical study to explore the safety and efficacy of Akkermansia probiotics combined with anti-PD-1 monoclonal antibody in patients with MSS/pMMR advanced colorectal cancer, as well as its impact on gut microbiota and the immune microenvironment.
NCT03353402	Altering the Gut Microbiota of Melanoma Patients Who Failed Immunotherapy Using FMT From Responding Patients	Melanoma Stage Iv Unresectable Stage III Melanoma	Procedure: FMT	Altering the Gut Microbiota of Melanoma Patients Who Failed Immunotherapy Using FMT From Responding Patients.
NCT03772899	Fecal Microbial Transplantation in Combination with Immunotherapy in Melanoma Patients	Melanoma	Drug: Fecal Microbial Transplantation	The investigators will combine FMT with the approved immunotherapy drugs pembrolizumab or nivolumab that are the standard of care for the treatment of advanced melanoma. The purpose of this study is to examine the safety of combining these two therapies in melanoma patients.
NCT04521075	A Phase Ib Trial to Evaluate the Safety and Efficacy of FMT in Combination With Nivolumab in Subjects with Metastatic or Inoperable Melanoma, Microsatellite Instability-high or Mismatch-repair Deficient Cancer, or NSCLC	Melanoma Stage IV Unresectable Melanoma NSCLC Stage IV	Biological: Fecal Microbial Transplantation by capsules	A Phase Ib trial to evaluate the safety and efficacy of FMT in combination with Nivolumab in subjects with metastatic or inoperable melanoma, microsatellite instability-high or Mismatch-repair Deficient Cancer, or NSCLC
NCT04924374	Microbiota Transplant in Advanced Lung Cancer Treated with Immunotherapy	Lung Cancer	Dietary Supplement: Microbiota Transplant plus anti PD1 therapy Drug: anti PD1 therapy	The gut microbiota can modulate the effectiveness of cancer therapies, especially immunotherapy. Manipulating the microbial populations in patients with advanced lung cancer through fecal microbiota transplantation from healthy individuals or from long-term survivors to advanced lung cancer will enhance the efficacy of immunotherapy.
NCT04951583	Phase II Trial of Fecal Microbial Transplantation in Patients with Advanced Non-Small Cell Lung Cancer and Melanoma Treated with ICIs.	Non-small cell lung Cancer Metastatic Advanced Melanoma	Combination Product: FMT + ICI	The aim of this study is to assess the anti-tumor activity of FMT administered in combination with ICI therapy.
NCT03819296	Role of Microbiome in the Realm of Immune-Checkpoint Inhibitor Induced GI Complications In Cancer Population	Cutaneous Melanoma AJCC v8	Other: Best Practice Other: Biospecimen Collection Procedure: Endoscopic Procedure	This trial studies the role of the gut microbiome and effectiveness of a fecal transplant on medication-induced gastrointestinal (GI) complications in patients with melanoma or genitourinary cancer.
NCT04116775	A Phase II Single Arm Study of FMT in Men with Metastatic Castration Resistant Prostate Cancer Whose Cancer Has Not Responded to Enzalutamide + Pembrolizumab	Prostate Cancer Prostate Cancer Metastatic	Biological: Fecal microbiota transplant Drug: Pembrolizumab Drug: Enzalutamide	To determine the anticancer effect of fecal microbiota transplant from participants who respond to pembrolizumab into those who have not responded in metastatic castration resistant prostate cancer.
NCT03341143	Phase II Feasibility Study of FMT in Advanced Melanoma Patients Not Responding to PD-1 Blockade	Melanoma	Drug: Fecal Microbiota Transplant with Pembrolizumab	The main goal of this research study is to determine if the FMT improves the body’s ability to fight your cancer.
NCT04038619	FMT for Immune-Checkpoint Inhibitor Induced-Diarrhea/Colitis in Genitourinary Cancer Patients	Colitis Diarrhea Malignant Genitourinary System Neoplasm	Procedure: Fecal Microbiota Transplantation Drug: Loperamide	This trial studies how well fecal microbiota transplantation works in treating diarrhea or colitis (inflammation of the intestines) that is caused by certain types of medications (called immune-checkpoint inhibitors) in patients with genitourinary cancer.
NCT04721041	Efficacy and Safety of Washed Microbiota Transplantation for The Treatment of Oncotherapy-Related Intestinal Complications	Intestinal Complications Cancer	Procedure: Washed Microbiota Transplantation (WMT)	This study aimed to exploring the therapeutic potential of WMT in the treatment of oncotherapy-related intestinal complications and improving the quality of life of patients.
NCT04163289	Preventing Immune-Related Adverse Events in Renal Cell Carcinoma Patients Treated with Combination Immunotherapy Using Fecal Microbiota Transplantation	Renal Cell Carcinoma	Drug: Fecal Microbiota Transplantation	The goal of this project is to study the safety of such FMT combination treatment and reduce occurrence of immune-related toxicities in patients, allowing them to continue their cancer treatments in the hopes of a better outcome. The investigators will also be looking at changes in the immune populations, microbiome profile of patients, response to treatment, and patient survival as secondary objectives.
NCT04988841	Prospective Randomized Clinical Trial Assessing the Tolerance and Clinical Benefit of Fecal Transplantation in Patients with Melanoma Treated with CTLA-4 and PD-1 Inhibitors	Melanoma	Drug: MaaT013 Drug: Ipilimumab Drug: Nivolumab	This trial is about assessing the tolerance and clinical benefit of fecal microbiome transfer in patients with melanoma in addition to the usual treatment with immunotherapy combining ipilimumab (CTLA-4 inhibitor) and nivolumab (PD-1 inhibitor).
NCT06205862	Efficacy and Safety of Fecal Microbiota Transplantation in Reducing Recurrence of Colorectal Adenomas After Endoscopic Resection: a Multicenter, Open-label, Randomized Controlled Study	Colorectal Adenoma	Procedure: fecal microbiota transplantation	The goal of this clinical trial is to learn about the efficacy and safety of fecal microbiota transplantation in reducing recurrence of colorectal adenomas after endoscopic resection.
NCT06405113	Fecal Microbiota Transplantation Combined with SOX and Sintilimab as First-line Treatment for Advanced Gastric Cancer: A Prospective, Multicenter, Randomized, Double-blind, Placebo-controlled Study (FMT-JSNO-01)	Gastric Cancer	Combination Product: Fecal Microbiota Transplantation (FMT)+chemotherapy+immunotherapy	The investigators plan to initiate a prospective, multicenter, randomized, double-blind, placebo-controlled phase II study, recruiting 198 patients with advanced gastric/gastroesophageal junction adenocarcinoma who have not received prior treatment. Randomly divided into two groups, one group is the group of fecal microbiota transplantation (FMT) + SOX+Sintilimab, and the other group is the group of SOX+Sintilimab. Compare the 2-year OS rates of the two groups to verify whether the addition of FMT to first-line treatment can improve the prognosis of gastric cancer patients.
NCT06346093	A Prospective, Randomized Placebo Controlled Trial of Fecal Microbiota Transplantation in Patients with Advanced Gastric Cancer	Advanced Gastric Cancer	Procedure: Fecal Microbiota Transplantation Capsules Procedure: Placebo	This study is a randomized, double-blind and placebo-controlled study. The purpose of this study is to evaluate the efficacy and safety of FMT capsules combined with chemotherapy and anti-PD-L1 therapy in the advanced gastric cancer.
NCT06610097	Impacts of Diet, Activity, and Mood on a Dynamic Gut Microbiota During Treatment for Triple-negative Breast Cancer	Breast Cancer	Nutritional Counseling	In this prospective randomized controlled study, the investigators propose to recruit up to 30 early-stage triple-negative breast cancer patients to randomize to a personalized nutritional intervention of a high-fiber diet coached by a registered dietician versus educational handout alone during neoadjuvant treatment. The investigators propose to study the gut microbiota through stool sample analysis among early-stage triple-negative breast cancer patients undergoing neoadjuvant (i.e., before surgery) chemotherapy +/- immunotherapy.
NCT06403111	Fecal Microbiota Transplantation Combined with Platinum-based Doublet Chemotherapy and Tislelizumab as First-line Treatment for Driver-gene Negative Advanced Non-Small Cell Lung Cancer: Study Protocol for a Prospective, Multicenter, Single-arm Exploratory Trial	Non-small Cell Lung Cancer	Combination Product: Fecal Microbiota Transplantation +c hemotherapy + immunotherapy	This study plans to reconstruct intestinal microecology through fecal microbiota transplantation, and combine with standard first-line therapy to enhance the anti-tumor immune effect at the same time, thereby extending the progression-free survival of patients and improving the prognosis of patients.
NCT05690048	Fecal Microbiota Transfer in Liver Cancer to Overcome Resistance to Atezolizumab/Bevacizumab (FLORA)	Hepatocellular Carcinoma	Drug: Fecal microbiota transfer Drug: Vancomycin Oral Capsule Drug: Atezolizumab + Bevacizumab	The interventional, randomized, placebo-controlled, single blind phase II-trial FLORA will assess safety and immunogenicity of fecal microbiota transfer in combination with standard of care immunotherapy in advanced hepatocellular carcinoma in a parallel group design.
NCT06218602	Pilot Trial of Fecal Microbiota Transplantation for Lymphoma Patients Receiving Axicabtagene Ciloleucel Therapy.	Lymphoma	Drug: Fecal Microbiota Transplantation Procedure: Chemotherapy Procedure: CAR-T Therapy	To determine if adding treatment with fecal microbiota transplantation is effective at treating gut-related side effects of antibiotic treatment in participants who are receiving standard therapy with anti-CD19 chimeric antigen receptor T-cell therapy.
NCT06486220	PFLL Combined With PD-1 Antibody with or Without FMT for Oligometastatic NPC, a Phase III, Open, Randomized Clinical Trial.	Nasopharyngeal Carcinoma	Drug: Intestinal bacteria freeze-dried powder capsules	There is a correlation between gut microbiota and immunotherapy reactivity, and regulating gut microbiota through FMT can prevent primary resistance to immune checkpoint inhibitors and further improve the effectiveness of tumor immunotherapy. Therefore, on the basis of previous studies, this study intends to explore whether intestinal flora transplantation can improve the anti-tumor efficacy of low-dose long term 5-FU pumping (“old fire soup”) therapy combined with immunotherapy and reduce the occurrence of toxic side effects in patients with metastatic nasopharyngeal carcinoma.
NCT05669846	Phase II Feasibility Study of Healthy Donor FMT and Pembrolizumab in Relapsed/Refractory (R/R) PD-L1 Positive NSCLC	Non-Small Cell Lung Cancer	Drug: Healthy Donor Fecal Microbiota Transplant Drug: Pembrolizumab	This study is to determine if Healthy Donor FMT improves the body’s ability to fight cancer in patients with relapsed/refractory PD-L1 Positive NSCLC.
NCT06643533	Safety and Efficacy of Fecal Microbiota Transplantation in Reversing Drug Resistance in Patients With Intermediate-advanced Unresectable Hepatocellular Carcinoma Undergoing Transcatheter Arterial Chemoembolization Combined With Lenvatinib Plus Sintilimab.	Hepatocellular Carcinoma Nonresectable	Other: Fecal Microbiota Transplantation	This study aims to evaluate the safety and efficacy of fecal microbiota transplantation in reversing drug resistance to the triple therapy regimen in patients with unresectable hepatocellular carcinoma.
NCT06393400	An Open-label, Single-arm, Phase 1 Study of the Combination of FMT and Gemcitabine with Nab-paclitaxel As First-line Therapy in Patients with Advanced Pancreatic Ductal Adenocarcinoma.	Unresectable or Metastatic Advanced Pancreatic Ductal Adenocarcinoma	Drug: Fecal Microbiota Transplantation Drug: PEG3350 Drug: Gemcitabine Drug: nab-Paclitaxel	To confirm the safety of combining oral fecal microbiota transplantation with gemcitabine and nab-paclitaxel chemotherapy as first line treatment in patients with unresectable or metastatic pancreatic ductal adenocarcinoma.
NCT06801665	Fecal Microbiota Transplantation Combined with QL1706, Bevacizumab, and XELOX As First-line Treatment for Advanced MSS-type Colon Cancer with Liver Metastasis: a Prospective, Multicenter, Single-arm Phase II Study	Colon Cancer Liver Metastases	Combination Product: FMT + QL1706+Bevacizumab+XELOX	The investigators plan to initiate a prospective, multicenter, phase II study, recruiting 30 patients with advanced colon cancer patients with liver metastasis who have not received prior treatment. This study plans to reconstruct intestinal microecology through fecal microbiota transplantation, and combine with QL1706+Bevacizumab+XELOX to enhance the anti-tumor immune effect at the same time, thereby improving the prognosis of colon cancer patients with liver metastasis.

**Table 5:** Clinical trials of cancer therapy utilizing microbiota depletion

NCT number	Official title	Cancer type	Intervention	Research purposes
NCT06627270	A Phase II Single-arm Cohort Study Establishing the Effect of Antibiotic Treatment on Intratumoral Bacteria in Surgical Patients with Oral Cancer	Oral Squamous Cell Carcinoma Oral Cancer Head and Neck Cancer Head and Neck Carcinoma	Drug: Metronidazole Other: Chlorhexidine	The goal of this phase II single arm clinical study is to evaluate the effect of antibiotics (metronidazole) and oral chlorhexidine (CHX) in reducing the bacteria load within tumors of patients undergoing surgery for oral cancer.
NCT04875728	Evaluating the Impact of Perioperative Antibiotic Prophylaxis on the Microbiome in Patients with Cutaneous Malignancy	Clinical Stage I Cutaneous Melanoma AJCC v8 Clinical Stage IA Cutaneous Melanoma AJCC v8 Clinical Stage IB Cutaneous Melanoma AJCC v8 Clinical Stage II Cutaneous Melanoma AJCC v8	Drug: Cefazolin Procedure: Resection	This phase I trial investigates the impact of cefazolin before surgery on the microbiome in patients with stage I-II melanoma.
NCT05777603	Phase I Study of Aerosolized Antibiotics and Pembrolizumab in Advanced Non-Small Cell Lung Cancer	Advanced Non-Small Cell Lung Cancer	Drug: aerosolized aztreonam Drug: aerosolized vancomycin Drug: pembrolizumab	To test 2 inhaled antibiotics (aztreonam and vancomycin), combined with a standard cancer treatment, in people with NSCLC.
NCT06793137	Phase II Trial for Intestinal Microbiome Modulation with Antibiotics in the Neoadjuvant Treatment of Locally Advanced Rectal Cancer	Rectal Cancer, Adenocarcinoma	Drug: Metronidazole	Intestinal microbiota studies have shown that an overpopulation of certain anaerobic bacteria is generally associated with poorer treatment response. No study has attempted to intervene in the gut microbiota to increase the complete response rate in rectal cancer. The proposal of the investigators aims to modulate the intestinal microbiota through a phase 2 clinical trial, with the use of metronidazole as the intervention.
NCT02399059	Effect of Peritoneal Lavage with Clindamycin-Gentamicin Solution During Elective Colorectal Cancer Surgery on the Oncologic Outcome	Colorectal Tumors	Procedure: Antibiotic lavage Procedure: Normal saline lavage	Antibiotic lavage reduces bacterial contamination and decreases SSI infection rate. SSI leads to an immunocompromised situation, leaving unattended the neoplasm. It has been described that SSI may result in a worse oncologic outcome.
NCT02407119	Effect of Helicobacter Pylori Eradication on Glandular Atrophy and Metachronous Cancer in Patients Undergoing Endoscopic Mucosal Resection for Gastric Cancer	Helicobacter Pylori Infection Early Gastric Cancer Endoscopic Resection	Drug: 7-day *H. pylori* eradication Omeprazole or Rabeprazole, Clarithromycin, Amoxicillin Drug: Placebo, Omeprazole or Rabeprazole, Clarithromycin	This study evaluates whether *Helicobacter pylori* eradication improves precancerous lesions including glandular atrophy and intestinal metaplasia as well as metachronous cancers or dysplasias after endoscopic mucosal resection for gastric cancer.

## Conclusion and perspective

The cancer-associated microbiota is integral to the tumor ecosystem and is significantly involved in tumor progression, metabolism, and the immune response. This review examines the evolution of research on microbiota‒cancer interactions, aiming to elucidate the roles and mechanisms by which various microbes contribute to tumor development. They also emphasize their involvement in critical metabolic processes, including glycolysis, lipid metabolism, and amino acid metabolism, and explore their complex interactions with immune cells across various tumor types. Furthermore, these microbiota serve as sensors of the TME and predictors of drug response and prognosis and are also distinct contributors to tumor progression.^321,381^ Their endogenous localization within tumors positions them as promising candidates for drug delivery vectors, enabling them to navigate the TME and effectively target cancer cells. Consequently, the cancer-associated microbiota can be regarded as a drug target, diagnostic and prognostic indicator, and adjunct to existing treatment regimens.^381^ The role of microbes in cancer is anticipated to become a central theme in cancer research over the next decade. Some tumor-intrinsic bacteria exhibit anticancer properties, suggesting the potential for direct transplantation into the TME to enhance the immune response and improve therapeutic efficacy. The species, abundance, and localization of these bacteria or fungi are significant determinants of the outcomes of anticancer therapies. In addition, their symbiotic coexistence with the host mitigates certain safety concerns. Further research should focus on elucidating the specific molecular mechanisms of microbes in tumors, identifying new therapeutic targets and biomarkers, and developing personalized treatment plans, thereby facilitating precision medicine and improving patient prognosis.

Several challenges persist despite promising advancements in the study of tumor-associated microorganisms. One significant issue is the technical limitation encountered in metagenomic sequencing, particularly in tumors with low microbial biomass, which impedes the acquisition of complete microbial genomes.^321^ The diverse roles of bacteria across various tumors may also complicate the ability to generalize their functions. For example, the origin and role of bacteria in the gut microbiome and CRC remain ambiguous owing to their low abundance and potential dual roles, which complicate their study. Another challenge lies in the reliance on animal models in experiments, as these models differ significantly from humans in genetic background and disease heterogeneity, and there is still a lack of result validation in human patients. The low abundance of bacteria also increases the risk of false positives due to sample contamination, leading to potential misinterpretations of microbial types and functions. A solution to address these issues may be to improve the experimental design by establishing more control groups, developing advanced data analysis and decontamination algorithms, and employing refined technical methods. Simultaneously, live bacterial products present additional challenges in clinical application, as they do not adhere to typical pharmacokinetic and dose‒response relationships, complicating the determination of optimal dosing and administration schedules. In addition, live microbiota products carry infection risks and may contaminate the medical environment. Therefore, significant efforts are needed to effectively transform the therapeutic potential of microorganisms into clinical practice.

To date, the interaction between the host and microbiome is complex and remains incompletely understood. Large-scale, cross-cancer, longitudinal studies are needed to elucidate this relationship in the future. These studies should aim to investigate the mechanisms by which microorganisms infiltrate tumor cells, integrate into host cell systems, and interact with both the immune system and the TME. Such research is essential for harnessing the full potential of the microbiota in the diagnosis and treatment of cancers. We anticipate that these advancements will promote a paradigm shift in cancer therapy to transition from harmful and nonselective treatments to more precise and convenient approaches. In the foreseeable future, comprehensive investigations into the role of the microbiota in cancers will lay a solid foundation for the development of novel cancer therapies.
